# High-Resolution Analysis of Coronavirus Gene Expression by RNA Sequencing and Ribosome Profiling

**DOI:** 10.1371/journal.ppat.1005473

**Published:** 2016-02-26

**Authors:** Nerea Irigoyen, Andrew E. Firth, Joshua D. Jones, Betty Y.-W. Chung, Stuart G. Siddell, Ian Brierley

**Affiliations:** 1 Division of Virology, Department of Pathology, University of Cambridge, Cambridge, United Kingdom; 2 Department of Plant Sciences, University of Cambridge, Cambridge, United Kingdom; 3 Department of Cellular and Molecular Medicine, University of Bristol, Bristol, United Kingdom; University of Maryland School of Medicine, UNITED STATES

## Abstract

Members of the family *Coronaviridae* have the largest genomes of all RNA viruses, typically in the region of 30 kilobases. Several coronaviruses, such as *Severe acute respiratory syndrome-related coronavirus* (SARS-CoV) and *Middle East respiratory syndrome-related coronavirus* (MERS-CoV), are of medical importance, with high mortality rates and, in the case of SARS-CoV, significant pandemic potential. Other coronaviruses, such as *Porcine epidemic diarrhea virus* and *Avian coronavirus*, are important livestock pathogens. Ribosome profiling is a technique which exploits the capacity of the translating ribosome to protect around 30 nucleotides of mRNA from ribonuclease digestion. Ribosome-protected mRNA fragments are purified, subjected to deep sequencing and mapped back to the transcriptome to give a global “snap-shot” of translation. Parallel RNA sequencing allows normalization by transcript abundance. Here we apply ribosome profiling to cells infected with *Murine coronavirus*, mouse hepatitis virus, strain A59 (MHV-A59), a model coronavirus in the same genus as SARS-CoV and MERS-CoV. The data obtained allowed us to study the kinetics of virus transcription and translation with exquisite precision. We studied the timecourse of positive and negative-sense genomic and subgenomic viral RNA production and the relative translation efficiencies of the different virus ORFs. Virus mRNAs were not found to be translated more efficiently than host mRNAs; rather, virus translation dominates host translation at later time points due to high levels of virus transcripts. Triplet phasing of the profiling data allowed precise determination of translated reading frames and revealed several translated short open reading frames upstream of, or embedded within, known virus protein-coding regions. Ribosome pause sites were identified in the virus replicase polyprotein pp1a ORF and investigated experimentally. Contrary to expectations, ribosomes were not found to pause at the ribosomal frameshift site. To our knowledge this is the first application of ribosome profiling to an RNA virus.

## Author Summary

Ribosome profiling is emerging as a powerful technique to monitor translation in living cells at sub-codon resolution. It has particular applicability to virology, with the capacity to identify viral mRNAs that are being translated during infection and to provide new insights into virus gene expression, regulation and host-virus interactions. In this work, we carried out the first ribosome profiling analysis of an RNA virus, using as a model system the murine coronavirus strain MHV-A59, a betacoronavirus in the same genus as the medically important SARS-CoV and MERS-CoV. Parallel ribosome profiling and RNA sequencing of infected-cell time points was performed during the course of MHV replication in mouse tissue culture cells and used to determine virus gene expression kinetics and the relative translational efficiencies of virus and host mRNAs. The sensitivity and precision of the approach permitted us to uncover several unanticipated features of coronavirus translation, giving insights into ribosomal frameshifting, ribosome pausing, and the utilisation of short, potentially regulatory, upstream open reading frames. We also identified some challenges associated with the technique that are of general relevance to the ribosome profiling technique and developed bioinformatic strategies to address these.

## Introduction

Members of the family *Coronaviridae* have the largest genomes of all RNA viruses, typically in the region of 30 kilobases (kb). Several coronaviruses, including SARS-CoV and MERS-CoV, are of medical importance, with high mortality rates and, in the case of SARS-CoV, significant pandemic potential. Other coronaviruses, such as *Porcine epidemic diarrhea virus* and *Avian coronavirus*, are important livestock pathogens. Coronavirus infections are frequent in bats and other mammals [1] and interactions between humans and non-human animal populations presents a constant risk of new zoonotic outbreaks [2]. Recent findings also indicate an evolutionary origin of the established human coronavirus species, *Human coronavirus 229E* in hipposiderid bats [3].

The family *Coronaviridae* is divided into the subfamilies *Coronavirinae* and *Torovirinae*. *Torovirinae* includes the genera *Bafinivirus* and *Torovirus*, infecting fish and mammals respectively, while *Coronavirinae* includes the genera *Alphacoronavirus*, *Betacoronavirus*, *Gammacoronavirus* and *Deltacoronavirus*, commonly infecting mammals and birds. SARS-CoV and MERS-CoV are members of the genus *Betacoronavirus*. Therefore, a useful model for these two viruses, especially with regard to their structure and replication, is *Murine coronavirus*, a betacoronavirus that is commonly referred to as mouse hepatitis virus (MHV).

**Fig 1:**
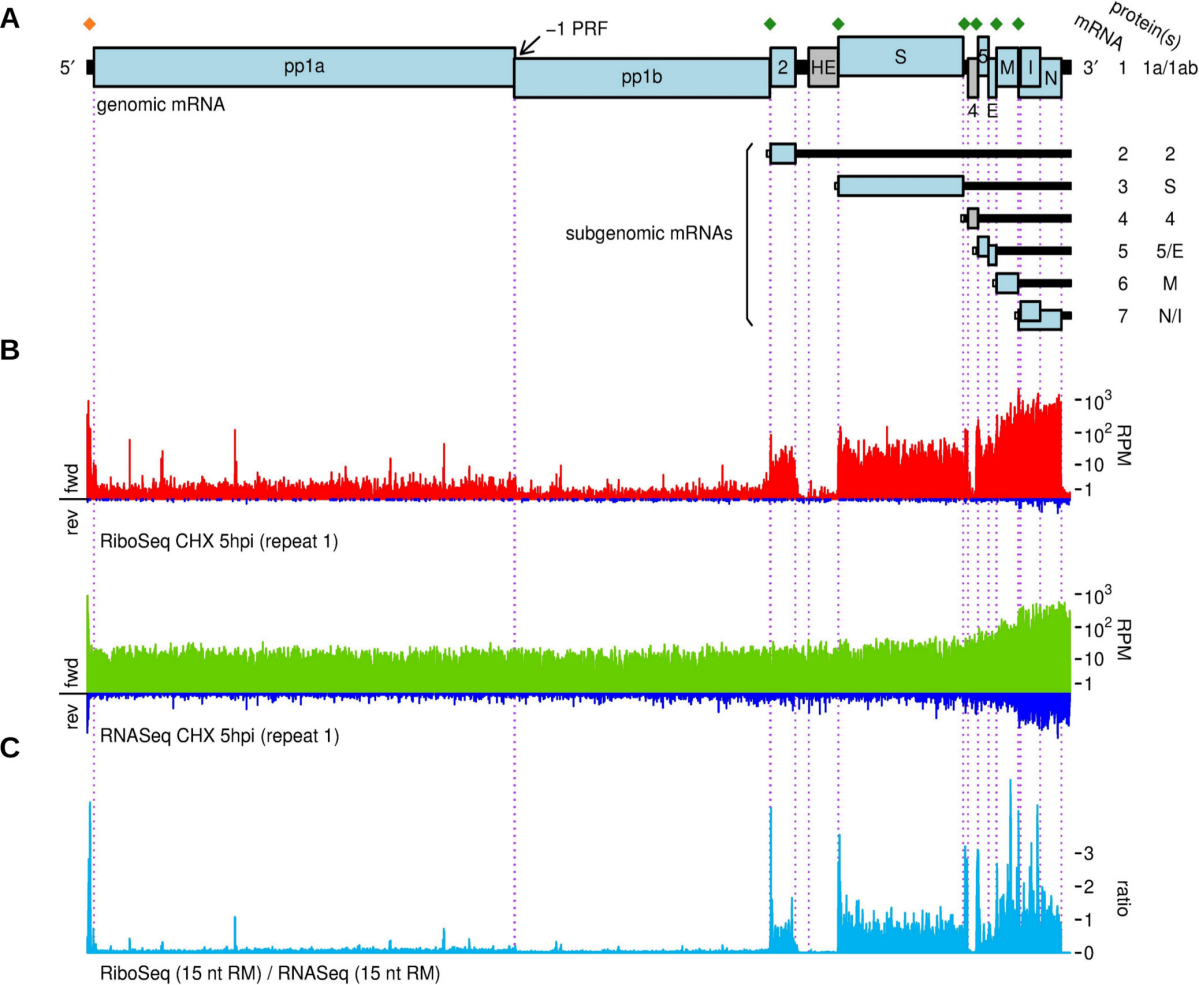
MHV RNA synthesis and translation. **(A)** Transcript map of the 31335-nt MHV-A59 genome. Polyproteins pp1a and pp1b are translated from the genomic RNA, with pp1b being expressed as a transframe fusion with pp1a (i.e. pp1ab) via −1 programmed ribosomal frameshifting (−1 PRF). The 3′ ORFs are expressed from a series of subgenomic RNAs produced during infection. Each subgenomic RNA contains a 5′ leader sequence that is identical to the 5′ leader of the genome, appended via polymerase “jumping” between body transcription regulatory sequences (TRSs) (green diamonds) and the leader TRS (orange diamond) during negative-strand synthesis. Due to mutations present in this laboratory-adapted strain, the hemagglutinin-esterase and ORF4 gene fragments (HE and 4; grey boxes) are not expected to be translated. **(B)** RiboSeq CHX (red) and RNASeq (green) densities at 5 h p.i. (repeat 1) in reads per million mapped reads (RPM). Read densities are plotted on a log(1+x) scale to cover the wide range in expression across the genome. Histograms show the positions of the 5′ ends of reads with a +12 nt offset to map (for RPFs) approximate P-site positions. Negative-sense reads are shown in dark blue below the horizontal axis. **(C)** The positive-sense RiboSeq/RNASeq ratio, after first applying a 15-nt running mean (RM) filter to each individual distribution.

Like all coronaviruses, MHV has a monopartite, positive-sense, single-stranded RNA genome (gRNA) (Fig 1A). The 5′ two thirds of the genome contains two long open reading frames (ORFs), ORF1a and ORF1b, which encode the replicative proteins. These ORFs are expressed as two polyproteins pp1a and pp1ab, where pp1ab is a “transframe” fusion of the ORF1a and ORF1b products, produced via −1 programmed ribosomal frameshifting (−1 PRF) [4, 5]. Polyproteins pp1a and pp1ab are proteolytically cleaved by virus-encoded proteases, PLP1 and PLP2 (in nsp3) and 3CL (nsp5) to produce the non-structural proteins nsp1 to nsp16. The 3′ third of the genome contains ORFs that encode structural proteins and accessory proteins. These ORFs are translated from a series of subgenomic mRNAs (mRNAs 2 to 7) produced during virus infection. Each subgenomic mRNA is identical to a 3′-coterminal region of the virus genome with the exception of a 65 nucleotide (nt) leader sequence at the 5′ end that is identical to the 5′ end of the gRNA. These leader sequences are added (as a reverse complement) during synthesis of subgenomic negative-sense templates that then give rise to the positive-sense mRNAs. The process of discontinuous transcription during negative-strand RNA synthesis takes place via polymerase “jumping” at specific “transcription regulatory sequences” (TRSs) on the gRNA template. In MHV, mRNAs 2 to 7 encode, respectively, proteins 2 and haemagglutinin-esterase (HE), spike (S), protein 4, protein 5 and envelope (E), membrane (M), nucleocapsid (N) and internal protein (I), with mRNAs 5 and 7 being functionally bicistronic (Fig 1A) [6]. In the laboratory-adapted strain (MHV-A59) employed in the present study, however, expression of HE and protein 4 is defective.

Ribosome profiling is an emerging methodology that facilitates global mapping of the positions of translating ribosomes on the transcriptome, defining at the codon level the extent to which individual mRNAs species are engaged in protein synthesis [7–9]. The technique exploits the knowledge that translating ribosomes can protect from RNase digestion a defined fragment of mRNA of around 28–30 nt in length [10]. In ribosome profiling, often referred to as RiboSeq, cells are lysed under conditions optimised to minimise further ribosome movement (addition of translation inhibitors, rapid freezing), the lysate is treated with ribonuclease (often RNase 1) to degrade regions of mRNAs that are not physically protected, and the ribosomes harvested on sucrose gradients or through a sucrose cushion. The ribosome pellet is de-proteinized, the ribosome-protected fragments (RPFs) harvested by elution from a polyacrylamide gel, ligated to adapters, subjected to RT-PCR, deep sequenced and mapped back to the genome. This analysis reveals the location and abundance of ribosomes on mRNAs with up to single-nucleotide precision. The corresponding transcriptome is also determined from the same lysate: total RNA is harvested, fragmented, cloned and sequenced to generate a parallel RNA sequencing (RNASeq) library.

Ribosome profiling has been applied to a variety of cellular organisms to address a range of questions in translational control and global gene expression [9, 11–16]. Also, it has been employed in the study of the replication of large DNA viruses; namely, human cytomegalovirus [17–18], Kaposi's sarcoma-associated herpesvirus [19], herpes simplex virus 1 [20], vaccinia virus [21], and bacteriophage lambda [22], providing insights into the temporal regulation of gene expression in these viruses and identifying numerous previously unrecognized translated ORFs, including novel protein-coding ORFs and short regulatory uORFs.

In this paper, we describe the first analysis of RNA virus replication and gene expression by ribosome profiling (and parallel RNASeq), using MHV as a model system. The data obtained allowed us to determine the time course of virus positive and negative-sense RNA production, as well as the translation of each of the virus genes, the expression of short and/or previously unannotated ORFs, and the efficiency of −1 PRF. We also investigated early time points of infections at high multiplicity to visualise the translation of input genomes. The profiling data also revealed examples of prominent ribosomal pausing within the coding regions for nsp3 and nsp6. Nsp3 ribosomal pausing was confirmed in *in vitro* translation experiments. Surprisingly, we found that ribosomes do not pause appreciably during −1 PRF, arguing against a requirement for pausing in frameshifting. This study also provides insights into the challenges associated with the profiling of RNA viruses and suggests strategies that may prove beneficial in future studies.

## Results

### Ribosome profiling of MHV-infected cells

To study the kinetics of virus RNA and protein synthesis in a single cycle of virus replication, we performed two independent biological repeats (repeats 1 and 2) of an MHV infection time course in which murine 17 clone 1 cells (17Cl-1) were infected with recombinant MHV-A59 at a multiplicity of infection (MOI) of 10 and cells harvested at 1, 2.5, 5 and 8 h post-infection (p.i.), with mock-infected cells harvested at 1 and 8 h. For all time points, two dishes were prepared and, immediately prior to harvesting, cells were treated with cycloheximide (CHX) alone, or harringtonine (HAR) then CHX (as detailed in Materials and Methods), for analysis of elongating (CHX) and initiating (HAR) ribosomes, respectively. Subsequently, RiboSeq (CHX), RiboSeq (HAR) and RNASeq (CHX only) libraries were prepared for each time point, deep sequenced and reads mapped to host and virus sequences (see Materials and Methods). The composition of each library is summarised in S1A Table and S1 Fig.

### Virus gene expression at 5 h p.i.

As an example of the data provided by our experimental strategy, Fig 1B shows the density of RiboSeq CHX and RNASeq reads mapping to the virus genome at 5 h p.i. In general, there is a 5′ to 3′ increasing gradient in total ribosome density, with the N ORF being expressed at the highest level, the M, 5, S and 2 ORFs at intermediate levels, and ORFs 1a and 1b at the lowest levels. As expected, very little ribosome density was observed within the defective genes HE and 4. The step reduction in RiboSeq density between ORF1a and 1b reveals the proportion of ribosomes that terminate at the ORF1a stop codon instead of frameshifting into ORF1b. In contrast, RNASeq density is essentially constant across ORFs 1a and 1b, and then steadily increases 5′ to 3′, reflecting the cumulative density summed over the genomic RNA and 3′-coterminal subgenomic transcripts. Extra RNASeq density in the 5′ UTR reflects the 5′ leader sequence that is present on all subgenomic transcripts as well as the genomic RNA. RiboSeq density was also observed in the 5′ leader, although not corresponding to known coding regions (see below).

Negative-sense virus RNA is present at much lower amounts than positive-sense RNA, but follows roughly the same expression patterns, including high density in the (anti)-leader region, consistent with discontinuous transcription occurring during negative-sense RNA synthesis [23]. Low levels of negative-sense RiboSeq reads were also observed but these had length distributions that did not match typical RPF length distributions (see below). Thus, these are unlikely to derive from ribosomes loading onto negative-sense RNAs (e.g. non-specifically onto uncapped, possibly degraded virus-derived RNAs). Instead, they may derive from low amounts of RNA non-specifically co-sedimenting with ribosomes (see below).

Since the RiboSeq analysis represents the product of transcript abundance and translation efficiency, we also plotted the RiboSeq/RNASeq ratio along the genome (Fig 1C). This ratio was substantially lower in ORF1a and ORF1b than in the 3′ coding ORFs (except the defective genes HE and 4), which may indicate that a substantial proportion of genomic RNA is not being translated (e.g. sequestered in replication-transcription complexes [RTCs] or destined for packaging) or that genomic RNA intrinsically has a relatively low translation efficiency. Note, however, that this simple calculation ignores the fact that RNASeq density is present for all ORFs on a transcript whereas RiboSeq density is only present for the translatable ORFs (normally the 5′ proximal ORF). This discrepancy is accounted for in the more detailed analysis of translation efficiencies below.

**Fig 2:**
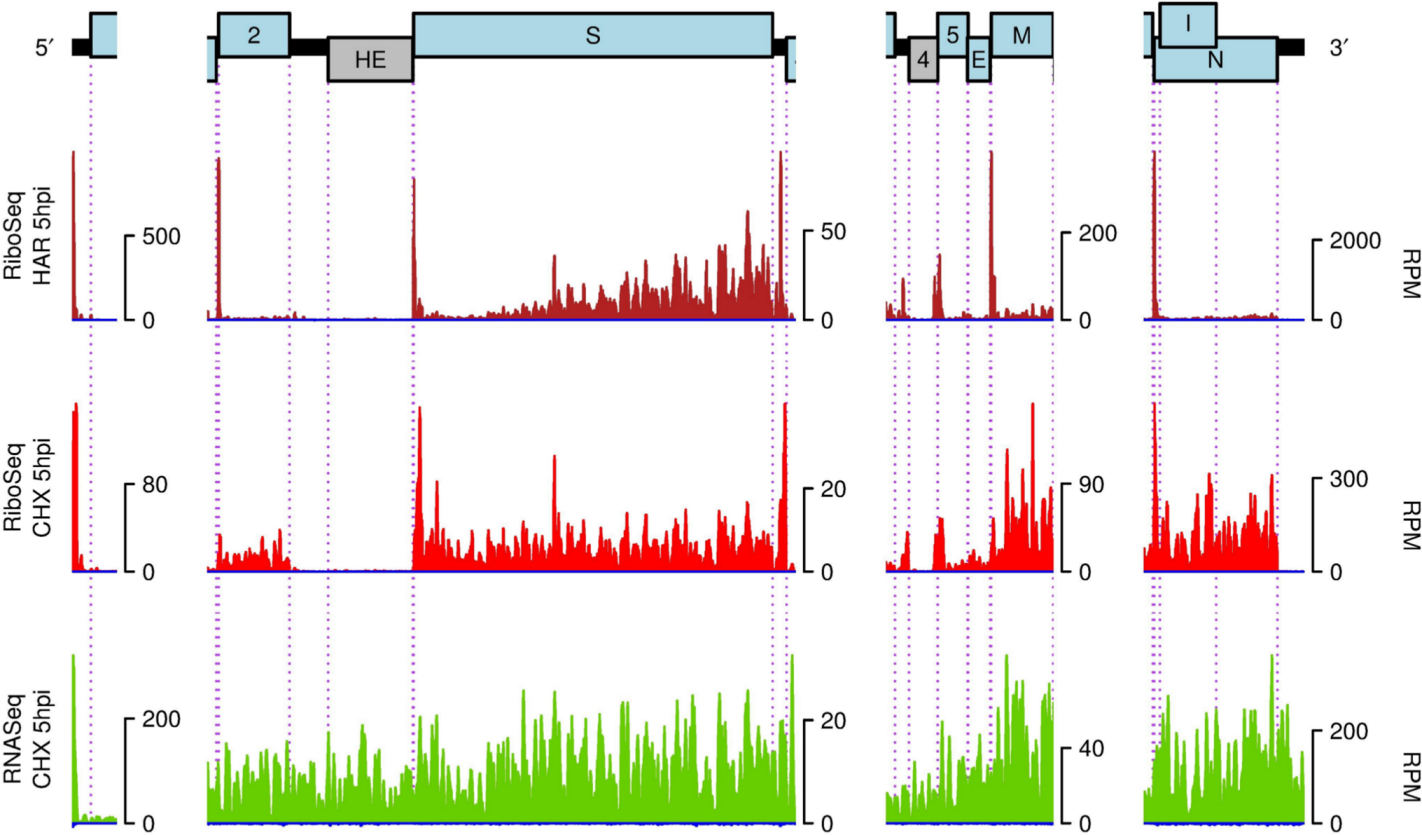
RNA synthesis and translation in the 5′ UTR and 3′ ORF regions. RiboSeq HAR (dark red), RiboSeq CHX (red) and RNASeq (green) densities at 5 h p.i. (repeat 1) in reads per million mapped reads (RPM), smoothed with a 15-nt running mean filter and plotted on a linear scale. Histograms show the positions of the 5′ ends of reads with a +12 nt offset to map (for RPFs) approximate P-site positions. Negative-sense reads are shown in dark blue below the horizontal axis.

Fig 2 shows enlarged views of the virus transcript 5′ UTR and 3′ ORFs with linear scales optimized separately for each region. This analysis shows that there is significant variability in the RNASeq read depth within a transcript, which we ascribe to biases such as fragmentation bias, PCR bias and ligation bias. Similarly, variability in the RiboSeq data within a CDS may be partly due to nuclease bias, PCR bias and ligation bias but also reflects real variations in ribosome progressivity. The depth of RNASeq reads in the 5′ UTR is similar to that of the N ORF, reflecting that the major contribution to 5′ leader sequence comes from mRNA7. Peaks in the RiboSeq HAR data highlight the canonical translation initiation sites of the 2, S, 5, M and N ORFs. In the same dataset, the ORF1a/1ab initiation peak is dwarfed by RPFs in the 5′ leader (presumably mostly coming from mRNA7; see below). It should be noted that HAR arrests ribosomes at initiation, but not during elongation thus allowing elongating ribosomes to run-off. However, in these samples it is apparent that elongating ribosomes have not yet cleared the S ORF.

### Assessment of data quality

We considered it important to assess the quality of the datasets that were obtained by our experimental strategy. For RPFs derived from non-organellar ribosomes of eukaryotic organisms, mapping of the 5′ end positions to coding sequences (CDSs) characteristically reflects the triplet periodicity (herein referred to as “phasing”) of translational decoding [7]. Good phasing within datasets is beneficial in assigning ORFs with confidence, particularly if such ORFs are very short or overlap. The extent of phasing can vary between protocols and libraries due, presumably, to variation in the efficiency of RNase I (or other nuclease) trimming or other factors. S2 Fig (repeat 1) and S3 Fig (repeat 2) show, for each library, histograms of the codon positions to which the 5′ ends of host mRNA reads map for different read lengths. The RiboSeq libraries show excellent phasing with the majority of RPF 5′ ends mapping to the first codon position. Conversely, and as expected, the 5′ ends of RNASeq reads had a nearly uniform distribution between the three possible codon positions. The RiboSeq read length distributions were typically sharply peaked at around 29 nt consistent with other analyses [8], while those of RNASeq were much broader, consistent with a length distribution set by the size of the gel slice excised during purification of fragmented RNA in the RNASeq protocol (approximately 28–34 nt).

S4 Fig shows the distribution of host mRNA RPF 5′ ends relative to initiation and termination codons, summed over all host mRNAs in each of the RiboSeq libraries. For all samples, a discrete peak in RPF abundance was observed just upstream of the initiation site. As noted previously, the peak is probably largely a result of drug treatment—either HAR which specifically arrests initiating ribosomes, or CHX which arrests elongating ribosomes but allows ribosomes to continue to accumulate at initiation sites [8]. This peak corresponds to the 5′ ends of RPFs derived from initiating ribosomes with the AUG codon in the ribosomal P-site, and allows calibration of the offset between the RPF 5′ end and RPF P-site position, which, for these libraries, is normally 12 nt (e.g. S5 Fig). For many samples, a discrete peak was also observed 15 nt upstream of the stop codon, corresponding to ribosomes pausing during termination (with the stop codon in the ribosomal A-site). The presence of this peak appears to be subject to minor variation in sample preparation as it was not consistent between repeats (cf. repeat 1 and repeat 2, RiboSeq CHX mock 1 h in S4 Fig). In contrast to [24], we believe that the clear spike four codons downstream of the initiation peak is an artefact of ligation bias (and potentially also other biases): every read mapping to this position begins with 5′-AUG (thus compounding any ligation preferences), whereas reads that map to the initiation peak have different 5′ and 3′ ends in different mRNAs (thus averaging out any ligation preferences). For 30-nt reads, a trough was also apparent four codons upstream of the termination peak (S5 Fig); this corresponds to reads that all end in UAG-3′, UAA-3′ or UGA-3′, and again is likely to be an artefact of ligation bias. Peaks at the start and stop codons were also apparent for RNASeq data, corresponding to reads with 5′ ends aligning to the A of AUG and the middle nucleotide of the stop codon, respectively (S6 Fig, right); the latter is not visible in RiboSeq data due to low RiboSeq density in the 3′ UTR. A peak 12 nt upstream of the AUG (more noticeable in repeat 1 samples, S6 Fig, left) together with a very low level of phasing within the CDS (S6 Fig) likely represents a low level of contamination of RNASeq samples by RiboSeq samples, although the latter could potentially also be a result of codon usage bias, e.g. a preference for RNY codons [25], compounded with ligation biases.

Averaged over all host mRNAs, very few RPFs were observed in 3′ UTRs while a larger but still low level of RPFs were observed in 5′ UTRs (S4 Fig and S5 Fig). The latter may largely derive from translation of uORFs in various locations and phases with respect to the main ORF of each mRNA [8]. We also observed a remarkable perturbation in host cell translation at late time points (S4 Fig, lower panels—RiboSeq CHX, compare 5 and 8 h p.i. with 1 and 8 h mocks) that was not mirrored in RNASeq data (S6 Fig) and could be a consequence of cell stress [26–28]. This phenomenon and other host cell responses to virus infection will be discussed in future work.

We also addressed the issue of possible contamination during sample preparation as we expected that RNASeq and RiboSeq analysis of virus-infected cells may present some specific challenges. For example, late in infection, virus RNA can be produced at very high levels and extreme care is required to minimise cross contamination between late and early time-point libraries. Indeed, a comparison of read length distributions of host-derived RNA and virus-derived RNA revealed contamination of this type in some of our libraries, despite great care in processing experimental samples (S7 Fig and S8 Fig). For example, in the first biological repeat (S7 Fig), the virus and host length distributions in the 5 h p.i. RiboSeq CHX sample were almost identical. However, for the 1 and 2.5 h p.i. RiboSeq CHX samples, virus and host length distributions were dissimilar to each other but instead the virus length distribution resembled the RiboSeq CHX 5 h p.i. length distribution, suggesting contamination of virus RPFs from the later time-point sample into the earlier time points. The absolute amount of contamination was very low and would have little effect on host mRNA analyses; however, relative to the amount of virus RNA at 1 and 2.5 h p.i., it was significant. Contamination was also apparent for the 1 and 2.5 h p.i. RiboSeq HAR samples and the 1 h p.i. RNASeq sample. Similarly, the mock-infected controls each contained ~1000–2000 virus reads (cf. ~2–22 million at late time points of infection) (S1A Table). In the second biological repeat, the mock samples were evidently less contaminated, containing from only 0 to 55 virus reads each (S1A Table). Nevertheless, traces of contamination were still apparent in the 1 and 2.5 h p.i. RiboSeq CHX and 1 h p.i. RiboSeq HAR samples (S8 Fig). A different type of contamination was observed for the 8 h p.i. RiboSeq CHX sample in repeat 2. Here, the host read length distribution was broad compared to the virus read length distribution, and the host mRNA phasing was poor (S8 Fig and S3 Fig, respectively). This suggests that this sample is contaminated with RNASeq material from a sample containing little or no virus RNA, thus affecting the host mRNA length distribution but not the virus RNA length distribution. In subsequent discussions of the MHV profiling data, any samples suffering from contamination have been excluded, or subjected to appropriate caveats.

Another potential source of “contamination” in our experimental strategy is the problem of non-ribosomal ribonucleoprotein (RNP) complexes. For example, certain virus proteins have RNA binding properties and can associate with viral and, potentially, cellular RNA. These RNP complexes may co-sediment with ribosomes and lead to contamination of RiboSeq libraries. Such contamination may be revealed by unusually high read density in host mRNA 3′ UTRs (which normally have very low RPF occupancy) and differences in read length distributions [29]. S9 Fig and S10 Fig show length distributions for all libraries for reads mapping within 10 to 100 codons upstream (green; CDS) or downstream (orange; 3′ UTR) of CDS termination codons. In all RiboSeq libraries, the 3′ UTR read density was extremely low compared to the CDS read density (left plot of each pair). (It should be noted however that, as HAR enriches for initiating ribosomes, the above analysis is not well-suited to HAR samples.) For comparison, the RNASeq library 3′ UTR read density was typically ~80% of the CDS read density (that it is not 100% likely reflects the presence of transcripts with 3′ UTRs that are shorter than the annotated 3′ UTRs). Since the analysis is based on mapping to NCBI RefSeq mRNAs, a low level of 3′ UTR occupancy derives from genuine RPFs derived from coding exons in one splice form that have alternative mappings to the 3′ UTR in another splice form. Further, low levels of post-termination unrecycled 80S ribosomes may enter the 3′ UTR [30–32]. Thus, for mock infections, the 3′ UTR RiboSeq read length distributions largely matched those of the CDSs (S9 Fig and S10 Fig, 1 and 8 h mock CHX), albeit with some differences (e.g. a high-end tail) arising from unknown sources of contamination potentially including host protein:mRNA RNPs. While such contamination is expected to be present throughout the mRNA, it is more apparent in the 3′ UTR due to the much lower density of *bona fide* RPFs in this region.

For infected samples, the host mRNA 3′ UTR density for CHX samples was similar in magnitude (0.5–1.2%) to that of the mocks (0.7–0.9%), except for the 8 h p.i. time points where the 3′ UTR density was 2.9–6.3% of the CDS density (S9 Fig and S10 Fig). Consistent with the probable RNASeq contamination discussed above, the length profile of the 8 h p.i. CHX repeat 2 sample was broad for both the CDS and 3′ UTR regions. On the other hand, the length profile of the 8 h p.i. CHX repeat 1 sample was not qualitatively different from that of the mocks, suggesting that the increase in 3′ UTR occupancy might not simply be explained by virus-induced RNPs, but rather, or as well, by an increase in *bona fide* RPFs in the 3′ UTRs. A mechanism for the latter could be overloading of the host cell ribosome recycling factors (ABCE1 and any cofactors), allowing an increase in post-termination unrecycled 80S ribosomes entering the 3′ UTR [31].

If a proportion of late time-point contamination results from virus proteins interacting with mRNA to form RNPs, it may be significantly higher for virus RNA than for host mRNA, as virus proteins are likely to interact selectively with virus RNA; for example, through specific binding signals or via compartmentalization within the cell. Excess contamination in the virus RPF fraction may be gauged by comparing length distributions of reads mapping to virus positive-sense RNA with length distributions of reads mapping to host mRNA CDSs. Reassuringly, in all cases, the virus positive-sense RiboSeq reads showed a similar or even tighter length distribution at late time points than the host RiboSeq reads (S7 Fig and S8 Fig; 5 h.p.i and 8 h p.i., CHX and HAR). In contrast, as mentioned above, the small quantity of negative-sense virus reads in the RiboSeq samples had very different length distributions (S7 Fig and S8 Fig) indicating that they are unlikely to be true RPFs; such reads comprised <4% of virus reads for all RiboSeq samples, and <0.05% for the two 5 h p.i. CHX repeats.

### Time course of virus RNA and protein synthesis

**Fig 3:**
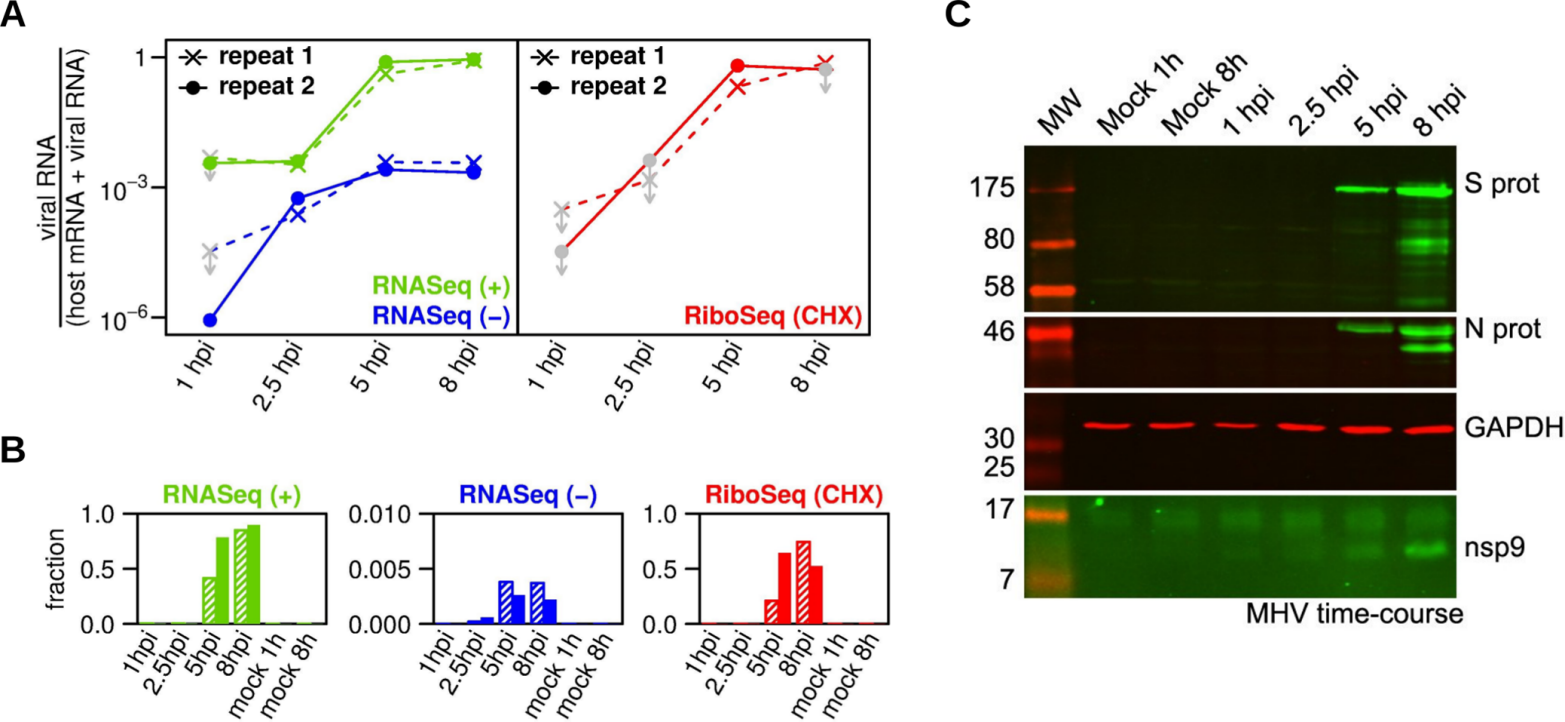
Time course of MHV total RNA synthesis and translation. **(A)** Time course of total virus RNA accumulation (left) and total virus translation (right). To normalize for differing library sizes, read counts are expressed relative to the total number of mapped virus RNA (positive and negative-sense) and mapped host mRNA reads for the library. Grey symbols with downward pointing arrows correspond to contaminated samples (see text) and represent upper bounds on the virus fraction. **(B)** Similar data represented on a linear scale; hatched bars—repeat 1, solid bars—repeat 2. **(C)** 17Cl-1 cells were infected with MHV-A59 (MOI 10) and harvested at 1, 2.5, 5 and 8 h p.i. Cell lysates were separated by 10% (for N and S westerns) or 17% (for nsp9 western) SDS-PAGE and immunoblotted using monoclonal anti-N, anti-S and anti-nsp9 sera. Molecular masses (in kDa) are indicated on the left. GAPDH was used as a loading control. All viral proteins were detected with a green fluorescent secondary antibody, and GAPDH with a red fluorescent secondary antibody.

Fig 3A shows a time course of the total amount of virus RNA expressed as a fraction of total virus RNA plus host mRNA, for both RiboSeq CHX and RNASeq samples. Samples with contamination (see above) could only be used to give upper bounds (grey symbols). Total virus translation as a fraction of total cellular translation increased 700 to 20,000-fold from 1 to 5 h p.i., while virus positive-sense RNA increased 80 to 200-fold over the same time period. In repeat 2, virus translation and RNA appeared to have reached a maximum by 5 h p.i., while infection progressed a little slower in repeat 1. From 1 h p.i. to 2.5 h p.i., the positive-sense RNA fraction remained roughly constant (presumably reflecting the input RNA) while the negative-sense RNA fraction grew from essentially negligible amounts to ~0.1% of total virus RNA and host mRNA (Fig 3A). At late time points, virus negative-sense RNA ceased to increase, whilst positive-sense virus RNA showed significant increases (Fig 3B). At the later time points, virus translation had reached ~50–75% of total cell translation and positive-sense virus RNA had reached ~80–90% of total virus RNA plus host mRNA. At the same time, negative-sense virus RNA represented ~0.3% of total virus RNA and host mRNA (Fig 3B). These findings are consistent with previous analysis of virus RNA synthesis in MHV-A59-infected cells [33]. Virus infection and the kinetics of viral protein expression over the time course were confirmed by western blot with antisera to the N, S and nsp9 proteins (Fig 3C).

**Fig 4:**
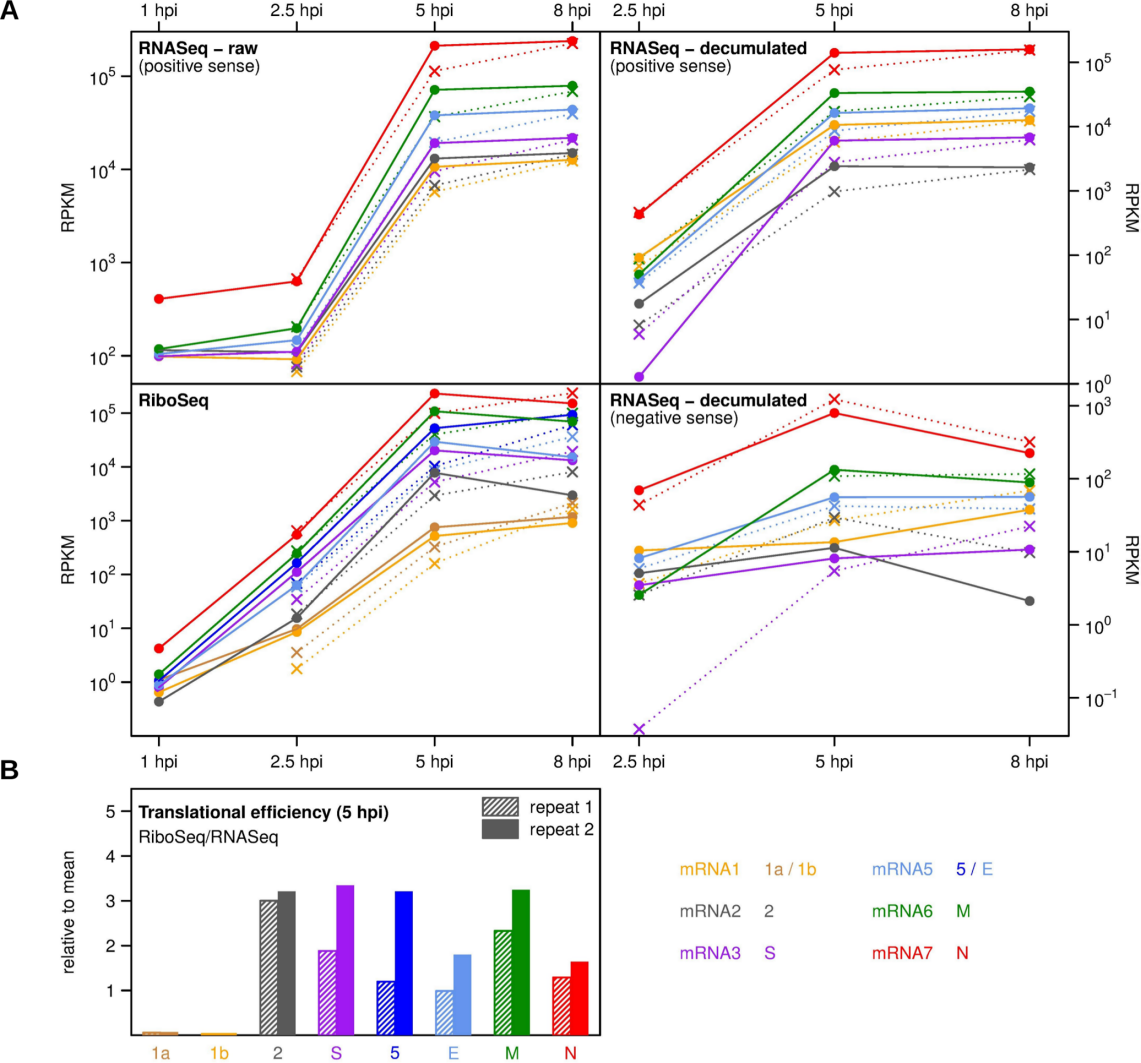
Time course of RNA synthesis and translation for different MHV genes. **(A)** Upper left: Time course of mean positive-sense raw RNASeq densities in each of six genome regions defined by the region between the TRS for a given mRNA and the next downstream TRS. Upper right: Estimated mean positive-sense RNASeq densities for each of mRNAs 1, 2, 3, 5, 6 and 7. Raw RNASeq densities represent the cumulative sum of densities for all mRNAs that cover a given genome region. Subtraction of the density for the immediately upstream inter-TRS region gives an estimate of the RNASeq density for a specific mRNA, herein referred to as the “decumulated” density. RNASeq densities for mRNA4 are omitted as it is not expressed at a sufficiently high level relative to mRNA3 to apply the “decumulation” procedure. Lower right: Estimated mean negative-sense RNASeq densities for each of the negative-sense subgenomic RNAs 2, 3, 5, 6, 7 and (anti)-gRNA. Lower left: Mean RiboSeq densities for each of ORFs 1a, 1b, 2, S, 5, E, M and N. The density for N includes any RPFs deriving from the overlapping I ORF. RiboSeq densities for the defective ORFs HE and 4 are omitted. Circles and solid lines correspond to repeat 2; crosses and dotted lines correspond to repeat 1. Due to low levels of reads and contamination (see text), values for 1 h p.i. and 2.5 h p.i. RiboSeq, and 1 h p.i. repeat 1 RNASeq should be considered as upper bounds and the 1 h p.i. repeat 1 RiboSeq values have been omitted. Densities are expressed in reads per kb per million mapped reads (RPKM). **(B)** Estimated translational efficiencies of different virus ORFs based on the quotient of the RiboSeq density for an ORF and the estimated positive-sense RNASeq density for the corresponding mRNA. Efficiencies are relative to mean host plus virus efficiencies and the calculation does not account for the presence of non-translated gRNA.

We also calculated the levels of transcription and translation for each virus ORF throughout the time course (Fig 4A). Note, again, that the data only provide upper bounds for the contaminated samples (as indicated in Fig 3). The particularly contaminated repeat 1 RiboSeq 1 h p.i. data are omitted from Fig 4A, while the upper bounds provided by the cleaner repeat 2 are included as they are likely to be more accurate. To calculate translation efficiencies, it is necessary to determine the amount of each virus transcript but, in the case of coronaviruses, raw RNASeq densities represent the cumulative sum of genomic RNA and all subgenomic transcripts. For example, for the N ORF, RNASeq density includes contributions from mRNAs 2 to 7 and gRNA. Thus, to calculate the amount of mRNA7, we subtracted the positive-sense RNASeq density in the region of mRNA6 upstream of the mRNA7 TRS from the density in the mRNA7 region. We then followed a similar procedure for all other mRNAs. The same analysis was also applied to the negative-sense virus RNAs and these “decumulated” values are plotted in the right-hand panels of Fig 4A. Due to the low production of mRNA4 relative to mRNA3, the amount of mRNA4 could not be estimated in this way. We also omitted the 1 h p.i. time point due to the low levels of virus reads (S1A Table). Translation efficiencies were calculated by dividing the raw RiboSeq densities for each ORF by the decumulated RNASeq densities for the corresponding mRNA. Note also that initiation and termination peaks were excluded from the RiboSeq density calculations (see Methods).

The 3′ ORFs 2, S, 5, E, M and N are all translated at comparable efficiencies (Fig 4B). The translation efficiency of E was at the lower end, presumably due to it not being the 5′ proximal ORF on its transcript (mRNA5) [34]. The translation efficiency of N was also at the lower end. The translation efficiency of ORF1a/1ab was, in comparison to the 3′ ORFs, very low. As mentioned above, this could be due to a proportion of gRNA being present in an untranslatable pool, perhaps as RTCs or RNPs destined for packaging, but may also indicate a real restraint on ORF1a/1ab translatablity (see below). The gRNA translation efficiency calculated in this way was low even at 2.5 h p.i. (repeat 2, ORF1a translational efficiency ~0.11). On the assumption that gRNA will not be directed to a packaging pathway at early time points, this suggests that incoming and early synthesis gRNA is largely involved in RNA synthesis, or is, indeed, inherently poorly translated. It should be noted that technically these calculations do not measure translational efficiency absolutely, as ribosome occupancy may also be affected by translational speed (though, when averaging over ORFs, this effect is thought to be generally quite slight; [8]). Further, as new transcripts enter the translation pool, there may not have been time to establish steady state ribosome loading on any particular transcript, while, at late time points, translational efficiencies may be below their optimal values due to saturation of the host cell protein synthesis machinery.

### Analysis of RNASeq sequences spanning TRS sites

**Fig 5:**
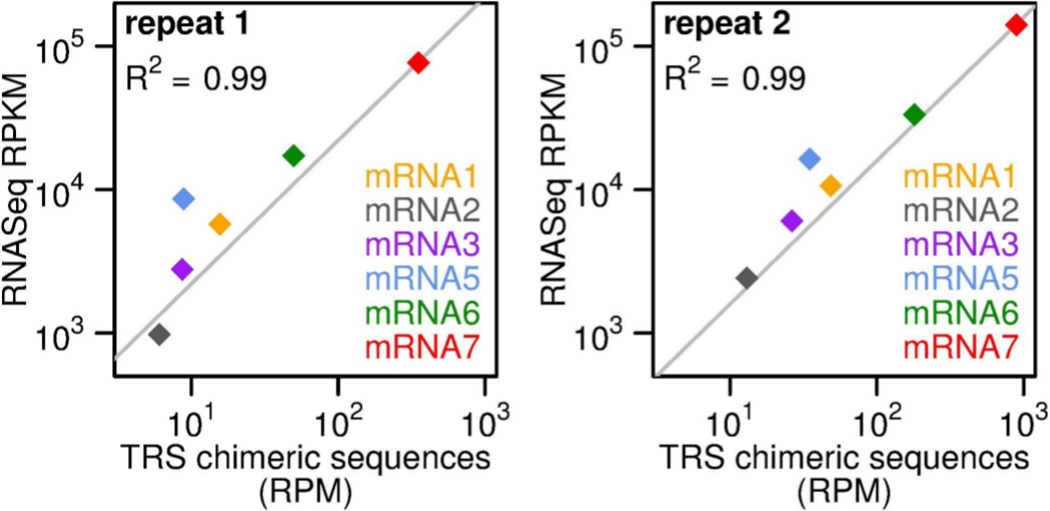
Comparison of estimators of relative mRNA abundance. Relative abundances of the different mRNA species (positive-sense) at 5 h p.i. were estimated either from mean RNASeq density (decumulated as described in the caption to Fig 4) or from the abundance of leader/body “chimeric” RNASeq reads spanning the corresponding TRS junction site. RNASeq densities are expressed in reads per kb per million mapped reads (RPKM). Chimeric TRS read counts are expressed in reads per million mapped reads (RPM).

Transcript abundances can be calculated from the decumulated RNASeq densities (as above) or, independently, from the relative abundances of RNASeq reads spanning each leader/body junction. Such “chimeric” reads (where the 5′ part maps to the leader sequence, and the 3′ part maps just downstream of a body TRS) were not included in the initial mapping to the virus genome (Fig 1B), but were identified subsequently (see Materials and Methods). Fig 5 compares mRNA abundances estimated using these two methods. The “TRS method” has the advantage that it avoids the potential inaccuracies introduced by decumulation but may be more subject to fragmentation, ligation and PCR biases due to the relatively short window in which to calculate a mean RNASeq density. Nonetheless there is a good correlation between the two estimates (R^2^ = 0.99).

In MHV, the consensus for canonical TRSs is UCUAAAC with minor exceptions being UCUAUAC for mRNA2 and UCCAAAC for mRNA6 [35–38]. A variable number of tandem copies (two in MHV-A59) of UCUAA are present at the leader junction site, while an imperfect copy of UCUAA precedes the canonical UCUAAAC at several body junction sites (S2 Table). Heterogeneity in the number of copies of the pentanucleotide has previously been observed to occur in mRNA6 for MHV-A59, and both mRNA6 and mRNA7 for MHV-JHM, and this is presumably due to heterogeneity in the site of re-annealing following a polymerase jump [39]. Consistent with this, we also observed significant usage of a junction site 5 nt upstream of the canonical site for mRNA6 (13–17% of mRNA6 transcripts) (S3 Table). We also observed this phenomenon for mRNA7 (0.5–0.8% of mRNA6 transcripts). The greater usage for mRNA6 is likely due to it having an imperfect pentanucleotide UCCAA at the canonical junction site but a perfect pentanucleotide UCUAA 5 nt upstream; in contrast, other mRNAs have a better pentanucleotide match at the canonical site than at the site 5 nt upstream (S2 Table). For mRNAs showing such heterogeneity, the summed values were used for Fig 5. For mRNA7, where the upstream pentanucleotide is CCUAA instead of UCUAA, we observed that the first nucleotide could be templated either by the body sequence (i.e. 'C'; ~40%) or by the leader sequence (i.e. 'U'; ~60%) (S3 Table, nt 29653 sequences).

We also observed many non-canonical leader/body chimeric sequences (S3 Table), though even the most frequent were present at ≤20% the level of leader/body chimeric reads for mRNA2 (the least abundant canonical mRNA). The coronavirus polymerase is known to engage in promiscuous jumping [39–41] and there is no reason to suppose that the additional transcript species generated this way are functionally relevant. Two of the most abundant (genomic coordinates 41 and 34 in S3 Table) involved apparent backward jumping by the polymerase (although inter-template jumping is another possibility). The sequences at non-canonical junctions often partly resembled canonical TRSs (e.g. UCUAAAa at nt 41, UCUcAAC at nt 34, cCUAcuu at nt 22483, UCcAAgc at nt 27106 and UgUAAua at nt 28847; canonical TRS nucleotides in upper case). In cases where the nucleotides at +1 to +2 in the genome sequence differed from UC, the RNASeq read generally contained nucleotides templated by the genome sequence rather than the UC in the leader sequence (e.g. CC instead of UC for the nt 22483 junction), although there were exceptions (e.g. UC instead of AA for the nt 22582 junction) (S3 Table). This latter site, AAUAAGC, aligns with a TRS previously identified for an HE mRNA in the JHM strain of MHV [38]. The sequence in MHV-JHM is AAUAAAC, differing from the MHV-A59 sequence by a G to A substitution. An HE mRNA has not been observed for MHV-A59 and this is likely due to the greater deviation from the consensus TRS, UCUAAAC, in this strain [38, 42]. Although we observe some usage of this site in our sequencing, the levels are extremely low—just 3.6–4.6% those of mRNA2 (the least abundant canonical mRNA).

### Comparison of host and virus translation efficiencies

**Fig 6:**
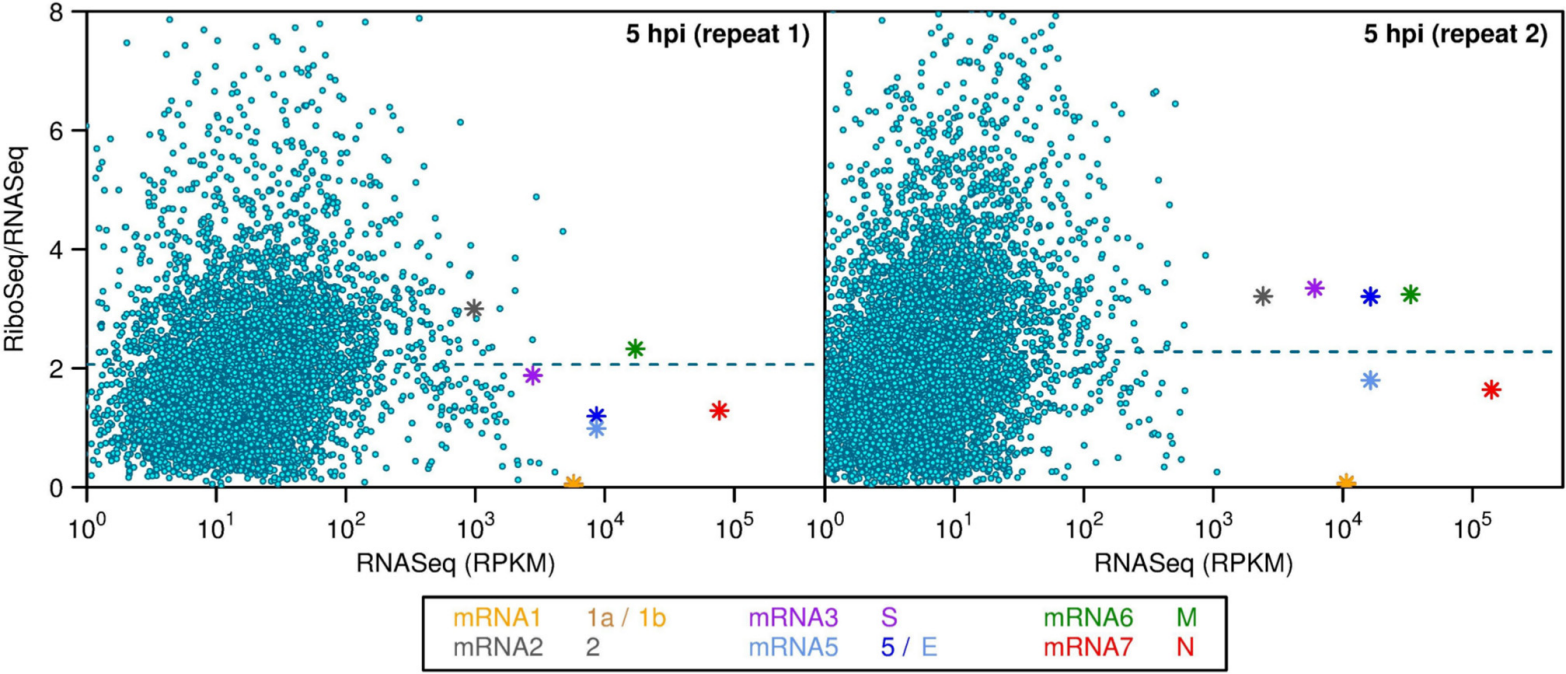
Comparison of host and virus translation efficiencies. The translation efficiencies of virus mRNAs were calculated as described in the caption to Fig 4. Host mRNA translation efficiencies are based on the ratio (after normalization for library size) of all RiboSeq or RNASeq reads mapping to any annotated coding region of any splice form of a given gene (see Methods). Host data are shown only for genes with >100 mapped RNASeq coding-region reads (prior to normalization for library size). Horizontal dashed lines indicate the mean values for host cell genes.

Fig 6 compares the translation efficiencies at 5 h p.i. of virus and host CDSs. The former are as described previously in Fig 4B. The latter are calculated on a per gene (rather than per transcript) basis, using RNASeq and RiboSeq reads contained entirely within annotated CDS regions (i.e. excluding 5′ and 3′ UTRs and also RPFs accumulating at or near to initiation or termination sites), and, like the virus values, are expressed relative to the mean levels for the cell (due to normalization by library size). The analysis shows that the virus translation efficiencies fall within the general range of those of host genes (except for ORF1a/1b which have particularly low translation efficiencies; see above) indicating that virus transcripts are not preferentially translated during virus infection. Instead, massive production of virus proteins (in particular the N protein) is achieved through high levels of transcription.

### Virus RNA and protein synthesis in the initial stages of infection

**Fig 7:**
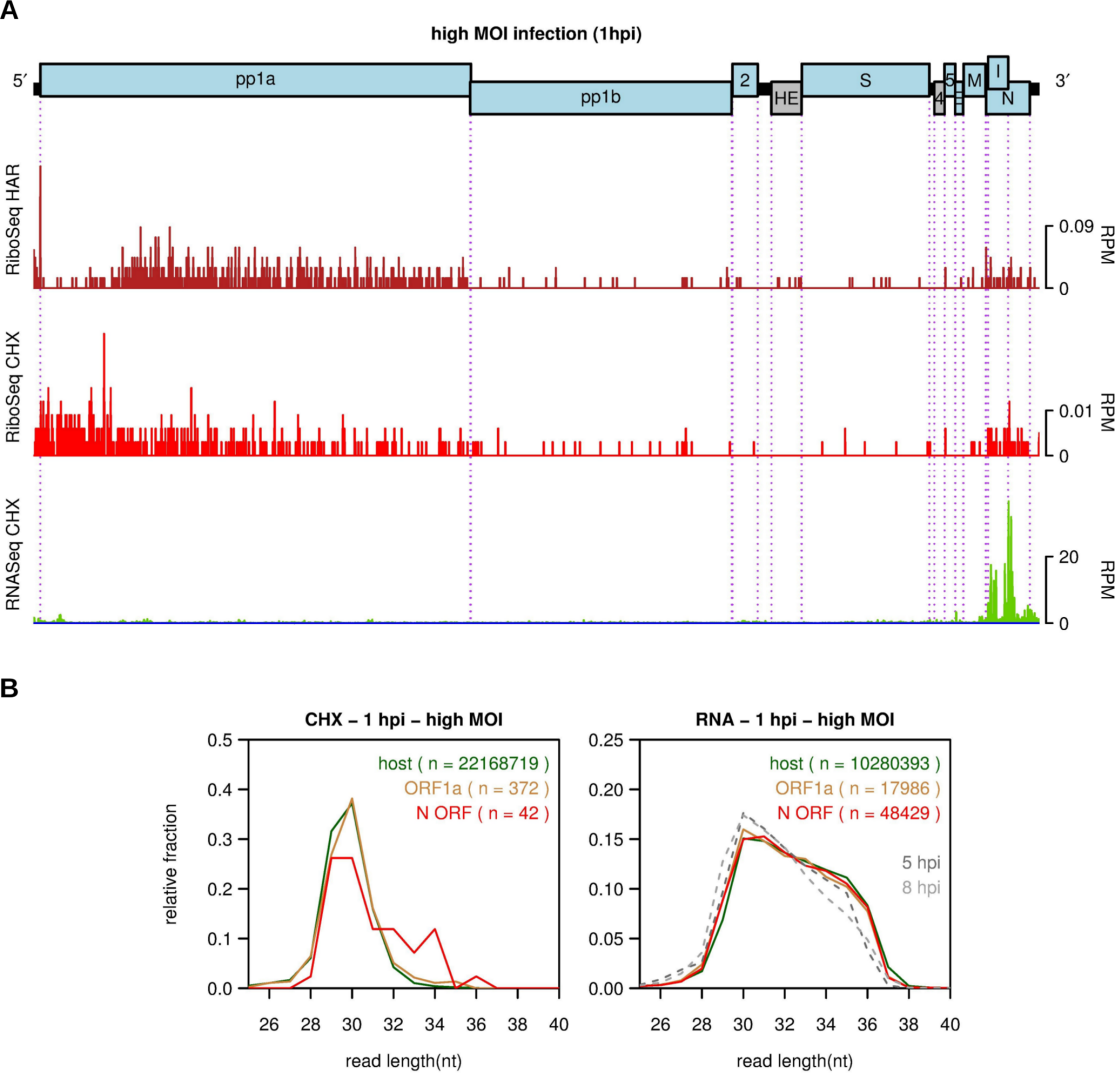
MHV RNA synthesis and translation at an early time point. **(A)** RiboSeq HAR (dark red), RiboSeq CHX (red) and RNASeq (green) density at 1 h p.i. in reads per million mapped reads (RPM), smoothed with a 15-nt running mean filter and plotted on a linear scale. To obtain sufficient reads at an early time point, cells were infected at an MOI of 200. Histograms show the positions of the 5′ ends of reads with a +12 nt offset to map (for RPFs) approximate P-site positions. **(B)** Comparison of the read length distributions of 5′ (ORF1a; orange) and 3′ (N ORF; red) virus reads with host mRNA reads (green) from the same samples. In the RNASeq graph (right), read length distributions are also shown for virus RNA from the 5 h p.i. and 8 h p.i. repeat 2 samples (grey).

To study virus RNA synthesis and translation during the earliest stages of infection, we did high MOI (~200) infections to maximize the number of virus reads in the libraries. The composition of the high MOI libraries is summarized in S1B Table and S12A Fig. Fig 7A shows the distributions of RiboSeq and RNASeq reads on the virus genome at 1 h p.i. (where 0 h p.i., is the time at which the inoculating virus is removed). A 5′ to 3′ decreasing gradient in RPF density is visible within ORF1a in the RiboSeq CHX density profile, while very few RPFs were found within ORF1b, indicating that, at 1 h p.i., ribosomes have only had time to translate part of the 4470-codon ORF1a. This does not indicate the translate rate *per se*, as time is also required for uncoating, recruitment of ribosomes, translation of a uORF on the gRNA (see below), and potential delays with initiation and reinitiation (see also below). In the RiboSeq HAR data, a clear trough in RPF density is visible after the ORF1a initiation peak, followed by higher density further downstream in ORF1a. The trough reflects run-off of elongating ribosomes in the three minutes between addition of HAR (which inhibits new initiation events) and CHX (which freezes the ribosomes). Taking the width of the trough as ~750 codons, this gives an elongation rate of 4.2 amino acids s^−1^, similar to that determined previously in mouse embryonic stem cells (5.6 amino acids s^−1^) [8].

Despite the very high MOI, virus RNA levels were low except, unexpectedly, in the N region where the mean RNASeq density was ~27 times that in the ORF1a region. To test whether this might be due to contamination from late time-point samples, we compared the length distribution of reads in the N region with the length distribution of reads mapping to host mRNAs for the same sample (Fig 7B, right panel; red and green lines, respectively). The two distributions were very similar. In contrast, the length distributions of virus-derived reads from the 5 and 8 h p.i. RNASeq time points (from repeat 2 which was co-processed with the high MOI libraries) were different in shape (Fig 7B, right panel; grey lines). While it is impossible to definitively rule out contamination in this way, the analysis suggests that the RNASeq density in the N region at 1 h p.i. is not a result of contamination. Since, for mRNA7, negative-sense RNA is present at >0.1% of positive-sense RNA at 2.5, 5 and 8 h p.i. (Fig 4), the absence of appreciable levels of negative-sense reads mapping to the N region in the high MOI 1 h p.i. sample (3 negative-sense compared with 48,429 positive-sense reads; 0.006%) also argues strongly against the positive-sense reads being inter-library contaminants. The near-complete absence of negative-sense reads also argues against this phenomenon being a result of early synthesis. Moreover, the absence of equivalent RNASeq density in the leader region (cf. Fig 2) argues against the density in the N region deriving from *bona fide* mRNA7 transcripts. Closer inspection revealed a number of a relatively abundant chimeric reads suggesting a mosaic structure of rearranged N-ORF sequences, reminiscent of defective interfering (DI) RNAs [43, 44]. However, since coronavirus DI RNAs are expected to include parts of the 5′ end of the genome and a packaging signal, and only arise after multiple high-MOI passages, we believe the N ORF transcripts we have identified must represent a novel class of packaged transcripts. An alternative, albeit unlikely, explanation is that the excess 3′ density may be a result of selective degradation (either natural or artifactual) of ~96% of the input gRNA.

Relative to RNA levels, very few RPFs mapped to the N ORF region and we were unable to ascertain whether or not they resulted from contamination from other samples as, in contrast to RPFs from ORF1a, their length distribution only partly matched the length distribution of host RPFs (Fig 7B, left panel, red line). Using these RPF counts, the N ORF translation efficiency (normalized to total virus RNA and host mRNA) was calculated to be only 0.0005, compared to values in the range 1.1 to 1.7 at the 2.5, 5 and 8 h p.i. timepoints, indicating that the early timepoint N ORF RNA revealed by RNASeq is not, or only barely, translated.

### High efficiency of −1 programmed ribosomal frameshifting in MHV

**Fig 8:**
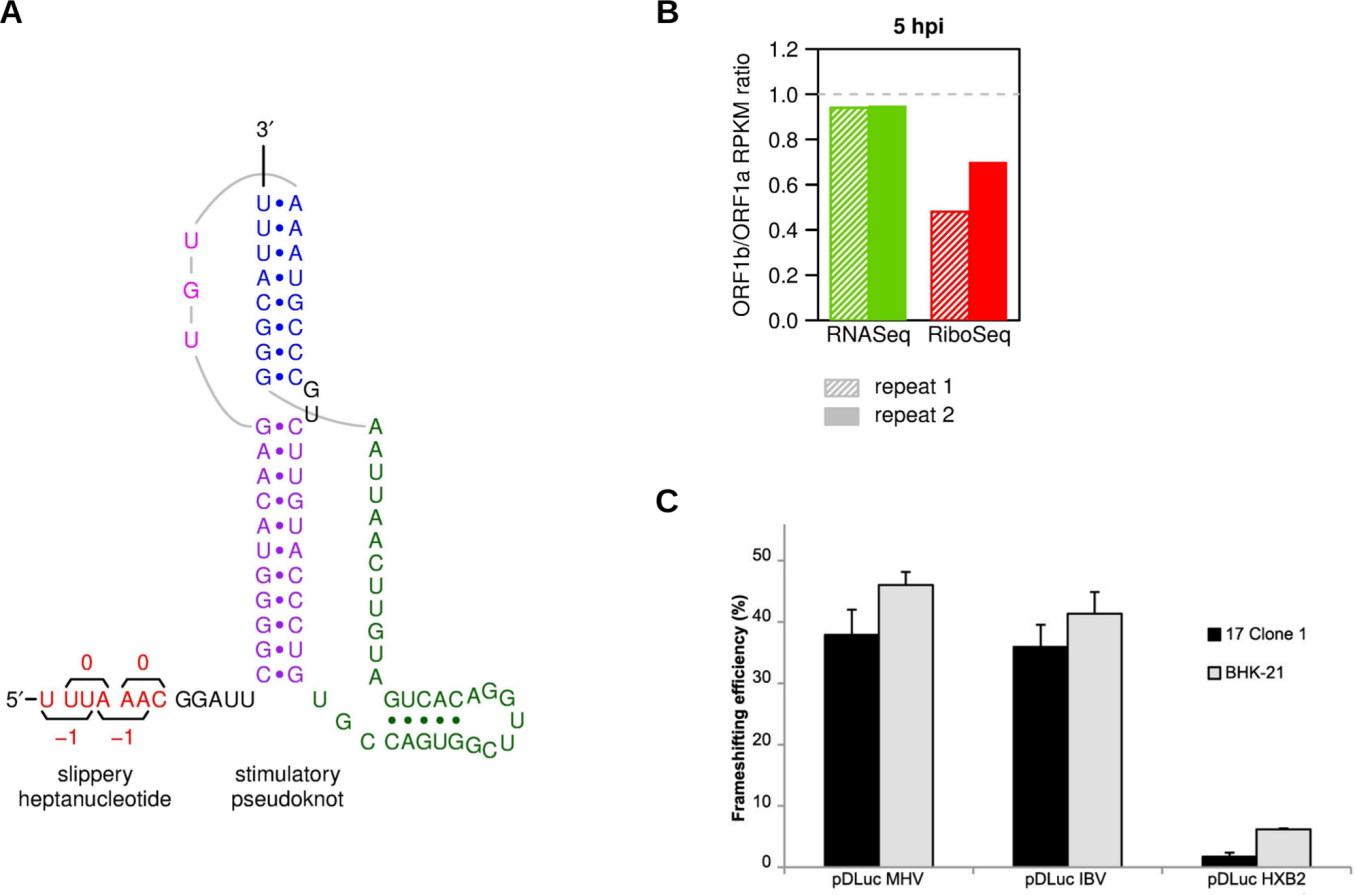
Frameshifting efficiency. **(A)** Schematic of the MHV frameshifting signal comprising a slippery heptanucleotide, U_UUA_AAC, and downstream pseudoknot stimulatory structure. **(B)** Frameshifting efficiencies estimated from the ratio of RiboSeq density in ORF1b to that in ORF1a (red). For comparison, the same calculation was done for RNASeq (green). ORF1a and ORF1b are both present only on the genomic RNA so the ratio of RNASeq densities in the two ORFs is expected to approximate unity. **(C)** Frameshifting efficiencies for MHV, IBV and HIV-1 frameshift cassettes determined using dual luciferase assays in 17 Cl-1 and BHK-21 cells. Cells were transfected with pDLuc-MHV, pDLuc-IBV or pDLuc-HXB2, and 24 h later, lysates were prepared and assayed for *Renilla* and firefly luciferase activity.

The −1 PRF signal that facilitates expression of MHV pp1ab comprises two elements, a heptanucleotide slippery sequence (U_UUA_AAC), identical in all known coronaviruses, and an RNA pseudoknot structure located a few nucleotides downstream [5, 45, 46] (Fig 8A). During translation of the gRNA, elongating ribosomes either terminate at the ORF1a stop codon, yielding pp1a, or frameshift on the slippery sequence to translate ORF1b, yielding pp1ab. Frameshifting likely provides a fixed ratio of translation products for assembly into a macromolecular complex [47, 48]. Studies of frameshifting using reporter constructs expressed in transfected cells or through *in vitro* translation of synthetic mRNAs have indicated that the efficiency of the process in coronaviruses is generally in the region of 20–45% [4, 5, 49–51] However, the actual efficiency in the context of virus infection has not been previously determined.

Simplistically, one can calculate this value by dividing the RiboSeq density in ORF1b by the density in ORF1a. However, in principle, RiboSeq density represents the quotient of expression level and translational speed so the above calculation assumes that, on average, translation speed is the same in ORFs 1a and 1b and that translation is steady state. Such a calculation is, therefore, invalid immediately after infection (as ribosomes begin to translate ORF1a of the input gRNA but have not yet reached ORF1b; Fig 7) and may also be compromised if newly synthesised gRNA entering the translation pool represents a significant fraction of the gRNA undergoing translation. Thus, we measured the frameshifting efficiency at 5 h p.i., calculating values of 48% for repeat 1, and 70% for repeat 2 (Fig 8B). The former value (48%) is similar to previous *in vitro* measurements of the MHV frameshifting efficiency (40%) [5]. As the infection appeared to be more advanced in repeat 2 (Fig 3), it is possible that the higher level measured (70%) is a consequence of depletion of the host cell protein synthesis resources, e.g. exhaustion of initiation factors (including free ribosomes) could decrease the density of ribosomes in ORF1a as elongating ribosomes run off, and a partial exhaustion of elongation factors could slow the establishment of a new steady state.

We also measured the frameshifting efficiency by means of transfected reporter constructs. We began by cloning a 100-bp fragment including the MHV frameshift signal (Fig 8A) into a dual luciferase frameshift reporter plasmid (pDluc; [52, 53]) between the *Renilla* (Rluc) and firefly (Fluc) luciferase genes. In this plasmid, frameshifting permits expression of Fluc as a fusion with Rluc (analogous to the expression of MHV pp1ab), while failure to frameshift results in expression of Rluc alone. Frameshifting efficiencies were calculated from the ratio of Fluc activity to Rluc activity, normalized by a control construct in which an extra C residue was inserted immediately downstream of the slippery sequence to place Rluc and Fluc in the same reading frame. The well-studied coronavirus frameshifting signal from *Avian coronavirus*, infectious bronchitis virus (IBV) served as a positive control, alongside a lower efficiency control (the *gag*/*pol* −1 PRF signal of HIV isolate HXB2) [54, 55]. The MHV frameshifting efficiency was found to be 38% in 17Cl-1 and 45% in BHK-21 cells, and similar in both instances to that of IBV (Fig 8C). These data suggest that frameshifting in coronaviruses is not specifically modulated by virus infection, with the difference seen in the more advanced infection of repeat 2 likely due to the non-specific effects mentioned above.

### Ribosomes do not pause appreciably at the frameshift site

The relevance of ribosomal pausing to the mechanism of −1 PRF has long been a subject of debate [56–58]. Frameshift signal-associated pauses have been documented in a number of *in vitro* assays [59–64], but there is, as yet, little evidence for a causal relationship, and pausing has not been examined in infected cells. We therefore looked to see whether there was an accumulation of RPFs at the MHV frameshift site. In the initial RiboSeq time courses we failed to see significant pausing at the frameshift site. However, reasoning that the frameshift-stimulatory pseudoknot beginning 6 nt 3′ of the slippery heptanucleotide U_UUA_AAC would be partly inside the mRNA entrance channel at the onset of frameshifting, and might, due to its compact structure be somewhat resistant to RNase 1 digestion, we considered the possibility that frameshift-associated pauses may generate longer RPFs, which would be excluded from the profiling analysis as a result of gel size selection (28–34 nt). Thus we prepared new libraries from the 5 h p.i. repeat 2 RiboSeq CHX samples (see S1C Table for composition) using a larger gel slice (nominally 28–80 nt). However, even in these samples we failed to see noticeable pausing at the frameshift site (S11 Fig).

### Sites of ribosomal pausing in ORF1a

**Fig 9:**
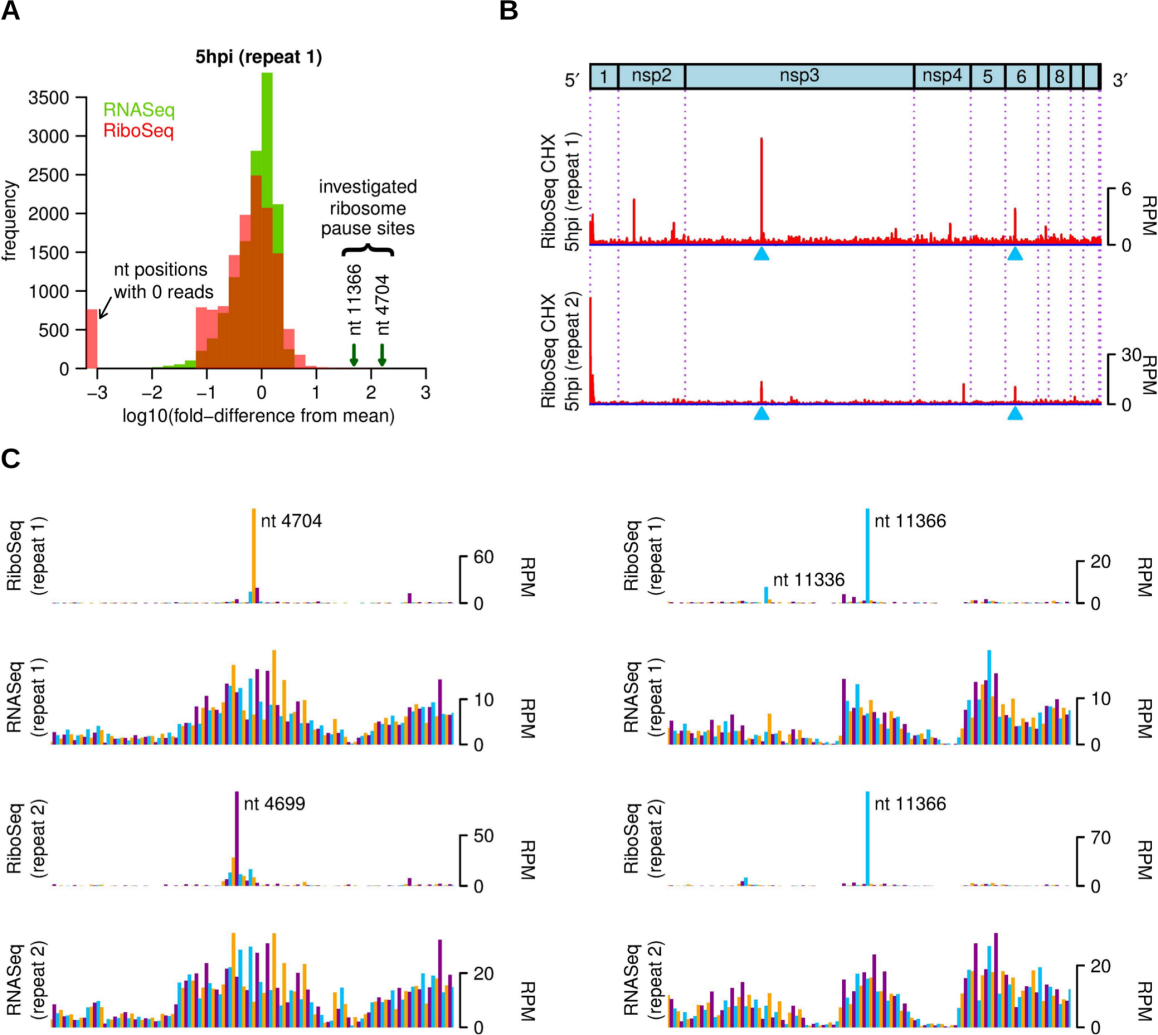
Ribosome pause sites in ORF1a. **(A)** Histograms of log fold-change from the mean in ORF1a (5 h p.i., repeat 1) showing that RiboSeq densities are more variable than RNASeq densities. RNASeq and RiboSeq counts in ORF1a were first smoothed with a 3-nt running mean filter to average out the intra-codon variability (i.e. triplet periodicity) present in RiboSeq data. **(B)** Blue triangles indicate selected sites of RPF accumulation in ORF1a, indicative of ribosomal pausing (see text). Histograms show the positions of the 5′ ends of reads with a +12 nt offset to map the approximate P-site. RPF distributions were smoothed with a 15-nt running-mean filter (which, incidentally, reduces the peak height ~15-fold, cf. part C). **(C)** Enlarged view of the two pause sites without smoothing. The 3′ pause corresponds to reads with 5′ ends mapping to genomic coordinate 11366 while the positions of the 5′ ends of reads at the 5′ pause site differ by 5 nt between the two repeats (genomic coordinate 4704 and 4699, respectively). Reads whose 5′ ends map to the first, second or third positions of codons are indicated in purple, blue or orange, respectively.

Although we failed to identify significant pausing at the frameshift site, there were other sites at which RPFs accumulated to a much higher level than at neighbouring sites. We frequently observed such accumulations at initiation sites (possibly an artifact of CHX treatment; [8]) (Fig 1 and Fig 2), but also at internal sites within ORFs. Besides ribosome pausing, fluctuations in RPF density may occur as a result of nuclease, ligation, and PCR biases. The latter two occur also for RNASeq, whilst in RNASeq nuclease bias is replaced by fragmentation bias. Following [65], we compared the distributions of variability in RiboSeq and RNASeq densities within ORF1a, which revealed that RiboSeq densities were more variable than RNASeq densities (Fig 9A), with the extra variability presumed to be a result of fluctuations in ribosome progressivity. We focused on two of the highest RiboSeq density peaks in the ORF1a region (blue arrows in Fig 9B).

RPFs at the second of the two pause sites, located in the nsp6 region, have 5′ ends that map almost exclusively to nt 11366 which, unusually, corresponds to the second codon position (Fig 9C, right). The 3′ end positions of these RPFs were, as is normal, more variable, with the most abundant 3′ ends mapping to nt 11393–11394 for repeat 1 and nt 11394–11395 for repeat 2, giving read lengths of 28–29 and 29–30 nt which are within the typical range for the respective samples (S2 Fig and S3 Fig). For these samples, RiboSeq CHX 5 h p.i. repeats 1 and 2, 64% and 66%, respectively, of host mRNA RPFs have 5′ ends mapping to codon position 1, with only 7% and 8% mapping to codon position 2. The reason for the deviation at the pause site is unknown but may be a result of “tension” within the mRNA or perturbation of the ribosome conformation [66]. Due to the unusual codon position of the 5′ end, it was not possible to definitively predict the P-site position of ribosomes at this pause site, but it is more likely to be at nt 11377 to 11379 (AAA codon) than at nt 11380 to 11382 (CAG codon) as the lengths of the most abundant reads (28–29 and 29–30 nt in repeats 1 and 2, respectively) are more consistent with reads being 1 nt shorter than normal at the 5′ end, rather than 2 nt longer. The nascent peptide sequence (i.e. peptide sequence within the ribosome exit tunnel that could potentially affect pausing) here is…IKHKHLYLTMYIMPVLCTLFYTNYLVVYKQ (P-site amino acid underlined). An additional smaller peak was apparent 30 nt upstream (in repeat 1) and potentially corresponds to a following ribosome stacking behind a proportion of paused ribosomes.

RPFs at the first of the two pause sites, located in the nsp3 region, have 5′ ends that map to nt 4704 in repeat 1 but nt 4699 in repeat 2 (Fig 9C, left). However, this 5 nt difference is made up in the length of the reads (top three read lengths 28, 27 and 29 nt in repeat 1, but 33, 34 and 32 nt in repeat 2) so that the 3′ ends of the RPFs map to similar positions in both repeats. Incidentally, this difference in 5′ end position between the two repeats makes it highly unlikely that the peak is an artefact of ligation bias. In general the nuclease trimming seems to be less stringent in repeat 2 than in repeat 1 (host mRNA RPF lengths peak at 29–30 nt in repeat 1 but at 30 nt in repeat 2; S2 Fig and S3 Fig). The pause site read length in repeat 2 is unusually long, indicating that the extra ~5 nt at the 5′ end are, for some reason, partially protected, resisting trimming in repeat 2 but not in the more stringently trimmed repeat 1. The nature of this protection (scrunching of extra mRNA into the mRNA exit channel, formation of an RNase-resistant RNA structure 5′-adjacent to the ribosome, conformational changes in the ribosome, or an additional ribosome/mRNA-associated protein factor) and whether and how it is linked to pausing remain undetermined. The nascent peptide sequence is…EKCQVTSVAGTKALSLQLAKNLCRDVKFVT (P-site amino acid underlined).

The nascent peptides at both pause sites lack the E- or P-site prolines or A-site GAA codons that are commonly associated with pausing in ribosome profiling meta-analyses [8], though many diverse nascent peptides are also known to perturb ribosome progressivity [67, 68]. As an alternative possibility, we analysed the RNA downstream of the pause sites for evidence of stable RNA structures that could induce pausing, but nothing was apparent. An alternative explanation is that these pauses are induced by *trans*-acting factors, e.g. RNA binding proteins, or chaperones of the nascent peptide.

### Experimental analysis of the nsp3 pause site

**Fig 10:**
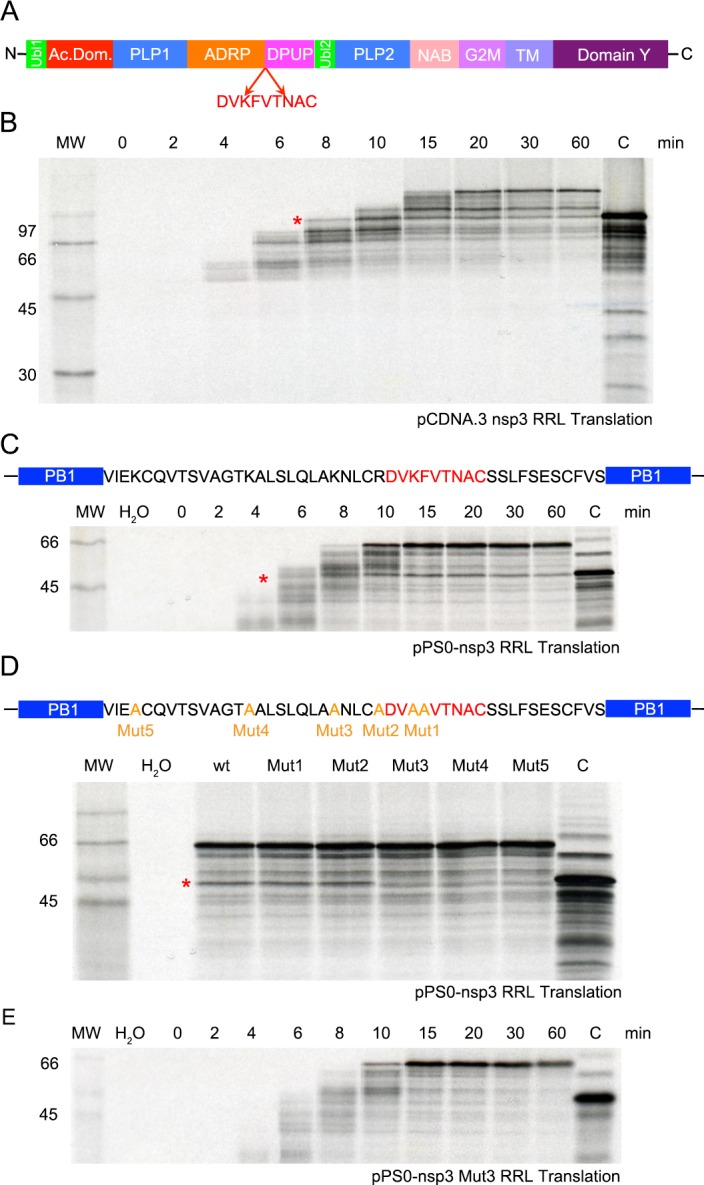
Determination of MHV-nsp3 pausing site. (**A**) Nsp3 is the largest protein encoded in the MHV replicase gene and contains two ubiquitin-related domains (green), a hypervariable acidic domain (red), two papain-like cysteine proteinase domains (PLP1 and PLP2; blue), a poly (ADP-ribose) binding activity (ADRP) domain (orange), the recently described “domain preceding Ubl2 and PLP2” (DPUP; fuchsia) [76], the nucleic-acid binding domain (NAB; salmon) the betacoronavirus marker (G2M; lavender), a transmembrane domain (TM; orchid) and domain Y (plum). The site of ribosomal pausing, DVKFVTNAC (P-site at pause underlined) is indicated. (**B**) Time course of translation of pcDNA.3 mRNA, containing sequence coding for nsp3* (first 1,125 residues excluding NAB, G2M, TM and Y domains) in RRL. Translation was allowed to proceed at 26°C in the presence of [^35^S]methionine for 3 min prior to addition of edeine to a final concentration of 5 μM. Samples were withdrawn at the indicated times after edeine addition, and translation products separated on a 10% SDS-PAGE gel and detected by autoradiography. MW indicates ^14^C-labelled molecular weight standards and H_2_O as a negative control. The predicted position of the pause product was determined from the “pause control” lane (see text). (**C**) Time course of translation of pPS0/nsp3-derived mRNA in RRL as above. pPS0 contains, under the control of the SP6 promoter, a copy of the influenza virus PB1 gene into which has been inserted cDNA encoding the nsp3 pause region (red) plus 30 upstream residues. (**D**) Ribosomal pausing assays of pPS0-nsp3 mutant mRNAs in RRL (20 min at 26°C). In each case, positively charged or aromatic amino acids were changed to alanine. In mutant 1, Lys-Phe at the pausing site was changed to Ala-Ala, and subsequent mutants were prepared sequentially from this clone, thus mutant 5 contains six substitutions (see text). (**E**) Ribosomal pausing assay of pPS0/nsp3 Mut3 mRNA in RRL as described above. In all panels, the pause product is indicated by a red asterisk.

Coronaviruses induce substantial membrane rearrangements in the infected cell, including formation of a reticulovesicular network composed of two types of membrane modifications, double-membrane vesicles (DMVs) and convoluted membranes (CM). The reticulovesicular network is contiguous with the endoplasmic reticulum (ER) and is the site of virus RNA synthesis [69, 70]. Nsp3, nsp4 and nsp6 are integral membrane proteins whose topology has been determined *in vitro* for SARS-CoV and MHV [71–74], and, in the case of SARS-CoV, have been shown to be necessary and sufficient for double-membrane vesicle formation [75]. Nsp3 is the largest protein encoded in MHV ORF1a and contains multiple domains, including two small ubiquitin-like domains (Ubl1 and Ubl2), two papain-like cysteine proteinase domains (PLP1 and PLP2), a poly-ADP-ribose-binding activity (ADRP domain), the newly determined domain preceding Ubl2 and PLP2 (DPUP; [76]), the nucleic-acid binding domain (NAB), the betacoronavirus marker (G2M), a transmembrane domain (TM) and domain Y (Fig 10A). The apparent ribosome pause occurs within the sequence DVKFVTNAC (P-site at pause underlined; Fig 10A) which is located between the ADRP and the DPUP domains.

We investigated the nsp3 pause *in vitro* in rabbit reticulocyte lysate (RRL) translations using edeine assays [60]. Initially, a cDNA fragment comprising the first 1,125 residues of nsp3 (nsp3*; excluding the NAB, G2M, TM and Y domains) was cloned into pcDNA3.1 and synthetic mRNAs translated in RRL for 3 min prior to addition of the translation initiation inhibitor edeine. Incubation was continued, samples withdrawn at the indicated times post-edeine addition and translation products separated on a 10% SDS-PAGE gel (Fig 10B). To accurately mark the position of the predicted pause product, a control mRNA in which a UAA stop codon had been introduced at the pausing A-site was also translated. As seen in Fig 10B (marked by a red asterisk) a distinct translational pause was observed during translation of nsp3*, migrating at the same position as the “pause control” and accumulating and then diminishing as translation proceeded. To more closely define the stalling sequence, a region encoding 46 amino acids of nsp3 including the putative pausing peptide was cloned into the influenza PB1 reporter gene in transcription vector pPS0 (Fig 10C) [60] and edeine assays performed as above. Once again, a clear ribosomal pause was evident (Fig 10C, red asterisk). The nsp3 sequence within pPS0 includes five upstream positively charged amino acids (four Lys and one Arg) and one aromatic residue (Phe) that could potentially contribute to pausing [77]. These residues were mutated to alanine sequentially and incrementally (pPS0-nsp3 mutants), such that in Mut 1, Lys-Phe adjacent to the pausing site was changed to Ala-Ala, Mut 2 had these changes plus Arg to Ala, and so on, as shown in Fig 10D. Edeine assays were performed and a single time point (20 min) from each mutant analysed by SDS-PAGE. As seen in Fig 10D, pausing was obviated in Mut 3, Mut 4 and Mut 5, indicating that the residue substituted by alanine Mut 3 is likely to be a major contributor to the ribosomal pause. A complete time-course of pPS0-nsp3 Mut3 confirmed the lack of pausing (Fig 10E).

### Translation in the leader region

**Fig 11:**
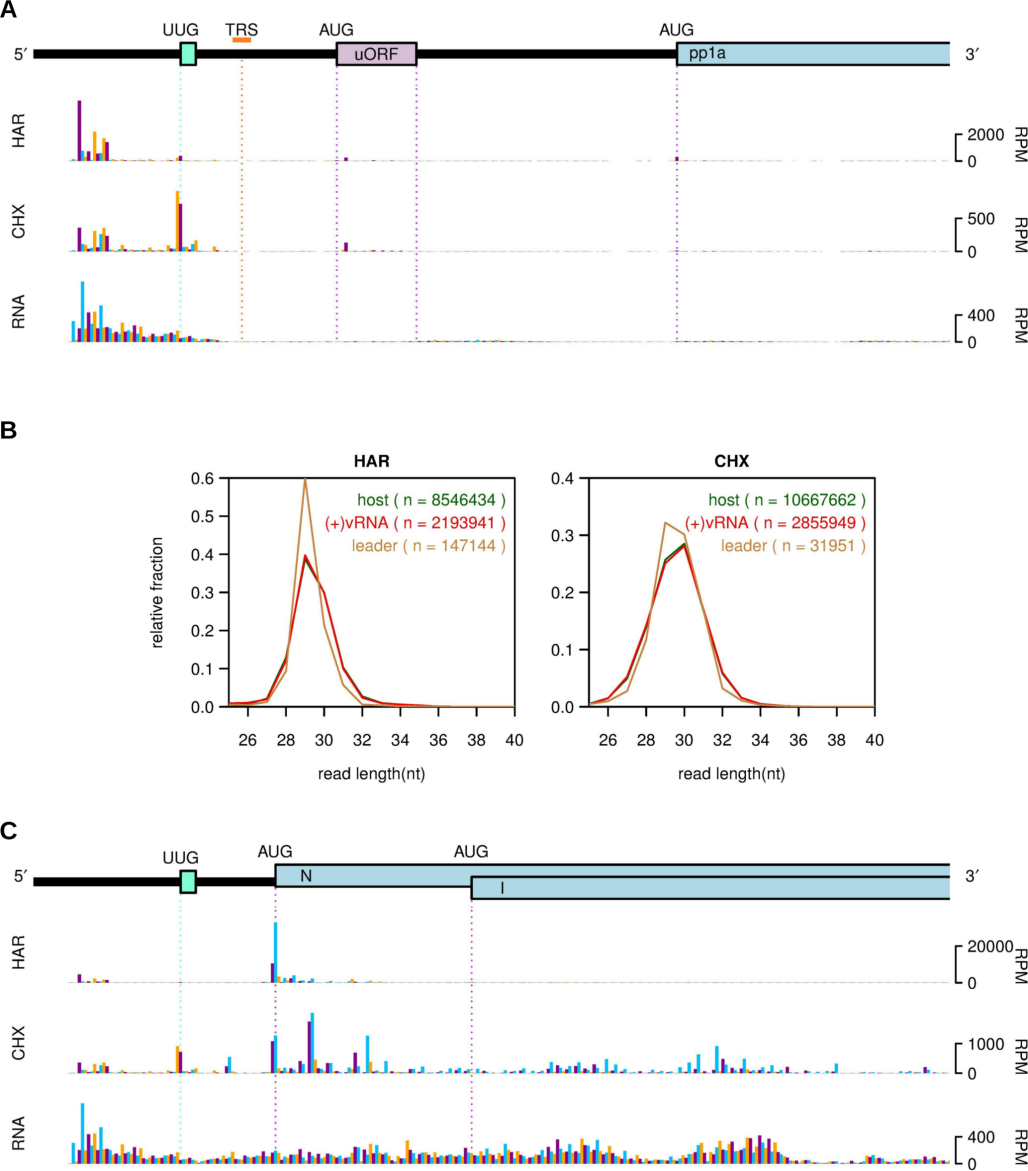
RiboSeq and RNASeq densities in the leader region and 5′ end of the genomic and N mRNAs. **(A)** A map of the 5′ end of the genomic RNA is shown at the top indicating a 1-codon non-AUG potential uORF (turquoise; sequence UUG UAG) in the leader, the leader TRS (orange), an 8-codon AUG-initiated uORF (lilac) present only in the genomic RNA, and the 5′ end of ORF1a (light blue). RiboSeq (CHX and HAR) and RNASeq (RNA) counts are shown for repeat 1 at 5 h p.i. Histograms show the positions of the 5′ ends of reads with a +12 nt offset to map the approximate P-site (the +12 nt offset means that genome coordinates 1 to 12 register zero counts). Reads whose 5′ ends map to the first, second or third positions of codons relative to the reading frames of the two uORFs and ORF1a (which are all in phase) are indicated in purple, blue or orange, respectively. **(B)** Comparison of the read length distributions of 5′-end-of-leader RPFs (5′ end of RPF maps at or 5′ of genome coordinate 32) (orange), all virus RPFs (red), and host mRNA RPFs (green) from the same samples. **(C)** Mapping of reads specifically to mRNA7. After subtracting rRNA (see Methods) reads were mapped to mRNA7 instead of the MHV genome. The 5′ region of mRNA7 are shown.

Fig 11A displays RiboSeq and RNASeq densities for the 5′ region of the genome at 5 h p.i. The leader sequence, 5′ of the leader TRS (orange, Fig 11A), is present on all mRNAs so reads mapping to this region may derive from any mRNA, although most are expected to derive from the highly abundant mRNA7. The plot excludes “chimeric” reads (i.e. reads that span a TRS transcriptional discontinuity), so the RNASeq density drops close to the TRS site and the same is also expected to happen for RiboSeq. Probing of initiation sites through harringtonine treatment revealed unexpectedly that a substantial number of reads accumulate at or near the 5′ end of the leader, despite an absence of AUG codons. These 5′-proximal reads have a tight length distribution characteristic of true RPFs (Fig 11B; left panel) so are likely to be *bona fide* RPFs rather than some form of contamination. The 5′ portion of the leader contains a number of potential near-cognate non-AUG initiation codons, but most of the harringtonine reads do not obviously map to these. For example, the most abundant RPF position corresponds to a GCG codon (genome coordinates 16–18); initiation at this point would generate a 12 amino acid peptide, but it should be noted that GCG is not a recognised non-AUG initiation codon.

Elongation profiling with cycloheximide revealed a similar pattern of reads in the 5′ part of the leader but also a larger peak on a UUG codon close to the 3′ end of the leader sequence (Fig 11A). UUG is a known, albeit quite inefficient non-AUG initiation codon [8, 78] and, in this case, it is also in a poor initiation context (cucUUGuag; in mammals contexts with an A at −3, or a G at −3 and a G at +4, may be regarded as “strong”; [79]), so only a very small proportion of ribosomes would be expected to initiate here. Consistent with this, the HAR peak is very small compared to that seen at the N initiation codon (1.4%; Fig 11C) (though similar in magnitude to initiation peaks at the uORF and ORF1a on the genomic RNA; Fig 11A). Interestingly, the difference between the UUG peak and the N initiation peak was much less for the CHX samples (69%; Fig 11C). The reasons for this are unknown, but may be related to the UUG codon being immediately followed by a termination codon, with the peak potentially being derived from both initiation and termination pauses (UUG in P-site, UAG in A-site). We note also that, on mRNA7, the UUG codon is 31 nt upstream of the N initiation codon, so that initiation at N might lead to stacking of ribosomes on the UUG codon, potentially increasing initiation on this ostensibly very weak start codon.

### Translation upstream of ORF1a

**Fig 12:**
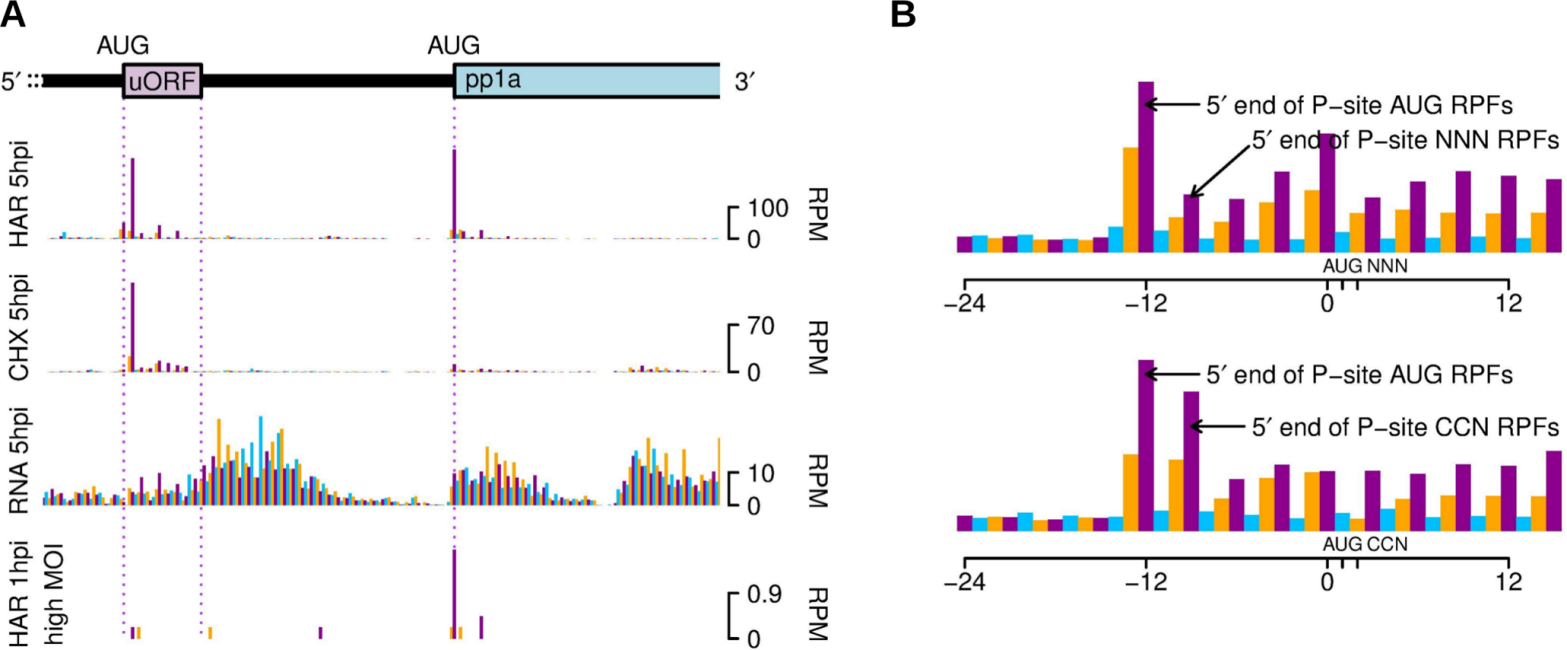
Translation of the genomic RNA uORF. **(A)** RiboSeq (CHX and HAR) and RNASeq (RNA) counts are shown for repeat 1 at 5 h p.i.; RiboSeq HAR counts are also shown for the high MOI infection. Histograms show the positions of the 5′ ends of reads with a +12 nt offset to map the approximate P-site. Reads whose 5′ ends map to the first, second or third positions of codons relative to the reading frames of the uORFs and ORF1a (which are in phase) are indicated in purple, blue or orange, respectively. Note that the illustrated region does not extend to the genomic 5′ terminus. **(B)** Comparison of RiboSeq CHX densities summed over all host NCBI RefSeq mRNAs and summed over mRNAs whose annotated coding sequences begin with AUG-CCN (Met-Pro). Histograms show the positions of the 5′ ends of reads, e.g. RPFs of ribosomes paused during initiation (AUG in the P-site at position 0 to 2) have 5′ ends that map predominantly to −12 or −13.

Downstream of the leader TRS but upstream of ORF1a, is a single, short AUG-initiated uORF that is present in many coronaviruses and believed to play a role as a regulator of genomic RNA translation, virus replication and pathogenesis [80]. Upstream ORFs are present in ~40% of mammalian mRNAs and have been shown generally to cause repression of translation of the downstream (main) ORF [81, 82]. We observed RPFs mapping specifically and in-frame to the uORF, confirming that it is translated. Indeed, at 5 h p.i. it appeared to be translated as efficiently as ORF1a (Fig 12A) despite its poor initiation context (uccAUGc; cf. auaAUGg for ORF1a) suggesting that it inhibits ribosomal access to ORF1a. This effect appeared less pronounced at early time points, suggesting a potential role for temporal regulation of replication protein synthesis (Fig 12A, bottom panel). Interestingly, we observed the greatest density of RPFs on the second codon (proline) rather than the first codon (methionine) of the uORF, both for HAR and CHX-treated samples. Prolines are often associated with ribosome pausing due to their restrained geometry in the decoding centre and/or ribosome exit tunnel [8, 83, 84]. To see if N-terminal Met-Pro was associated with ribosomal pausing on other mRNAs, we compared mean ribosome profiles for host mRNAs with CDSs beginning with AUG-CCN with mean ribosome profiles for generic host mRNAs and found that, particularly under conditions of virus infection, ribosomes tend to pause more at the second codon in the former (Fig 12B), although the ratio of ribosome occupancy between the AUG and CCN averaged over host mRNAs was less extreme than is the case for the virus uORF. It should be noted that, although presence of the uORF is conserved in 17 of 18 NCBI betacoronavirus RefSeqs, CCN occurs as the second codon in only six of these.

### Translation 5′ of other annotated ORFs

**Fig 13:**
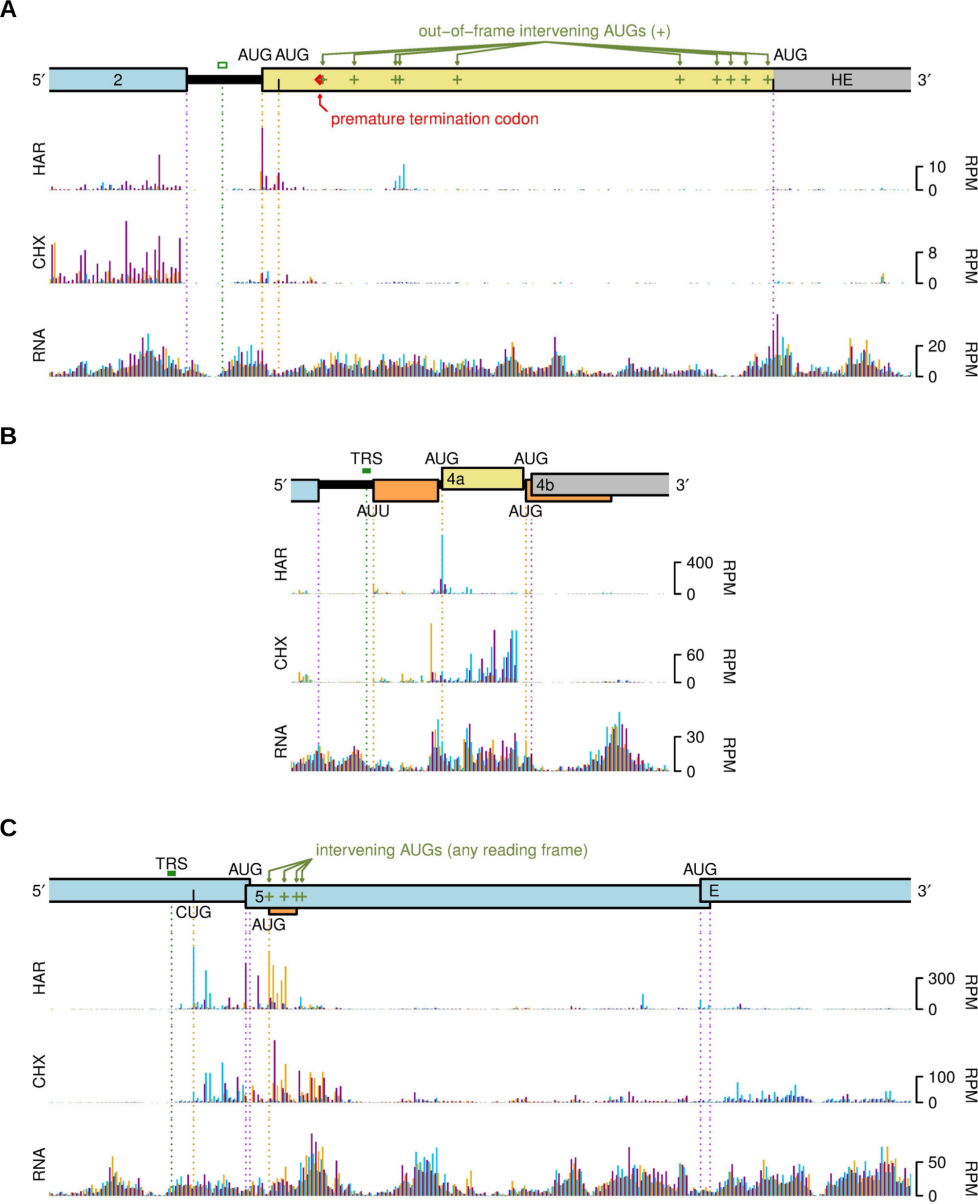
Analysis of translation upstream of other annotated ORFs. RiboSeq (CHX and HAR) and RNASeq (RNA) counts are shown for repeat 1 at 5 h p.i. Histograms show the positions of the 5′ ends of reads with a +12 nt offset to map the approximate P-site. Reads whose 5′ ends map to the first, second or third positions of codons relative to the reading frame of the main annotated ORF (i.e. HE, 4b or 5, respectively) are indicated in purple, blue or orange, respectively. **(A)** 5′ of the HE ORF. A defective TRS for a very low abundance HE mRNA is annotated with an open green box. In MHV-A59, the HE ORF is disrupted with a premature termination codon (red diamond). Out-of-frame AUG codons that would inhibit ribosomal access via leaky scanning to the next HE-frame AUG codon downstream of the premature termination codon are indicated in green. **(B)** 5′ of ORF4. In MHV-A59, ORF4 is split by a frameshift mutation into ORF 4b (grey) and a very short ORF4a (pale yellow). An upstream AUU-initiated short ORF and a short out-of-frame AUG-initiated ORF are shown in orange. **(C)** 5′ of ORF5. A CUG codon in the same frame as the upstream ORF4, and a short out-of-frame AUG-initiated ORF are indicated.

Fig 13 shows histograms of RiboSeq (CHX and HAR) and RNASeq reads that map near to the 5′ ends of the HE, 4 and 5 ORFs. Again, “chimeric” leader/body reads spanning transcriptional discontinuities at the TRS sites are excluded from these plots. In the laboratory-adapted strain MHV-A59, the HE ORF is disrupted by a premature termination codon (red diamond, Fig 13A) [85], and, furthermore, the TRS upstream of HE in MHV-A59 is defective (open green box, Fig 13A) [38], leading to only very low levels of HE mRNAs (see above). Although ribosomes were observed to initiate at the authentic HE AUG codon, upstream of the premature termination codon (Fig 13A, HAR), very little RiboSeq density was observed downstream of the premature termination codon. Translation of the annotated HE ORF (i.e. the long 3′ fragment; grey, Fig 1A and Fig 13A) was negligible, consistent with the presence of numerous AUG codons in other reading frames downstream of the “authentic” HE start codon, which would be expected to inhibit ribosomal access to the 3′ fragment of HE. The low level of initiation noted at the “authentic” HE start codon is likely explained by the very low levels of HE mRNA production inferred from the observation of a few RNASeq reads crossing the HE leader/body transcriptional discontinuity (see above and S3 Table), since leaky scanning on mRNA2 is unlikely to allow access to HE due to the large number of intervening AUG codons.

Similarly, in MHV-A59 the natural ORF4 coding sequence is split by a frameshift mutation into a short 5′ ORF4a (pale yellow, Fig 13B) and a longer 3′ ORF4b (grey, Fig 1A and Fig 13B) [86]. Again, we observed ribosomes initiating at the ORF4a AUG codon (Fig 13B, HAR, the blue peak is in the ORF4a frame), but very little RiboSeq density in the annotated ORF 4b. Ribosome access via leaky scanning to ORF4b would be inhibited not only by the ORF4a AUG but also by an additional out-of-frame AUG codon (Fig 13B). Upstream of ORF4a, but downstream of the mRNA TRS junction, a low level of initiation appeared to occur on an AUU codon (RiboSeq, HAR, orange peak). Ribosomes initiating here would translate a 15-codon ORF resulting in the peptide MYSILIATWPRKRQS (assuming the initiating codon AUU is decoded as Met). A similar ORF is present in other strains of MHV.

Upstream of ORF5, we identified an alternative initiation site at a CUG codon (Fig 13C, HAR, blue peak) which may have some bearing on the mechanism of expression of the E ORF, which lies downstream of ORF5 on the bicistronic mRNA5 (Fig 13C). The CUG codon in question is in the same reading frame as the upstream ORF4 and initiation here would result in translation of the last 13 codons of ORF4 with peptide sequence MVVHILLRHCPGI (assuming the initiating codon CUG is decoded as Met). The CUG is downstream of the mRNA5 TRS and appears to be utilized only on this mRNA as the RiboSeq density on the upstream part of the defective ORF4 (see above) is negligible. The level of initiation at the CUG was comparable to that at the ORF5 AUG (Fig 13C) and translation of this short ORF might be utilized to shunt a proportion of ribosomes past the ORF5 AUG codon. We also observed utilization of an AUG codon just downstream of the ORF5 AUG codon (Fig 13C, HAR, orange peak, six-codon ORF, peptide sequence MDLACE). Access to this AUG is likely facilitated by the poor initiation context of the ORF5 AUG (cauAUGa).

After translating a very short ORF (e.g. <30 codons), the small subunit of the ribosome can remain associated with the message, resume scanning, and reinitiate translation at a downstream AUG codon [87]. After translation of a short ORF, the 40S subunit of the ribosome is not immediately competent to reinitiate, but becomes competent after scanning for some distance. Thus, after translating the short CUG-initiated ORF, it is possible that the post-termination 40S subunits can scan past the five AUG codons present within the first 44 nt of ORF5 (green +s, Fig 13C), before becoming initiation competent and able to reinitiate translation at the next available AUG codon, which is the initiation codon for the E ORF some 290 nt downstream (Fig 13C) (see also [88]). The presence of an upstream CUG-initiated short ORF is preserved in other strains of MHV, though most (other than MHV-A59) *also* have a separate AUG-initiated (albeit in a weak initiation context) short ORF that could be used to shunt even more ribosomes past the ORF5 initiation codon. These viruses also preserve a conserved absence of AUG codons (in any reading frame) throughout ORF5 except for the 5′-most 44 nt (where there are from one to five AUG codons, depending on species and strain) and the very 3′ end where the E ORF AUG is situated [88]. In contrast, related viruses such as *Betacoronavirus 1* (including bovine coronavirus and equine coronavirus) have AUG codons spaced throughout ORF5, but produce a separate mRNA for E protein expression so that bicistronic expression from the same mRNA as ORF5 is not required [89, 90]. It should be noted, however, that expression of E (but not protein 5) can occur from artificial reporters in which an additional ORF is added upstream of ORF5, and therefore appears to involve internal ribosome entry [34, 91]. It is possible that multiple strategies are used to enhance E expression. Alternatively, presence of the CUG-initiated uORF could simply be to downregulate production of protein 5.

### Translation of the I ORF

**Fig 14:**
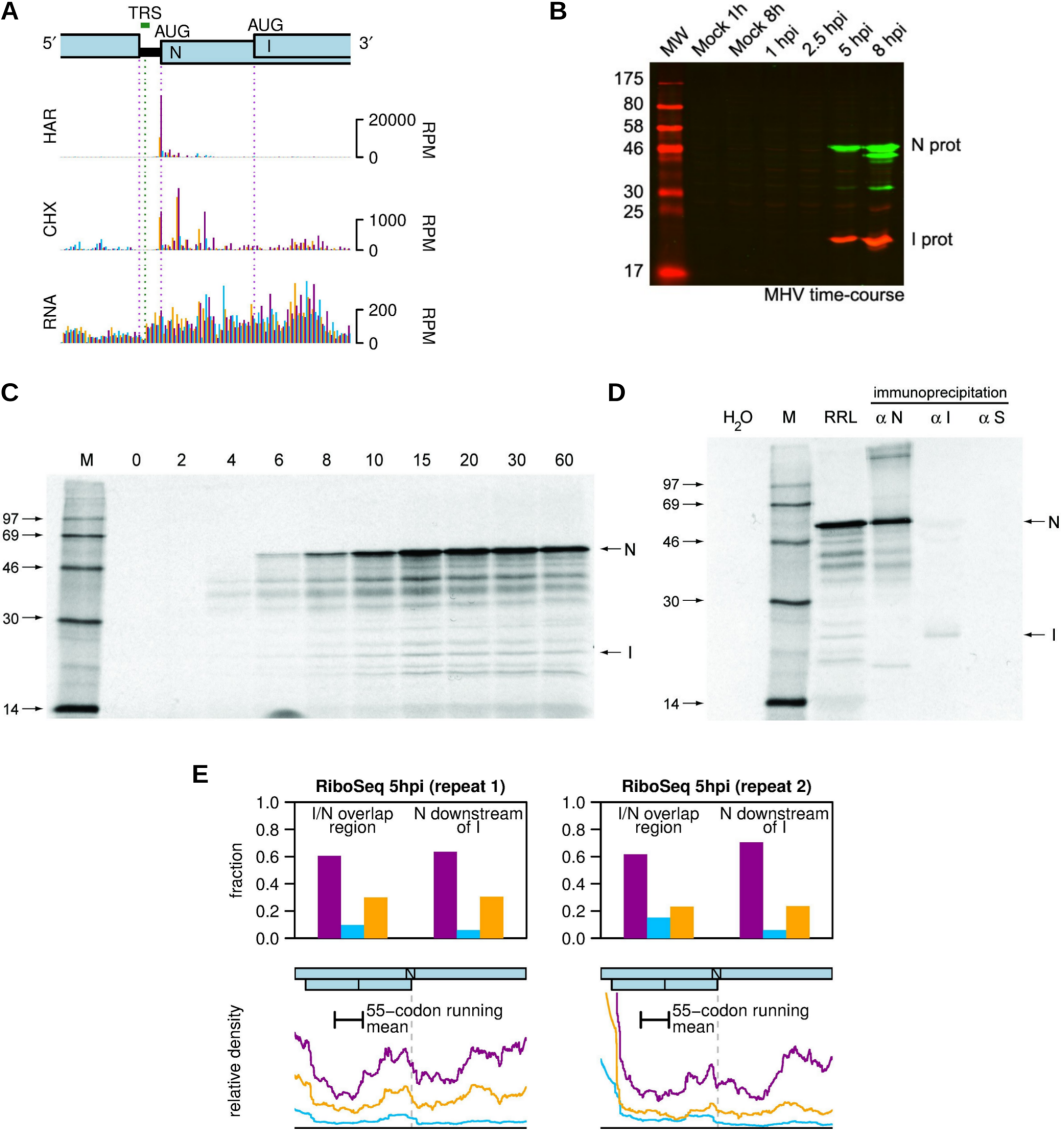
Translation of the N and I proteins. **(A)** RiboSeq (CHX and HAR) and RNASeq (RNA) counts are shown for repeat 1 at 5 h p.i. Histograms show the positions of the 5′ ends of reads with a +12 nt offset to map the approximate P-site. Reads whose 5′ ends map to the first, second or third positions of codons relative to the reading frames of the N ORF are indicated in purple, blue or orange, respectively. The I ORF is in the +1 reading frame relative to the N ORF. **(B)** I is expressed in infected-cells. 17 Cl-1 cells were infected with MHV-A59 and harvested at 1, 2.5, 5 and 8 h p.i. Cell lysates were separated on a 12% SDS-PAGE gel and immunoblotted using monoclonal anti-N and polyclonal anti-I sera. Protein molecular weight markers (MW, kDa) are indicated on the left. N and I were detected with green and red fluorescent secondary antibodies, respectively. **(C)** Time course of translation of pcDNA.3 N-ORF-derived mRNA in RRL. Translation was at 26°C and samples were collected at the indicated times prior to separation on a 10% SDS-PAGE gel. Labelled polypeptides were detected by autoradiography. Products migrating at the expected sizes for N (50 kDa) and I (23 kDa) are indicated. **(D)** The pcDNA.3 N-ORF-derived mRNA was translated in RRL and immunoprecipitated with specific anti-N, anti-I or anti-S sera. In the H_2_O control, water replaces mRNA template. Immunoprecipitated products were separated on a 10% SDS-PAGE gel and detected as above. **(E)** Top: Phasing of RPFs (CHX, 5 h p.i.) mapping to the region of the N ORF that is overlapped by the I ORF and the region of the N ORF downstream of the I ORF. Bottom: Phasing as a function of position within the N ORF smoothed with a 55-codon running-mean filter. The bar indicates 55 codons length.

A long internal ORF (I) is present within the N ORF of MHV and many other coronaviruses, encoding a largely hydrophobic polypeptide that is thought to confer a minor growth advantage to the virus [92, 93]. As shown in Fig 14A, however, HAR profiling did not reveal an initiation spike for the I protein of MHV-A59, suggesting that it might not be expressed. However, western blotting of infected-cell lysates using anti-N and anti-I sera revealed unambiguous expression of N and I from 5 h p.i. (Fig 14B). To further confirm expression of the I protein, the N coding sequence was cloned into pcDNA.3 and the mRNA translated in RRL (Fig 14C) and immunoprecipitated (Fig 14D) with anti-N and anti-I sera, and, as a negative control, anti-S serum. As shown, both N protein (50 kDa) and I protein (23 kDa) were expressed from the synthetic N mRNA, with I produced at a level of about 2% of N.

Although we were unable to obtain strong evidence for the expression of I from the profiling data, a comparison of the phasing of RPFs (a) in the region where the I ORF overlaps the N ORF, and (b) in the region of the N ORF downstream of the I termination codon, revealed in the former a slight excess of RPFs with 5′ ends mapping to the second position of N-frame codons (blue in Fig 14E; upper panels). The excess is consistent with 6–12% of ribosomes translating the +1 (i.e. I) reading frame in this region. It is possible that 5′ leader sequence present in mRNA7 (e.g. the UUG-initiated uORF), but absent from the pcDNA.3-transcribed mRNA, promote access to the I ORF. To ensure that the phasing difference was not due to a single RPF peak (as individual peaks can sometimes map to a non-standard phase; cf. Fig 9C), mean phasing was also determined in a 55-codon sliding window and, consistent with the previous result, the proportion of RPFs mapping to the second position of N-frame codons (blue in Fig 14E; lower panels) was found to decrease abruptly around the I ORF stop codon. A caveat to note is that, in MHV-A59, an upstream AUG (bold) is present in the I frame followed by a stop codon (asterisk) prior to the “designated” I AUG codon (underlined;…**M**PVAEAPL*TALVMESSRRP; both AUGs are in a strong context). In some related virus sequences, the stop codon is replaced with a sense codon such that I is probably initiated from the upstream AUG. Thus, MHV-A59 may be somewhat defective with regards to I expression.

## Discussion

We have used ribosome profiling to investigate virus gene expression kinetics, relative translational efficiencies, ribosomal frameshifting, ribosome pausing, and uORF translation in cells infected with MHV, a representative of the betacoronavirus genus of the coronavirus family of RNA viruses. These studies provide the highest resolution data on coronavirus translation to date. Using parallel RNASeq data, we examined the kinetics of virus replication and transcription, the relative abundances of different transcripts, and the degree of promiscuous polymerase jumping. We explored a number of data quality issues that can arise when applying ribosome profiling to the study of RNA viruses that replicate to high titres in cell culture and describe ways to bioinformatically assess and quantify potential contamination. Despite identifying low levels of different types of contamination, we were able to use impartial tests to avoid drawing incorrect conclusions from our data.

Viruses present particular challenges in profiling experiments. One of these is library contamination, which in this study may have been derived from two sources. The first was low-level contamination of one sample by another, a problem that is compounded by the high levels of virus RNA synthesised in infected cells at late time points. We took precautions to avoid this source of contamination, including the use of designated work spaces, buffers and equipment, and avoiding parallel processing of early and late time points where possible. Potentially, contamination may also have been introduced through the multiplex adaptor sequences. In the relatively small number of published studies on virus ribosome profiling, data from mock-infected samples and tests for contamination are often not reported, so the level of contamination suffered by others is uncertain. A second potential source of contamination could derive from RNPs comprising virus or host mRNA complexed with virus or stress-induced host RNA binding proteins. Such RNPs might co-sediment with ribosomes during the sucrose cushion centrifugation step and contaminate RiboSeq libraries. Although we were mindful of the possibility of such contamination, we found little evidence for it occurring as a result of MHV infection. An increased 3′ UTR RiboSeq (CHX) density was not apparent until 8 h p.i. (when the plateau of virus production has been reached and virtually all cells are involved in extensive syncytium formation) and, even then, the read length distributions were similar to those of mock-infected cells; suggesting that the increased 3′ UTR occupancy was as much due to *bona fide* RPFs as contaminating RNPs. The former could be due to depletion of ribosome recycling factors resulting in increased amounts of unrecycled post-termination ribosomes accessing the 3′ UTR [31]. The high level of phasing in our RiboSeq data (S4 Fig) allowed us to carefully assess contamination issues, and our observations reinforce the essentiality of basic data quality checks (e.g. S4–S10 Figs) in profiling studies. Despite these challenges, the profiling and RNASeq analysis of MHV infection still showed itself to be a powerful tool to investigate specific aspects of MHV replication at high resolution.

The kinetics of virus transcription in MHV-infected cells as observed through RNASeq were consistent with previous studies [33, 94–96]. Up to 2.5 h p.i., there was little amplification of positive-sense RNA, whilst negative-sense RNA levels rose from undetectable to about 0.1% of total virus RNA and host mRNA. Subsequently, positive-sense RNA levels increased rapidly—with the accumulation of negative-sense RNA plateauing at about 5 h p.i.—such that, at late time points, the former comprised 80–90% of total virus RNA plus host mRNA, while the latter comprised only ~0.3%. Despite differences in abundance, the patterns of expression of positive and negative-sense RNAs were similar, including high densities in the leader region, consistent with discontinuous transcription occurring during negative-strand synthesis [23]. The measurement of decumulated RNASeq densities and the analysis of specific RNASeq leader/body chimeric reads at TRSs determined the relative abundance of mRNAs at 5 h p.i. to be mRNA7 > mRNA6 > mRNA1/mRNA5/mRNA3 > mRNA4/mRNA2. An earlier study of MHV-A59 transcription using [^32^P] pulse labelling in the presence of actinomycin D provided a similar but slightly different order (mRNA7 > mRNA6 > mRNA5 > mRNA1/mRNA3/mRNA4 > mRNA2) [97] although it should be noted that, while mRNAs 7 and 6 are nearly always the most abundant subgenomic transcripts, the relative abundances of the other transcripts can vary greatly between different isolates, strains and mutants of MHV [42, 97].

The translation of virus proteins was detectable at a very early stage of infection. Indeed, using a high MOI infection, we were able to visualize input gRNA translation at 1 h p.i., a stage when the majority of ribosomes had not yet reached the pp1b ORF. Using RiboSeq (HAR) data at this time point, we were able to estimate a translation rate of 4.2 amino acids s^−1^, consistent with previous estimates for mammalian systems [8]. During the course of infection, we found that virus mRNAs 2–7 were translated with generally similar efficiencies and, importantly, were not preferentially translated relative to host mRNAs. Rather, the synthesis of large quantities of virus proteins, especially N, is achieved through high levels of transcription (note that, due to library normalization, the quotient of RiboSeq and RNASeq does not inform on global virus-induced host shut-off, which is likely to be occurring at late time points of infection [98]). The virus genomic RNA, however, appears to be poorly translated, as judged by the quotient of RiboSeq and RNASeq. During infection, much of the gRNA pool may, of course, be unavailable for translation. At earlier time points, it is, perhaps, sequestered in replication-transcription complexes; whereas at later time points, it may also be involved in packaging complexes. At 2.5 h p.i., when gRNA is unlikely to be a substrate for packaging, its translational efficiency was still low, but at this point in the replication cycle, the formation of replication-transcription complexes would preclude the massive amplification of viral RNA that takes place between 2 and 6 h p.i. [33]. It may also be the case that the pp1a and pp1b ORFs on the gRNA are inherently poorly translatable, e.g. due to translation of the uORF (see below) inhibiting ribosomal access to ORF1a.

We also observed significant amounts of RNASeq reads mapping to the N ORF region at 1 h p.i., a time point at which negative-sense RNASeq reads were essentially absent. This suggests that the N ORF RNA is not newly synthesised. Further, the absence of similar amounts of RNASeq density in the leader region, together with a very low translation efficiency, suggest that the N ORF RNA does not correspond to *bona fide* mRNA7. There has been considerable debate regarding the presence of subgenomic RNAs in coronavirus particles [99, 100] but recent analyses [101] suggest that there is a very selective incorporation of MHV gRNA into virus particles and, although immunopurified virus particles may contain detectable amounts of mRNA7, it is minimal. The N ORF RNA observed in our study may represent a part of a defective viral genome with some structural similarity to DI-like RNAs. An alternative possibility, namely that the RNASeq density corresponding to the N ORF may arise by selective degradation of the genomic RNA, is not without precedent in other virus infections [102]. However, it seems very unlikely to occur to ~96% of the input gRNA prior to replication complex formation. Further studies are needed to determine the source of this RNA and whether or not it has any biological relevance.

Our data indicate that in MHV-infected cells, in addition to the “standard” coding sequences, ribosomes access and translate a number of short ORFs. In general, translation of upstream short ORFs (uORFs) is thought to regulate translation of downstream protein-coding ORFs, with the peptide product of the uORF only rarely being functional in itself [82]. The AUG-initiated uORF of the gRNA has been characterised previously and may play a role in attenuation of translation of ORFs 1a and 1b, with a beneficial but non-essential role in coronavirus replication in cell culture [80, 103]. We found that translation of this uORF occurred at a level similar to that of ORF1a, reflecting its upstream position but poorer initiation context. Interestingly, ribosomes on this uORF paused predominantly at the second codon (proline), probably as a consequence of the restrained geometry of this amino acid in the decoding centre [83].

Other translated uORFs included a UUG-initiated 1-codon ORF in the leader sequence, an AUU-initiated 15-codon ORF upstream of ORF4a, and a CUG-initiated 13-codon ORF upstream of ORF5. The function, if any, of the first two is unknown, but we speculate that the latter uORF may play a role in expression of the E protein, which is encoded downstream of ORF5 on mRNA5. E is a small, hydrophobic viroporin that plays multiple roles during infection, including a role in virion morphogenesis [104]. As the second cistron on mRNA5, it is not clear how the E AUG is accessed for translation initiation. Previous evidence indicates that E can be expressed via internal ribosome entry [91], although the experiments that led to this conclusion did not test for the production of alternative transcripts that might allow E expression in the system used. We now hypothesize, however, that E could also be expressed via a form of leaky scanning, where, after translating the short uORF on mRNA5, the small subunit of the ribosome remains associated with the mRNA, resumes scanning, and re-initiates at the AUG of the E ORF. Intervening AUGs within the 5′ 44 nt of ORF5 could be bypassed, as the scanning 40S subunit may not have had time to reacquire the relevant initiation factors [87]. We were also able to confirm expression of the previously characterized internal (I) ORF embedded within the N gene [93] through western blotting, while analysis of profiling data (taking advantage of the phasing quality to gauge translation levels in different frames) was consistent with translation of I at a level not more than 12% of N protein expression. The mechanism of I expression is uncertain, but leaky scanning of ribosomes that fail to initiate at the N AUG is a possibility and the low level of I expression is consistent with such a mechanism. Note that failure to detect I ORF initiation (and weak detection of E ORF initiation) may indicate a shortcoming of the ribosomal profiling technique in the detection of initiation codons accessed by non-standard mechanisms.

Coronavirus −1 PRF signals have been useful models for studies of ribosomal frameshifting *in vitro*, both from the perspective of structure-function relationships of RNA pseudoknots, and also because they stimulate efficient frameshifting [58]. From the profiling analysis presented here, we now know that frameshifting in the context of MHV infection is also extremely efficient, with around half of the ribosomes that translate ORF1a continuing on to translate ORF1b. We find little evidence that −1 PRF is modulated by MHV infection, with similar efficiencies observed both in infected cells and in transfected cells expressing a frameshift-reporter mRNA. Intriguingly, there is no evidence that ribosomes pause upon encountering the MHV frameshift-promoting pseudoknot. Several published *in vitro* studies have shown that RNA pseudoknots (and certain other RNA structures) can pause ribosomes [57, 59–61] and recent kinetic studies have revealed that the translocation step of protein synthesis is significantly slowed by 3′ frameshift-stimulatory RNA structures [62–64]. Whilst the *in vitro* systems used to study pausing and frameshifting kinetics could be inappropriate, it may be that profiling is insufficiently sensitive to register what may, *in vivo*, be pseudoknot-induced ribosomal pauses of short duration. Relevant to this, despite the burgeoning literature on ribosomal profiling, only relatively few studies have addressed whether RiboSeq pauses can be generally correlated with intra-mRNA structure [105–107]. Until this is better understood, the significance of these observations remains to be determined.

In this study, we did identify a number of strong ribosomal pauses, however, and confirmed the occurrence of pausing within nsp3 in an *in vitro* translation assay. The nsp3 pausing site is located in the linker region between two modular domains of the protein, i.e. ADRP [108] and the recently identified DPUP [76], and we hypothesize that the pause may occur after synthesis of the first domain in order to allow it to fold properly before synthesis of the second domain. Ribosomal pausing as a way to optimize protein folding has been reported increasingly in recent years [109–111]. We show that replacing four residues (Lys, Arg, Lys, Phe) in the nascent peptide sequence (within 10 aa upstream of the pausing P-site) is sufficient to largely abrogate pausing, indicating that the pause is nascent peptide mediated and depends, at least in part, on positively charged residues acting within the ribosome exit tunnel, consistent with other ribosome profiling data where positively charged residues have been linked to ribosome retardation [77].

Our analysis of MHV by ribosomal profiling is the first such investigation for an RNA virus. Together with RNASeq, the datasets provide a high-resolution examination of MHV replication and gene expression and provides a basis for the subsequent analyses of virus-host responses (manuscript in preparation). We anticipate that the information will also be valuable to researchers with an interest in translation and virology, not least due to the excellent phasing in the RiboSeq datasets and the good coverage of reads on virus and cellular mRNAs.

## Materials and Methods

### Cells and virus

Murine 17 clone 1 (17Cl-1) [112] and BHK-21 [C-13] (ATCC CCL-10) cells were maintained in Dulbecco’s modification of Eagle’s medium supplemented with 10% (vol/vol) fetal calf serum (FCS). Recombinant MHV strain A59 (MHV-A59) was derived as previously described [113]. 17Cl-1 cells (10^7^) were plated in 10 cm dishes and, upon reaching 70–80% confluence, were infected with MHV-A59 at a multiplicity of infection (MOI) of 10 PFU/cell (or 200 PFU/cell in the “High MOI” experiment) in Hank’s balanced salt solution (HBSS) containing 50 μg/ml DEAE-dextran and 0.2% bovine serum albumin (BSA). After 45 min at 37°C, the inoculum was removed and the cells were incubated in DMEM containing 10% FCS, 100 U/ml penicillin and 100 μg/ml streptomycin at 37°C until harvest.

### Drug treatment and lysis

At the appropriate time point, cells were treated with CHX (Sigma-Aldrich; to 100 μg/ml; 2 min), or HAR (LKT laboratories; 2 μg/ml, 3 min) then CHX (to 100 μg/ml; 2 min). Cells were rinsed with 5 ml of ice-cold PBS, the dishes were submerged in a reservoir of liquid nitrogen for 10 s and then transferred to dry ice and 400 μl of lysis buffer [20 mM Tris-HCl pH 7.5, 150 mM NaCl, 5 mM MgCl_2_, 1 mM DTT, 1% Triton X-100, 100 μg/ml cycloheximide and 25 U/ml TURBO DNase (Life Technologies)] dripped onto the cells. The cells were scraped extensively to ensure lysis, collected and triturated with a 26-G needle ten times. Lysates were clarified by centrifugation for 20 min at 13,000 g at 4°C, the supernatants recovered and stored in liquid nitrogen.

### Ribosomal profiling and RNASeq

Cell lysates were subjected to RiboSeq and RNASeq. The methodologies employed were based on the original protocols of Ingolia and colleagues [7, 114], except ribosomal RNA contamination was removed by treatment with duplex-specific nuclease (DSN) and library amplicons were constructed using a small RNA cloning strategy [115] adapted to Illumina smallRNA v2 to allow multiplexing. The methods used were as described [16], with minor modifications for the analysis of ribosomal pausing at the MHV −1 PRF signal, namely a broader range of RPFs, migrating between 28 and 80 nt, were harvested prior to amplicon construction, and longer PCR amplicons of ~150–206 bp were gel purified. Amplicon libraries were deep sequenced using an Illumina HiSeq 2000 platform (repeat 1 samples at the Wellcome Trust Centre for Human Genetics—Oxford Genomics Centre; repeat 2, MOI 200, and long read samples at the Beijing Genomics Institute).

### Computational analysis of RiboSeq and RNASeq data

Adaptor sequences were trimmed using the FASTX-Toolkit and reads shorter than 25 nt were discarded. Trimmed reads were mapped first to *Mus musculus* rRNA (GenBank accession numbers NR_003278, NR_003279, NR_003280, NR_030686, NR_046233 and GU372691), followed by the MHV genome (GenBank accession number AY700211.1) and subsequently *Mus musculus* mRNA, ncRNA and genomic DNA databases. In order to select good-quality samples of host mRNA-derived RPFs for analyzing RPF length, framing, and position-on-transcript distributions, the mouse mRNA database comprised NCBI RefSeq mRNAs. The non-coding RNA and genomic DNA databases comprised the Ensembl Mus_musculus.NCBIM37.64.ncrna.fa and release-64 DNA chromosome files, respectively. Reads that map to the gDNA, but none of the RNA databases, are expected to derive from unannotated transcripts as the sequencing protocol is RNA-specific. Reads were mapped using bowtie version 1 [116] with parameters -v 2 —best (i.e. maximum 2 mismatches, report best match). The order of mapping was tested to check that virus-derived reads were not lost accidentally due to mis-mapping to host RNA, or *vice versa*; a slight reduction (~0.05%) in virus-derived reads was observed only on mapping to the entire host genome (gDNA) and thus mapping to virus RNA and host mRNA was considered to be specific. For host mRNA mapping, no specific consideration was given to the presence of multiple isoforms within the RefSeq database; reads that could be mapped to multiple transcripts were assigned at random to one transcript. Except where specifically stated, virus reads that mapped discontinuously to the MHV genome (due to transcriptional discontinuities at TRS sites) were excluded from the analyses.

Host mRNA RiboSeq and RNASeq phasing distributions (S2 Fig and S3 Fig) were derived from reads mapping to the “interior” regions of annotated coding ORFs; specifically, the 5′ end of the read had to map between the first nucleotide of the initiation codon and 30 nt 5′ of the last nucleotide of the termination codon, thus, in general, excluding RPFs of initiating or terminating ribosomes. Histograms of 5′ end positions of host mRNA reads relative to initiation and termination codons (S4 Fig, S5 Fig, S6 Fig) were derived from reads mapping to RefSeq mRNAs with annotated CDSs ≥450 nt in length and annotated 5′ and 3′ UTRs ≥60 nt in length. All figures are based on total numbers of mapped reads, rather than weighted sums for highly expressed mRNAs [7], because virus-induced shut-off of host cell translation at late time points reduces the efficacy of the latter approach for our data. Read length distributions (S7 Fig and S8 Fig) are based on total mapped reads (to positive-sense host mRNA, or to positive or negative-sense MHV genome, as indicated) without restriction to annotated coding regions. To compare read densities between CDSs and 3′ UTRs (S9 Fig and S10 Fig), we used reads whose 5′ end offset by +12 nt (i.e. estimated P-site positions for RPFs) mapped within the regions from 30 nt to 300 nt upstream of stop codons (CDSs), or from 30 nt to 300 nt downstream of stop codons (3′ UTRs). This analysis was restricted to mRNAs with annotated coding ORFs ≥450 nt in length and annotated 3′ UTRs ≥300 nt in length. The presence of transcript isoforms with 3′ UTRs shorter than the annotated (≥300 nt) 3′ UTRs leads to a modest underestimation of the actual 3′ UTR density.

For Fig 12B, RefSeq mRNAs with annotated CDSs ≥300 nucleotides in length and annotated 5′ UTRs ≥30 nt in length (with no restriction on annotated 3′ UTR length) were used, as only the 5′ end of CDSs was analysed, and the more relaxed thresholds increased the sample size [important for the more restricted set of CDSs beginning with AUG-CCN (Met-Pro); of 29600 NCBI RefSeq mRNA accessions, 1558 have CDSs beginning with AUG-CCN]. Transcripts with ≥20 RPFs with 5′ ends mapping between −30 and +15 relative to the annotated initiation codon were used, and histograms of 5′ end positions for individual transcripts were down-weighted by the number of RPFs mapping to this region before summing over the different transcripts (i.e. a weighted sum of “highly expressed” mRNAs, [7]). Fig 12B is based on sums over 3620 and 203 transcripts for generic CDSs and CDSs beginning with AUG-CCN, respectively.

Plots showing reads mapped to the MHV genome (Figs 1, 2, 7, 9, 11, 12A, 13 and 14 and S11 Fig) show histograms of the positions to which the 5′ ends of reads map, with a +12 nt offset to indicate (for RPFs) the approximate P-site. (More precisely, the +12 nt offset means that RPFs whose 5′ end aligns to the first position of a codon are mapped to the first nucleotide of the P-site codon, and RPFs whose 5′ end aligns to the third position of a codon are mapped to the last nucleotide of the codon preceding the P-site codon.) In contrast, plots showing reads summed over large numbers of host mRNAs (Fig 12B and S4 Fig, S5 Fig, S6 Fig) show histograms of the positions to which the 5′ ends of reads map, without the +12 nt offset. This is because the host mRNA plots are used for calibration whereas the virus plots are used to illustrate specific features of virus gene expression. To normalize for different library sizes, while taking into account global shut-off of host gene expression in response to virus infection, counts expressed as reads per million mapped reads (RPM) or reads per kb per million mapped reads (RPKM) use the sum of total virus RNA (positive and negative-sense) plus total host mRNA (reads that map to NCBI mRNA RefSeqs) as the denominator. The same library normalization factors were also used for Fig 3, S4 Fig and S6 Fig.

To calculate the expression of individual virus ORFs (Fig 4, RiboSeq), we counted RPFs whose 5′ end mapped between the first nucleotide of the initiation codon and 30 nt 5′ of the termination codon, thus excluding RPFs of ribosomes paused during initiation or termination (or nearby). The corresponding sequence length was used to calculate counts per kb. We used a similar procedure to calculate RNASeq densities for each inter-TRS region (Fig 4, RNASeq), with the inter-TRS regions (prior to the 30 nt 3′ buffer) being 72 to 21748 (mRNA1), 21754 to 23923 (mRNA2), 23929 to 27936 (mRNA3), 27942 to 28319 (mRNA4), 28325 to 28959 (mRNA5), 28965 to 29656 (mRNA6), and 29662 to 31335 (mRNA7).

Frameshifting efficiencies (Fig 8) were calculated using reads whose 5′ end offset by +12 nt (i.e. estimated P-site positions for RPFs) mapped within the regions 361 to 13452 (for ORF1a) and 13774 to 21596 (for ORF1b). These coordinates leave a 150 nt buffer after the ORF1a initiation codon (nt 211), before the frameshift site (nt 13602), after the ORF1a termination codon (nt 13623) and before the ORF1b termination codon (nt 21746), respectively. Read counts were divided by region lengths to obtain read densities. Phasing distributions in the N and I ORFs (Fig 14E) were calculated with respect to the N reading frame, using reads whose 5′ end offset by +12 nt (i.e. estimated P-site positions) mapped within the regions 29736 to 30353 (for the I/N overlap) and 30360 to 31031 (for N downstream of I). For comparison, the coordinates of the N and I ORFs are 29670 to 31034 (N) and 29734 to 30357 (I). For the analysis of RiboSeq and RNASeq count variability within ORF1a (Fig 9A), counts were first smoothed with a 3-nt running mean filter and then the fold-change relative to mean was calculated using reads whose 5′ end mapped between nt 211 (the start of ORF1a) and nt 13572 (30 nt 5′ of the frameshift site).

For the above analyses, virus reads with discontiguous mappings to the MHV genome (i.e. reads spanning sites of discontinuous transcription—generally at the TRS sites) were excluded. To identify such reads we re-mapped raw trimmed reads to host rRNA, virus genome, host mRNA, ncRNA and gDNA databases, this time permitting zero mismatches. We then pooled the remaining unmapped reads with the reads that mapped to the virus genome and, for each library, searched this set of reads for the query sequence UUUAAAUCUAA (AY700211.1 nt 55 to 65; 5′-adjacent to the leader TRS). Reads were selected that had at least 17 nt 3′ of the query sequence and classified according to whether nucleotides +3 to +17 after the query sequence were compatible with mRNA1, 2, 3, 4, 5, 6 or 7, or were derived from non-canonical chimeric sequences. These criteria were motivated by previous data indicating that, in leader/body chimeras, nucleotides up to and including UUUAAAUCUAA are templated by the leader, nucleotides at +1 and +2 may be templated by leader *or* genome, and nucleotides at +3 and above are templated by the genome sequence [41]. Counts were normalized to reads per million mapped reads as described above. A possible source of error here is that different libraries have different RNASeq read length distributions (due to variation in the gel-slice boundaries); libraries with longer reads will have proportionally more reads found to span leader/body discontinuities due to the requirement of at least 17 nt 3′ of the 11-nt query sequence for selection. For this reason, inter-library comparisons are avoided.

To calculate host translational efficiencies, after removing reads mapping to rRNA with bowtie1 as above, remaining reads were mapped to the mouse genome (UCSC, assembly mm10) using TopHat (parameters: —no-novel-juncs —bowtie1 —prefilter-multihits —max-multihits 500, with —transcriptome-index defined using the genes.gtf file from the UCSC mm10 annotation available from the tophat website) [117]. Reads entirely contained within annotated CDSs were enumerated with htseq-count (parameters: -t CDS -m intersection-strict -i gene_id -s yes) [118], reporting read counts per gene rather than per transcript. Read counts were normalized for library size as above, and for CDS length according to the sum of all coding exon fragment lengths for a given gene ID in the genes.gtf file. This will tend to result in an overestimate of CDS lengths since many transcripts (alternative splice forms and/or alternative transcription initiation sites) will lack some coding exons. While this is likely to have only a modest effect on RiboSeq/RNASeq translation efficiencies (Fig 6, *y*-axis) it will tend to result in underestimates for RNASeq RPKM values (Fig 6, *x*-axis).

The sequencing data have been deposited in the ArrayExpress database (http://www.ebi.ac.uk/arrayexpress) under the accession number E-MTAB-4111.

### Plasmids

The MHV frameshift signal, and the N, nsp3 and nsp6 protein coding sequences were amplified using specific oligonucleotides (S4 Table) and cDNA derived from 17 Cl-1 cells infected with MHV-A59 at an MOI 10 and harvested at 8 h p.i. For assessing frameshifting efficiencies in transfected tissue culture cells, the dual-luciferase reporter vector pDluc was employed (kind gift from Dr M. Howard, University of Utah; [53]). DNA fragments of 100 bp spanning the MHV frameshift signal and flanked by *Xho*I and *Bgl*II restriction sites were derived by PCR amplification and ligated into appropriately cleaved pDluc vector. An in-frame control (mimicking 100% frameshifting efficiency) was also constructed. pDluc-IBV and pDluc-HXB2 have been described elsewhere [54, 55]. *Bam*H1-*Xho*I-digested PCR fragments were cloned into pcDNA 3.1 (+) (Life Technologies) previously digested with *Bam*H1-*Xho*I. In pPS0 plasmids, PCR reactions were carried out using the pcDNA.3 nsp3 plasmid as a template and cloned into a digested *Xho*I/*Pvu*II-pPS0 plasmid. pPS0-nsp3 mutants were subjected to site-directed mutagenesis. For all pcDNA.3 and pPS0 constructs, a “pause control” was also generated in which a UAA stop codon was introduced to generate a protein whose size corresponded to that produced by the predicted ribosomal pause. All sequences were confirmed by dideoxy sequencing.

### Frameshifting assays in tissue culture

17 Cl-1 and BHK-21 cells were seeded in dishes of a 24-well plate and grown for 16 h until 80% confluence was reached. Plasmids were transfected using a commercial liposome method (TransIT-LT1, Mirus). Transfection mixtures [containing plasmid DNA, serum-free medium (Opti-MEM; Gibco-BRL) and liposomes] were set up as recommended by the manufacturer and added dropwise to the tissue culture cell growth medium. Cells were harvested 24 h post transfection (h.p.t.) and reporter gene expression was determined using a dual-luciferase assay system kit (Promega). Frameshifting efficiencies were calculated by dividing the Fluc/Rluc ratios of the test samples by the Fluc/Rluc ratio of the in-frame controls.

### Immunoblotting

Proteins were separated by 10%, 12% or 15% SDS-PAGE depending on the molecular weight of the protein of interest and transferred to nitrocellulose membranes. These were blocked for 30–60 min with 5% powdered milk (Marvel) in PBST [137 mM NaCl, 2.7 mM KCl, 10 mM Na_2_HPO_4_, 1.5 mM KH_2_PO_4_ (pH 6.7), and 0.1% Tween 20] and probed with mouse monoclonal antibodies raised against nsp9 (AM08450PU-N, Acris Antibodies, Inc, 1:500 in Marvel-PBST), N (1:1,000), S (1:500), GAPDH (G8795, Sigma-Aldrich, 1:20,000) or a polyclonal rabbit anti-I (1:1,000, a kind gift of Prof. P. S. Masters, Wadsworth Center, New York State Department of Health). Membranes were incubated in the dark with an IRDye-conjugated secondary antibody in PBST [IRDye 800CW Donkey Anti-Mouse IgG (H+L), IRDye 800CW Donkey Anti-Rabbit IgG (H+L) and IRDye 680RD Goat Anti-Mouse IgM (μ chain specific)]. Blots were scanned and bands quantified using an Odyssey Infrared Imaging System (Licor).

### *In vitro* transcription, translation and immunoprecipitation

pcDNA.3 and pPS0 plasmids were linearized with *Xho*I and *Ava*II respectively and capped run-off transcripts generated using T7 RNA polymerase and SP6 RNA polymerase respectively as described previously [119]. RNAs were recovered by a single extraction with phenol-chloroform (1:1 vol/vol) followed by ethanol precipitation. Remaining unincorporated nucleotides were removed by gel filtration through a NucAway spin column (Ambion). The eluate was concentrated by ethanol precipitation, the mRNA resuspended in water, checked for integrity by agarose gel electrophoresis and quantified by spectrophotometry. RNAs were translated in nuclease-treated rabbit reticulocyte lysate (RRL) (Promega) programmed with ~50 μg/ml template mRNA. A typical reaction mixture had a volume of 10 μl and was composed of 90% (vol/vol) RRL, 20 μM amino acids (lacking methionine), and 0.2 MBq [^35^S]-methionine. Reaction mixtures were incubated for 30 min at 26°C and stopped by the addition of an equal volume of 10 mM EDTA, 100 μg/ml RNase A followed by incubation at room temperature for 15 min. In ribosomal pausing assays, conditions were the same except that the reaction mixture had a volume of 40 μl and the translational inhibitor edeine was added 3 min after the start of the reaction in order to obtain synchronous initiation (final concentration, 5 μM). Aliquots of 1.5 μl were withdrawn from the translation reaction mixture at specified intervals and mixed with an equal volume of EDTA/RNase A mixture, as above. In immunoprecipitations, 10 μl of RRL was mixed with either mouse anti-N, anti-S or rabbit anti-I for 30 min at 4°C prior to binding to protein A-Sepharose CL-4B (Pharmacia Biotech AB, Uppsala, Sweden) and subsequent washing. Samples were prepared for SDS-PAGE by addition of 10 volumes of 2X Laemmli’s sample buffer and boiling for 4 min. Proteins were resolved on 10% or 15% SDS-PAGE gels. ^14^C-labelled molecular weight standards (MW) were from Amersham International (United Kingdom). Dried gels were exposed to a Carestream Kodak Biomax MR film (Sigma-Aldrich) and scanned.

## Supporting Information

S1 Table Library composition statistics. Table of host and virus read counts for the different samples.
(DOCX)

S2 Table Genomic sequences flanking the leader and body junction sites. TRSs (UCUAAAC or similar) are indicated in bold. Nucleotides consistent with tandem copies of the pentanucleotide UCUAA are indicated in red (copy at the canonical junction site) and blue (copy 5 nt upstream of the canonical junction site). Note also the high similarity between the sequences at the leader (mRNA1) and mRNA7 junction sites: when polymerase jumping for mRNA7 occurs 5 nt upstream of the canonical site, 17 nt of 3′ sequence are required to distinguish gRNA reads from mRNA7 reads.
(DOCX)

S3 Table Frequencies of canonical and non-canonical leader/body chimeric reads. Chimeric reads utilizing the leader TRS were identified by searching for all reads containing the sequence UUUAAAUCUAA (AY700211.1 nt 55 to 65), and classified according to the identity of the following nucleotides at positions +3 to +17. These 15 nucleotides are listed in column 4. The genomic coordinate of the first nucleotide of the 15 is given in column 5. Nucleotides at positions +1 to +2 in the RNASeq read are listed in column 3. The corresponding two nucleotides from the genome are listed in column 2. Also, the 5 nucleotides preceding these in the genome are listed in column 1. The numbers of junction/body chimeric reads containing each sequence are given in column 6 (repeat 1) and column 7 (repeat 2). Only sequences with three or more occurrences in repeat 1 *and* ten or more occurrences in repeat 2 are shown. Data are shown for the 5 h p.i. RNASeq libraries.
(DOCX)

S4 Table List of oligonucleotides used. Note *fwd* indicates forward primer, and *rev* indicates reverse primer.
(DOCX)

S1 Fig Composition of the time-course libraries. Reads were mapped to virus RNA, and host rRNA, mRNA, ncRNA and gDNA databases. Reads mapping to gDNA are expected to derive from unannotated transcripts not present in the mRNA or ncRNA databases, but, since the direction of transcription is not annotated in the gDNA database, such reads constitute a mixture of forward and reverse-sense matches. Reverse-sense rRNA matches in the RNASeq samples are expected to derive from the RiboZero kit which contains complementary sequences to rRNA.
(TIF)

S2 Fig Phasing of reads mapping to host mRNAs (repeat 1). Phasing of 5′ ends of reads that map to host mRNA coding regions as a function of read length. Reads whose 5′ ends map to the first, second or third positions of codons are indicated in purple, blue or orange, respectively.
(TIF)

S3 Fig Phasing of reads mapping to host mRNAs (repeat 2). See S2 Fig caption for details.
(TIF)

S4 Fig RPF distributions on host mRNAs. Histograms of RPF 5′ end positions relative to annotated initiation and termination codons summed over all host RefSeq mRNAs for the RiboSeq libraries. To account for different library sizes, histograms are normalized by the sum of total virus RNA (positive and negative-sense) plus total host mRNA for the library.
(TIF)

S5 Fig Example of offset-to-P-site calibration. Histograms of RPF 5′ end positions relative to annotated initiation and termination codons summed over all host RefSeq mRNAs for the RiboSeq CHX 5 h p.i. time point (repeat 1) as a function of RPF length. RPFs of ribosomes paused during initiation with the initiation codon (AUG at position 0 to 2; left) in the P-site have 5′ ends that normally map to position −12 (12 nt upstream), or, particularly for longer RPFs (e.g. 30–32 nt), position −13. The smaller peak at 0 is likely an artifact of ligation bias (and potentially also nuclease bias)—all RPFs mapping to this position begin with 5′-AUG, whereas RPFs that map to other positions have differing 5′ end nucleotides so that any 5′-end-dependent biases are averaged out when summing over many mRNAs. Termination occurs with the stop codon (UNN at position −2 to 0; right) in the A-site so that the 5′ ends of RPFs paused during termination normally map to position −17 (15 nt upstream).
(TIF)

S6 Fig RNASeq distributions on host mRNAs. Histograms of read 5′ end positions relative to annotated initiation and termination codons summed over all host RefSeq mRNAs for the RNASeq libraries. To account for different library sizes, histograms are normalized by the sum of total virus RNA (positive and negative-sense) plus total host mRNA for the library.
(TIF)

S7 Fig Comparison of read length distributions for virus and host mRNA (repeat 1). Length distributions for reads mapping to host mRNAs (green), positive-sense virus RNA (orange) and negative-sense virus RNA (blue, dashed) for repeat 1. The left panel in each pair shows the absolute read counts. The right panel in each pair shows the distributions normalized to have equal total sums to facilitate comparison of distribution shapes. Differences between host and virus distributions are indicative of contamination. Negative-sense virus read length distributions are only shown at 5 h p.i. and 8 h p.i. as the counts at earlier time points are often too low to assess distribution shape.
(TIF)

S8 Fig Comparison of read length distributions for virus and host mRNA (repeat 2). See S7 Fig caption for details.
(TIF)

S9 Fig Comparison of the density and length distributions of reads mapping to host mRNA coding regions and 3′ UTRs (repeat 1). Reads were counted in windows from 10 to 100 codons upstream (CDS; green) or downstream (3′ UTR; orange) of annotated termination codons, and summed over all host mRNAs. The left panel in each pair shows the absolute read counts, allowing comparison of the CDS and 3′ UTR read densities; the density ratio (3′ UTR / CDS) is indicated in purple in each panel. For all RiboSeq samples, 3′ UTR occupancy is very low compared to CDS occupancy, whereas, for RNASeq, 3′ UTR occupancy is typically around 80% of CDS occupancy (the RNASeq value is less than unity due to differences in the transcript isoforms present in the sample compared to the RefSeq mRNA database). The right panel in each pair shows the distributions normalized to have equal total sums so that the shapes of the CDS and 3′ UTR distributions can be compared. For RNASeq, the two distributions have essentially identical shapes. For RiboSeq, differences in the two distributions provide an indicator of the level of non-RPF contamination present in the sample.
(TIF)

S10 Fig Comparison of the density and length distributions of reads mapping to host mRNA coding regions and 3′ UTRs (repeat 2). See S9 Fig caption for details.
(TIF)

S11 Fig Analysis of longer RPF species. Unfractionated RiboSeq RNA prepared for the 5 h p.i. time point of repeat 2 was re-run on a 15% denaturing acrylamide-urea gel and a larger gel slice taken to sample RPFs within the range 28 to ~80 nt. Histograms show the positions of the 5′ ends of reads with a +12 nt offset to map the approximate P-site. RPF distributions were smoothed with a 15-nt running-mean filter. Note the widely varying vertical axis scales—the vast majority of RPFs fall in the size range 25–35 nt. Blue triangles indicate the previously analysed sites of RPF accumulation (see Fig 9).
(TIF)

S12 Fig Data quality analysis for the high MOI samples. **(A)** Composition of the high MOI libraries (see S1 Fig caption for further details). **(B)** Comparison of read length distributions for virus and host mRNA (see S7 Fig caption for further details). **(C)** Phasing of reads mapping to host mRNAs (see S2 Fig caption for further details).
(TIF)
