# Supplementary material for: High-Resolution Analysis of Coronavirus Gene Expression by RNA Sequencing and Ribosome Profiling
Source: PLoS Pathog. 2016 Feb 26;12(2):e1005473. doi: 10.1371/journal.ppat.1005473 (PMC4769073; doi:10.1371/journal.ppat.1005473)
Supplement: S1 Table — Table of host and virus read counts for the different samples. (DOCX) [file ppat.1005473.s001.docx]

**S1 Table. Library composition statistics.** Table of host and virus read counts for the different samples.

| **Conditions** | **Time point** | **total reads** | **host mRNA** | **positive vRNA** | **negative vRNA** |
| --- | --- | --- | --- | --- | --- |
|  |  |  |  |  |  |
| **A. MOI 10 infections** | | | | | |
|  |  |  |  |  |  |
| **Repeat 1** |  |  |  |  |  |
| RiboSeq-CHX | 1 h p.i. | 38886254 | 12131105 | 3775 | 4 |
|  | 2.5 h p.i. | 48426898 | 15593464 | 23338 | 175 |
|  | 5 h p.i. | 51078923 | 10689279 | 2855949 | 1259 |
|  | 8 h p.i. | 63172375 | 1120219 | 3254224 | 1010 |
|  | mock 1 h | 38543800 | 8503067 | 994 | 1 |
|  | mock 8 h | 27124163 | 7039902 | 1911 | 1 |
| RiboSeq-HAR | 1 h p.i. | 52894123 | 11211468 | 3893 | 6 |
|  | 2.5 h p.i. | 47243830 | 9899418 | 18505 | 77 |
|  | 5 h p.i. | 58918441 | 8553614 | 2193941 | 2293 |
|  | 8 h p.i. | 55545738 | 554552 | 2347517 | 1354 |
|  | mock 1 h | 54477520 | 4648657 | 1583 | 2 |
|  | mock 8 h | 78504191 | 5626427 | 1948 | 1 |
| RNASeq-CHX | 1 h p.i. | 85025636 | 9729162 | 47948 | 330 |
|  | 2.5 h p.i. | 75119105 | 7489099 | 25257 | 1786 |
|  | 5 h p.i. | 67199222 | 6243505 | 4462952 | 41214 |
|  | 8 h p.i. | 62943574 | 3890960 | 22434260 | 97846 |
|  | mock 1 h | 80602120 | 7535385 | 975 | 3 |
|  | mock 8 h | 68586026 | 6556438 | 1032 | 7 |
| **Repeat 2** |  |  |  |  |  |
| RiboSeq-CHX | 1 h p.i. | 59549003 | 15243244 | 503 | 2 |
|  | 2.5 h p.i. | 39417504 | 3397121 | 14344 | 522 |
|  | 5 h p.i. | 43115271 | 2930254 | 5293975 | 663 |
|  | 8 h p.i. | 48052744 | 923793 | 1030917 | 4462 |
|  | mock 1 h | 55466003 | 4138685 | 3 | 1 |
|  | mock 8 h | 47434328 | 9966308 | 13 | 10 |
| RiboSeq-HAR | 1 h p.i. | 53371211 | 5049638 | 325 | 0 |
|  | 2.5 h p.i. | 63011777 | 2667612 | 1330 | 26 |
|  | 5 h p.i. | 54399813 | 2466424 | 2783276 | 35658 |
|  | 8 h p.i. | 60590162 | 1155158 | 3320814 | 34893 |
|  | mock 1 h | 51330353 | 3040164 | 2 | 0 |
|  | mock 8 h | 68578050 | 4520914 | 28 | 2 |
| RNASeq-CHX | 1 h p.i. | 67671001 | 5762057 | 21043 | 5 |
|  | 2.5 h p.i. | 79412735 | 8461362 | 34252 | 4775 |
|  | 5 h p.i. | 65839440 | 6914861 | 25450950 | 83260 |
|  | 8 h p.i. | 65886262 | 3472234 | 31182781 | 76477 |
|  | mock 1 h | 45939400 | 4556438 | 0 | 0 |
|  | mock 8 h | 62128055 | 6486670 | 55 | 0 |
|  |  |  |  |  |  |
| **B. MOI 200 infections** | | | | | |
|  |  |  |  |  |  |
| RiboSeq-CHX | 1 h p.i. | 56043345 | 22219724 | 464 | 0 |
| RiboSeq-HAR | 1 h p.i. | 50040366 | 4541453 | 509 | 0 |
| RNASeq-CHX | 1 h p.i. | 82293225 | 10297184 | 92974 | 27 |
|  |  |  |  |  |  |
| **C. MOI 10 infections (long reads)** | | | | | |
|  |  |  |  |  |  |
| RiboSeq-CHX | 5 h p.i. | 76099040 | 2725987 | 4546887 | 933 |
