# Supplementary material for: High-Resolution Analysis of Coronavirus Gene Expression by RNA Sequencing and Ribosome Profiling
Source: PLoS Pathog. 2016 Feb 26;12(2):e1005473. doi: 10.1371/journal.ppat.1005473 (PMC4769073; doi:10.1371/journal.ppat.1005473)
Supplement: S2 Table — TRSs (UCUAAAC or similar) are indicated in bold. Nucleotides consistent with tandem copies of the pentanucleotide UCUAA are indicated in red (copy at the canonical junction site) and blue (copy 5 nt upstream of the canonical junction site). Note also the high similarity between the sequences at the leader (mRNA1) and mRNA7 junction sites: when polymerase jumping for mRNA7 occurs 5 nt upstream of the canonical site, 17 nt of 3′ sequence are required to distinguish gRNA reads from mRNA7 reads. (DOCX) [file ppat.1005473.s002.docx]

**S2 Table. Genomic sequences flanking the leader and body junction sites.** TRSs (UCUAAAC or similar) are indicated in bold. Nucleotides consistent with tandem copies of the pentanucleotide UCUAA are indicated in red (copy at the canonical junction site) and blue (copy 5 nt upstream of the canonical junction site). Note also the high similarity between the sequences at the leader (mRNA1) and mRNA7 junction sites: when polymerase jumping for mRNA7 occurs 5 nt upstream of the canonical site, 17 nt of 3′ sequence are required to distinguish gRNA reads from mRNA7 reads.

| transcript | junction site and flanking sequence | genomic location | product(s) |
| --- | --- | --- | --- |
| mRNA1/gRNA | AUCUAA**UCUAAAC**UUUAU | 60-77 | 1a/1ab |
| mRNA2 | AAUAAA**UCUAUAC**UUGUC | 21742-21759 | 2 |
| mRNA3 | GCAUAA**UCUAAAC**AUGCU | 23917-23934 | S |
| mRNA4 | AGAAAA**UCUAAAC**AAUUU | 27930-27947 | 4 |
| mRNA5 | UACUAA**UCUAAAC**CUCAU | 28313-28330 | 5/E |
| mRNA6 | AUCUAA**UCCAAAC**AUUAU | 28953-28970 | M |
| mRNA7 | ACCUAA**UCUAAAC**UUUAA | 29650-29667 | N/I |
