# Supplementary material for: High-Resolution Analysis of Coronavirus Gene Expression by RNA Sequencing and Ribosome Profiling
Source: PLoS Pathog. 2016 Feb 26;12(2):e1005473. doi: 10.1371/journal.ppat.1005473 (PMC4769073; doi:10.1371/journal.ppat.1005473)
Supplement: S3 Table — Chimeric reads utilizing the leader TRS were identified by searching for all reads containing the sequence UUUAAAUCUAA (AY700211.1 nt 55 to 65), and classified according to the identity of the following nucleotides at positions +3 to +17. These 15 nucleotides are listed in column 4. The genomic coordinate of the first nucleotide of the 15 is given in column 5. Nucleotides at positions +1 to +2 in the RNASeq read are listed in column 3. The corresponding two nucleotides from the genome are listed in column 2. Also, the 5 nucleotides preceding these in the genome are listed in column 1. The numbers of junction/body chimeric reads containing each sequence are given in column 6 (repeat 1) and column 7 (repeat 2). Only sequences with three or more occurrences in repeat 1 and ten or more occurrences in repeat 2 are shown. Data are shown for the 5 h p.i. RNASeq libraries. (DOCX) [file ppat.1005473.s003.docx]

**S3 Table. Frequencies of canonical and non-canonical leader/body chimeric reads.** Chimeric reads utilizing the leader TRS were identified by searching for all reads containing the sequence UUUAAAUCUAA (AY700211.1 nt 55 to 65), and classified according to the identity of the following nucleotides at positions +3 to +17. These 15 nucleotides are listed in column 4. The genomic coordinate of the first nucleotide of the 15 is given in column 5. Nucleotides at positions +1 to +2 in the RNASeq read are listed in column 3. The corresponding two nucleotides from the genome are listed in column 2. Also, the 5 nucleotides preceding these in the genome are listed in column 1. The numbers of junction/body chimeric reads containing each sequence are given in column 6 (repeat 1) and column 7 (repeat 2). Only sequences with three or more occurrences in repeat 1 *and* ten or more occurrences in repeat 2 are shown. Data are shown for the 5 h p.i. RNASeq libraries.

| genomic sequence | | RNASeq sequence | | genomic coordinate | number | | mRNA |
| --- | --- | --- | --- | --- | --- | --- | --- |
| −5 to −1 | +1 to +2 | +1 to +2 | +3 to +17 |  | repeat 1 | repeat 2 |  |
| CCUAA | UC | UC | UAAACUUUAAGGAUG | 29658 | 3769 | 28735 | mRNA7 |
| UCUAA | UC | UC | CAAACAUUAUGAGUA | 28961 | 445 | 5055 | mRNA6 |
| UCUAA | UC | UC | UAAACUUUAUAAACG | 68 | 168 | 1571 | gRNA |
| ACUAA | UC | UC | UAAACCUCAUCUUAA | 28321 | 95 | 1129 | mRNA5 |
| CAUAA | UC | UC | UAAACAUGCUGUUCG | 23925 | 93 | 846 | mRNA3 |
| UGAUA | UC | UC | UAAUCCAAACAUUAU | 28956 | 91 | 783 | mRNA6 |
| GAAAA | UC | UC | UAAACAAUUUAUAGC | 27938 | 47 | 583 | mRNA4 |
| AUAAA | UC | UC | UAUACUUGUCGUGGC | 21750 | 65 | 422 | mRNA2 |
| GAGAA | CC | UC | UAAUCUAAACUUUAA | 29653 | 12 | 140 | mRNA7 |
| GAGAA | CC | CC | UAAUCUAAACUUUAA | 29653 | 8 | 90 | mRNA7 |
| UCAAC | UC | UC | UAAAACUCUUGUAGU | 41 | 8 | 60 |  |
| UACCC | UC | UC | UCAACUCUAAAACUC | 34 | 5 | 45 |  |
| AAACA | CC | CC | UACUUCUUCAGACAU | 22483 | 13 | 43 |  |
| AUAUA | UC | UC | CAAGCAACUUAGUGA | 27106 | 3 | 42 |  |
| GUUUA | UG | UG | UAAUACUUUGGUGCU | 28847 | 7 | 27 |  |
| GUGAG | UC | UC | CUGGACUUUGCAUUU | 27315 | 4 | 25 |  |
| CCUAA | UC | UC | UAAACCUUAAGGAUG | 29658 | 3 | 19 | mRNA7^1^ |
| AGUGU | AA | UC | UAAGCUUAUUAUUUU | 22582 | 3 | 15 | HE^3^ |
| GAGAA | CC | CC | CACUUAAUUACUUUG | 25944 | 3 | 15 |  |
| UUGGA | UC | UC | UAAAUUAGAAUUGGU | 30653 | 3 | 12 |  |
| CCUAA | UC | UC | UAAACUUUAAGGAUU | 29658 | 3 | 12 | mRNA7^1^ |
| CCUAA | UC | UC | UAAACUUAAGGAUGU | 29658 | 6 | 11 | mRNA7^2^ |

^1^ 1-nt mismatch

^2^ 1-nt deletion

^3^ Corresponds to the HE mRNA (a.k.a. mRNA2b) observed for some strains of MHV
