# Supplementary material for: High-Resolution Analysis of Coronavirus Gene Expression by RNA Sequencing and Ribosome Profiling
Source: PLoS Pathog. 2016 Feb 26;12(2):e1005473. doi: 10.1371/journal.ppat.1005473 (PMC4769073; doi:10.1371/journal.ppat.1005473)
Supplement: S4 Table — Note fwd indicates forward primer, and rev indicates reverse primer. (DOCX) [file ppat.1005473.s004.docx]

**S4 Table. List of oligonucleotides used.** Note *fwd* indicates forward primer, and *rev* indicates reverse primer.

| **Name** | **Sequence (5′-3′)** |
| --- | --- |
| MHV frameshift signal *fwd* | GCGCCTCGAGTTCAAAAGACACGAACTTTTTAAACGGATTC |
| MHV frameshift signal *rev* | GCGCAGATCTATTAGCATTACAAATGTCAAATGCCC |
| MHV frameshift signal IFC *fwd* | TTCGGGGTACAAGTGGTAAATGCCCGTCTTG |
| MHV frameshift signal IFC *rev* | CAAGACGGGCATTTACCACTTGTACCCCGAA |
| N prot *fwd* | GCGCGGATCCCACCATGTCTTTTGTTCCTGGGCAAGAAAATGC |
| N prot *rev* | GCGCCTCGAGTTATTACACATTAGAGTCATCTTCTAACCCATC |
| nsp3 prot (pCDNA.3) *fwd* | GCGCGGATCCCACCATGGGCAAGAAAGTCGAGTTTAACGAC |
| nsp3 prot (pCDNA.3) *rev* | GCGCCTCGAGTTATTAGGCCTTAATAATGGGTTTTGTATAATATTTACC |
| nsp3 Pause Control (pCDNA.3) *rev* | GCGCCTCGAGTTATTAACTACATGCATTCGTCACAAACTTTAC |
| nsp3 prot (pPS0) *fwd* | GCGCCTCGAGGTGATAGAGAAGTGTCAGGTGACC |
| nsp3 prot (pPS0) *rev* | GCGCCAGCTGTGAGACAAAGCAAGATTCACTAAAAAGC |
| nsp3 Pause control (pPS0) *rev* | GCGCCAGCTGTTATTACACAAACTTTACATCACGGCACAAATTTTTGGC |
| nsp3 Mut1 *fwd* | TTGTGCCGTGATGTAGCGGCTGTGACGAATGCATG |
| nsp3 Mut1 *rev* | CATGCATTCGTCACAGCCGCTACATCACGGCACAA |
| nsp3 Mut2 *fwd* | GCCAAAAATTTGTGCGCTGATGTAGCGGCTGT |
| nsp3 Mut2 *rev* | ACAGCCGCTACATCAGCGCACAAATTTTTGGC |
| nsp3 Mut3 *fwd* | TCACTTCAATTGGCCGCAAATTTGTGCGCTGA |
| nsp3 Mut3 *rev* | TCAGCGCACAAATTTGCGGCCAATTGAAGTGA |
| nsp3 Mut4 *fwd* | TCCGTTGCTGGTACCGCAGCGCTATCACTTCA |
| nsp3 Mut4 *rev* | TGAAGTGATAGCGCTGCGGTACCAGCAACGGA |
| nsp3 Mut5 *fwd* | CTCGAGGTGATAGAGGCGTGTCAGGTGACCTC |
| nsp3 Mut5 *rev* | GAGGTCACCTGACACGCCTCTATCACCTCGAG |
