# Supplementary figures and images for: High-Resolution Analysis of Coronavirus Gene Expression by RNA Sequencing and Ribosome Profiling
Source: PLoS Pathog. 2016 Feb 26;12(2):e1005473. doi: 10.1371/journal.ppat.1005473 (PMC4769073; doi:10.1371/journal.ppat.1005473)

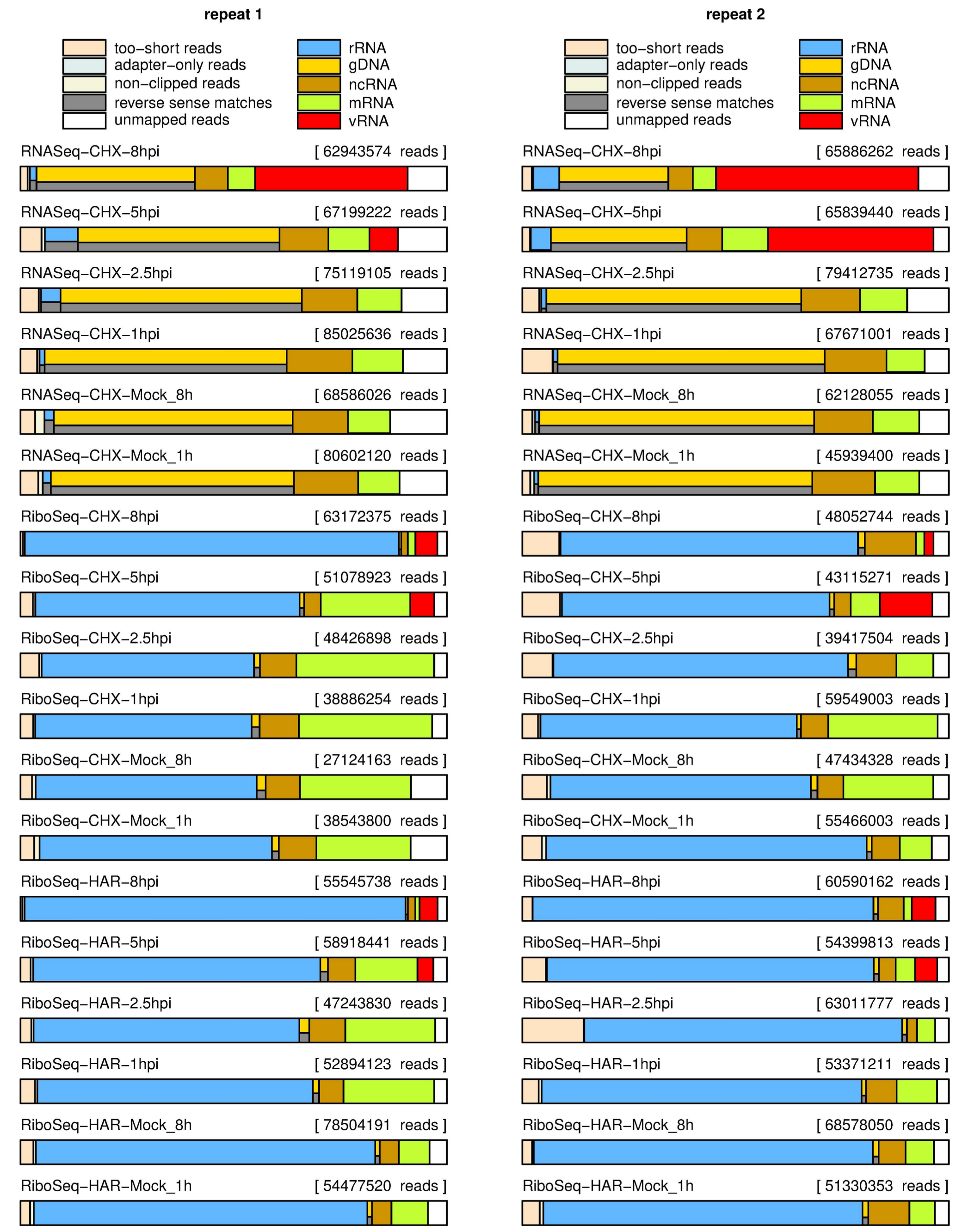

Supplement: S1 Fig — Reads were mapped to virus RNA, and host rRNA, mRNA, ncRNA and gDNA databases. Reads mapping to gDNA are expected to derive from unannotated transcripts not present in the mRNA or ncRNA databases, but, since the direction of transcription is not annotated in the gDNA database, such reads constitute a mixture of forward and reverse-sense matches. Reverse-sense rRNA matches in the RNASeq samples are expected to derive from the RiboZero kit which contains complementary sequences to rRNA. (TIF) [file ppat.1005473.s005.tif]

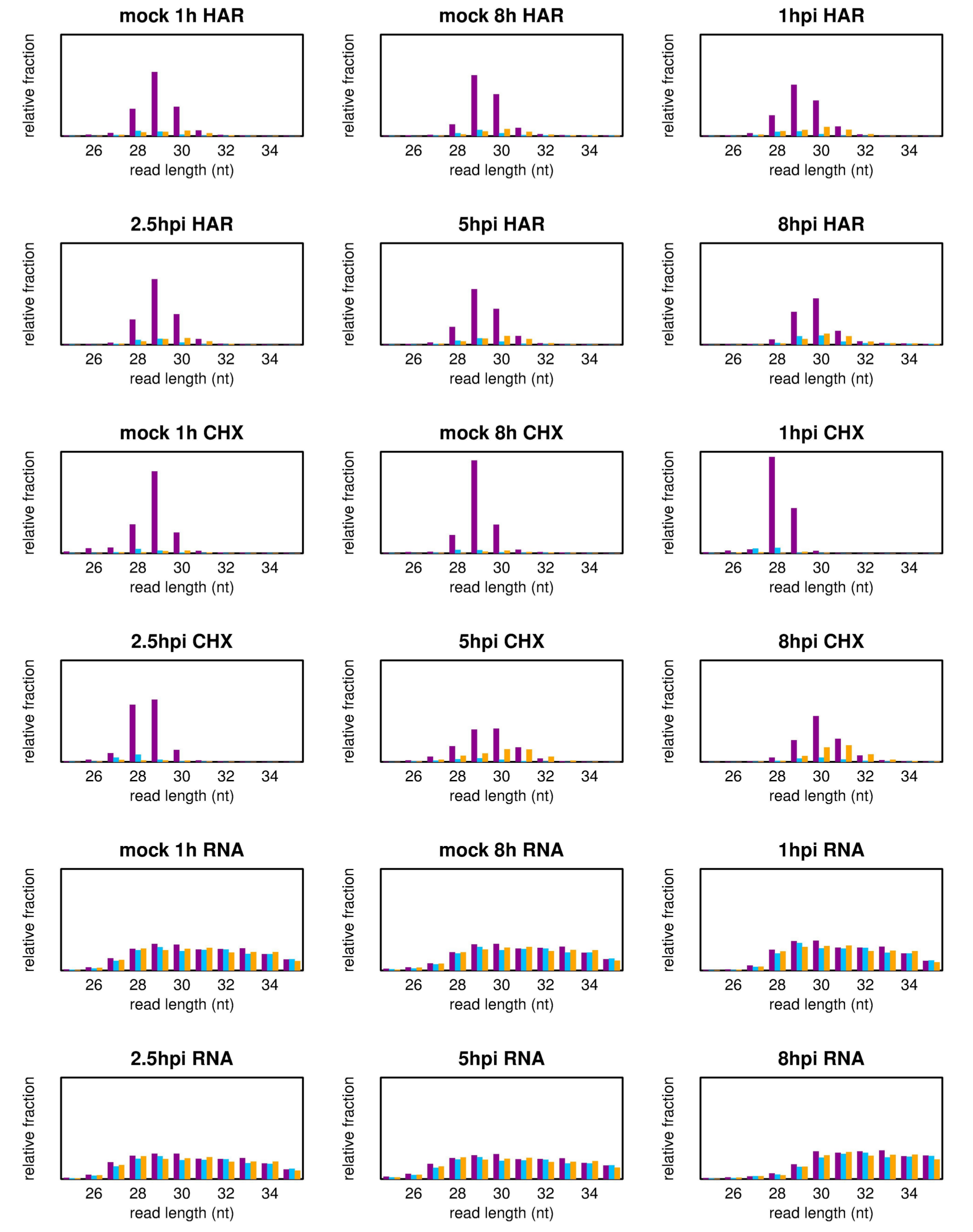

Supplement: S2 Fig — Phasing of 5′ ends of reads that map to host mRNA coding regions as a function of read length. Reads whose 5′ ends map to the first, second or third positions of codons are indicated in purple, blue or orange, respectively. (TIF) [file ppat.1005473.s006.tif]

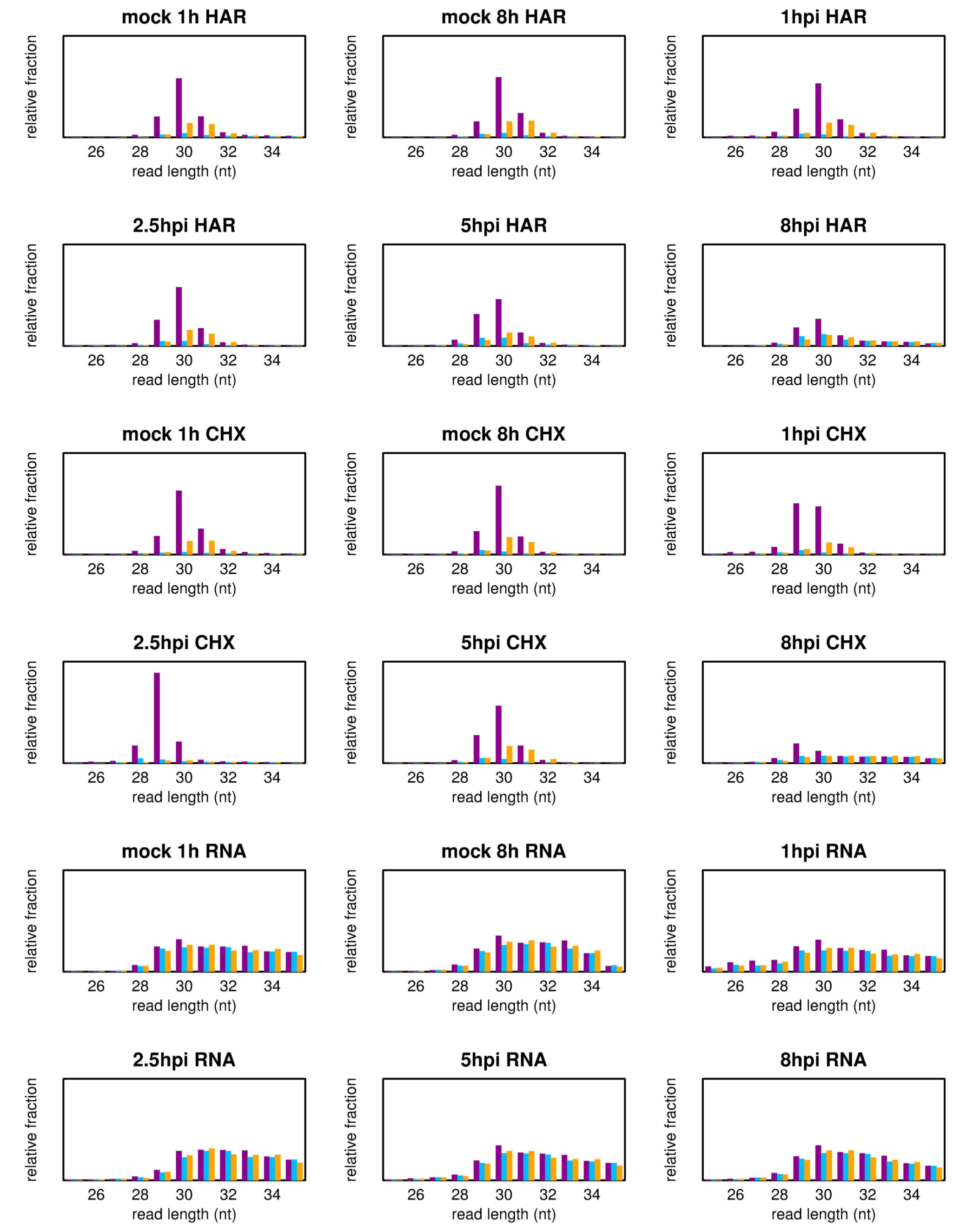

Supplement: S3 Fig — See S2 Fig caption for details. (TIF) [file ppat.1005473.s007.tif]

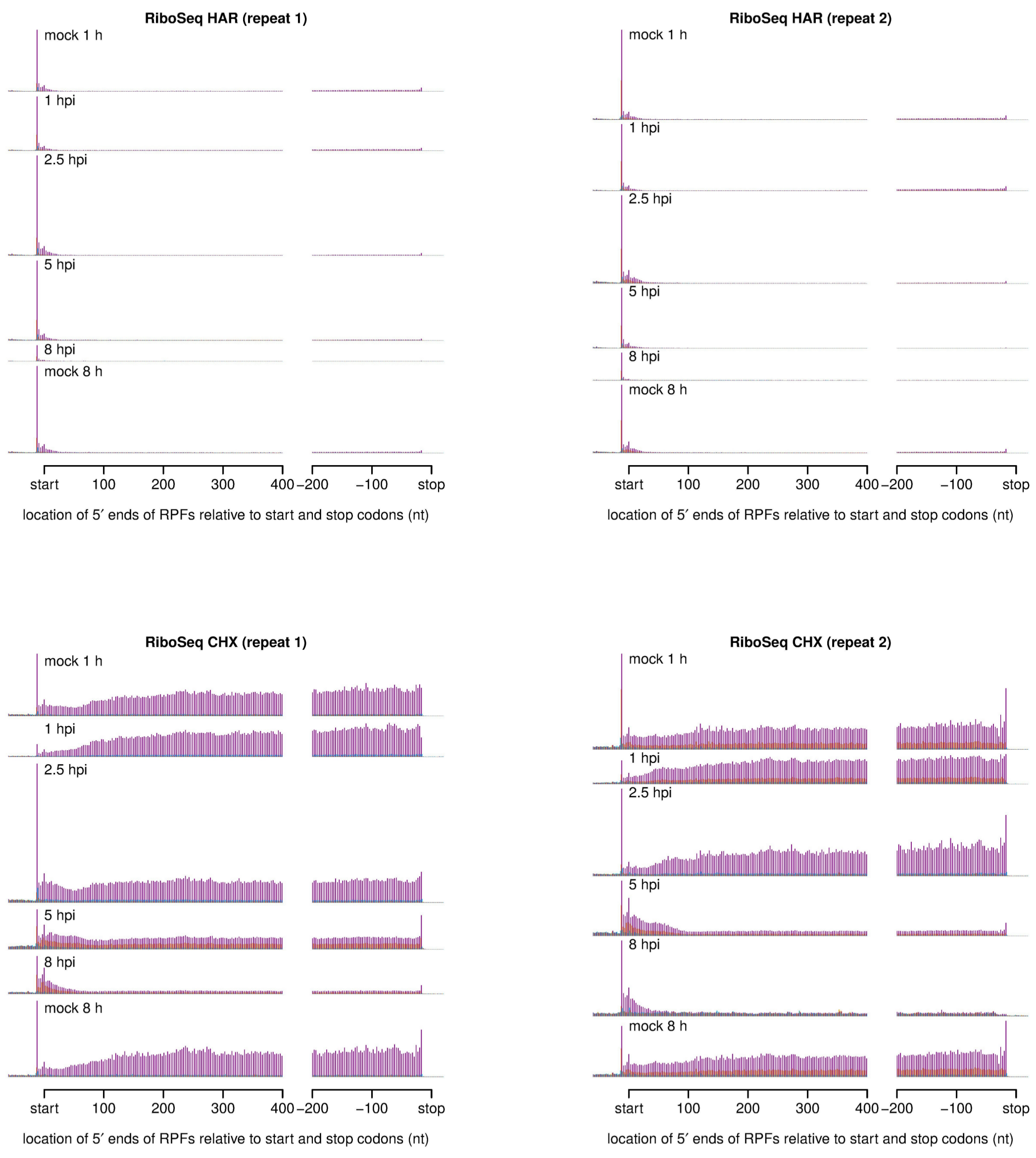

Supplement: S4 Fig — Histograms of RPF 5′ end positions relative to annotated initiation and termination codons summed over all host RefSeq mRNAs for the RiboSeq libraries. To account for different library sizes, histograms are normalized by the sum of total virus RNA (positive and negative-sense) plus total host mRNA for the library. (TIF) [file ppat.1005473.s008.tif]

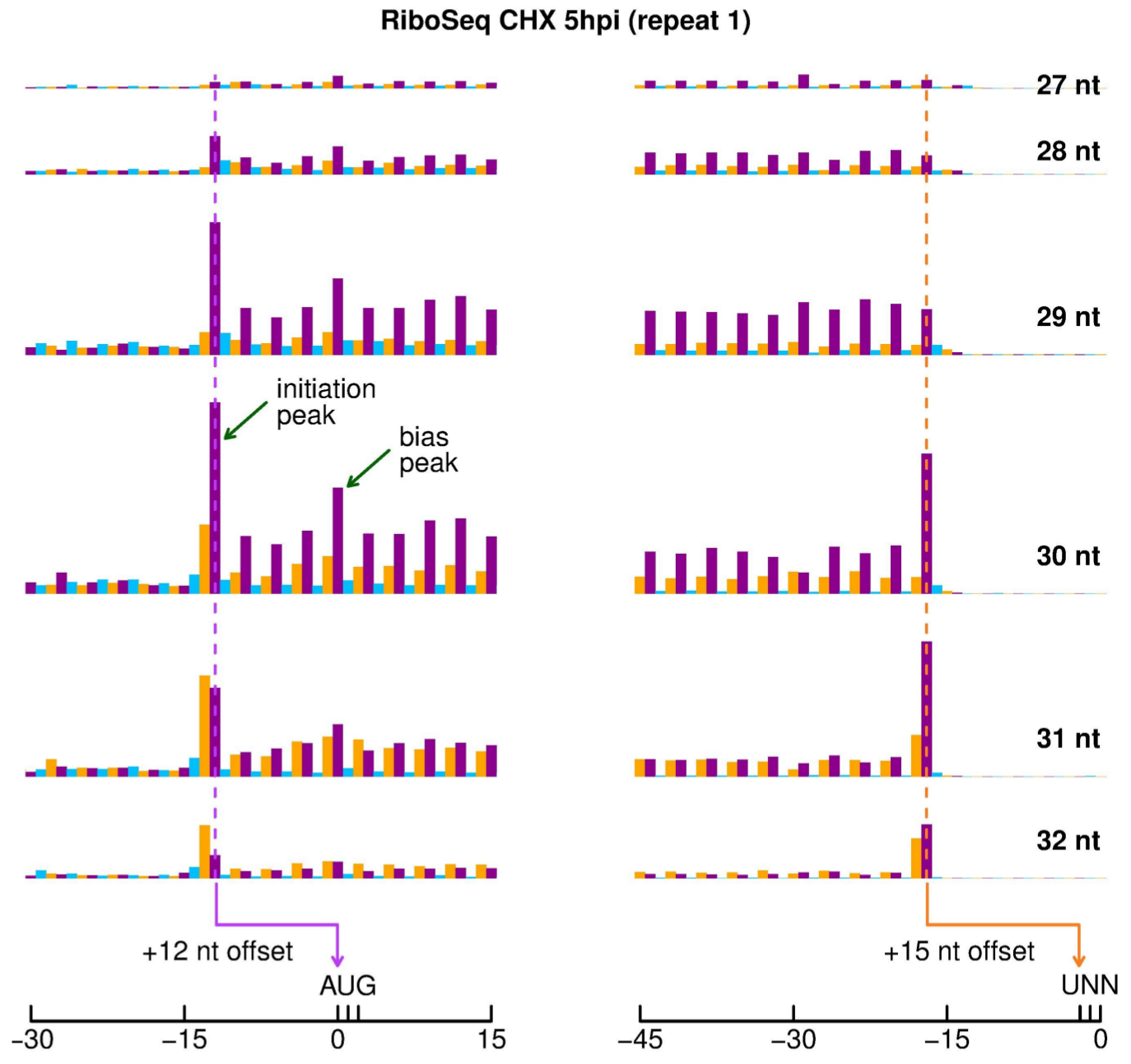

Supplement: S5 Fig — Histograms of RPF 5′ end positions relative to annotated initiation and termination codons summed over all host RefSeq mRNAs for the RiboSeq CHX 5 h p.i. time point (repeat 1) as a function of RPF length. RPFs of ribosomes paused during initiation with the initiation codon (AUG at position 0 to 2; left) in the P-site have 5′ ends that normally map to position −12 (12 nt upstream), or, particularly for longer RPFs (e.g. 30–32 nt), position −13. The smaller peak at 0 is likely an artifact of ligation bias (and potentially also nuclease bias)—all RPFs mapping to this position begin with 5′-AUG, whereas RPFs that map to other positions have differing 5′ end nucleotides so that any 5′-end-dependent biases are averaged out when summing over many mRNAs. Termination occurs with the stop codon (UNN at position −2 to 0; right) in the A-site so that the 5′ ends of RPFs paused during termination normally map to position −17 (15 nt upstream). (TIF) [file ppat.1005473.s009.tif]

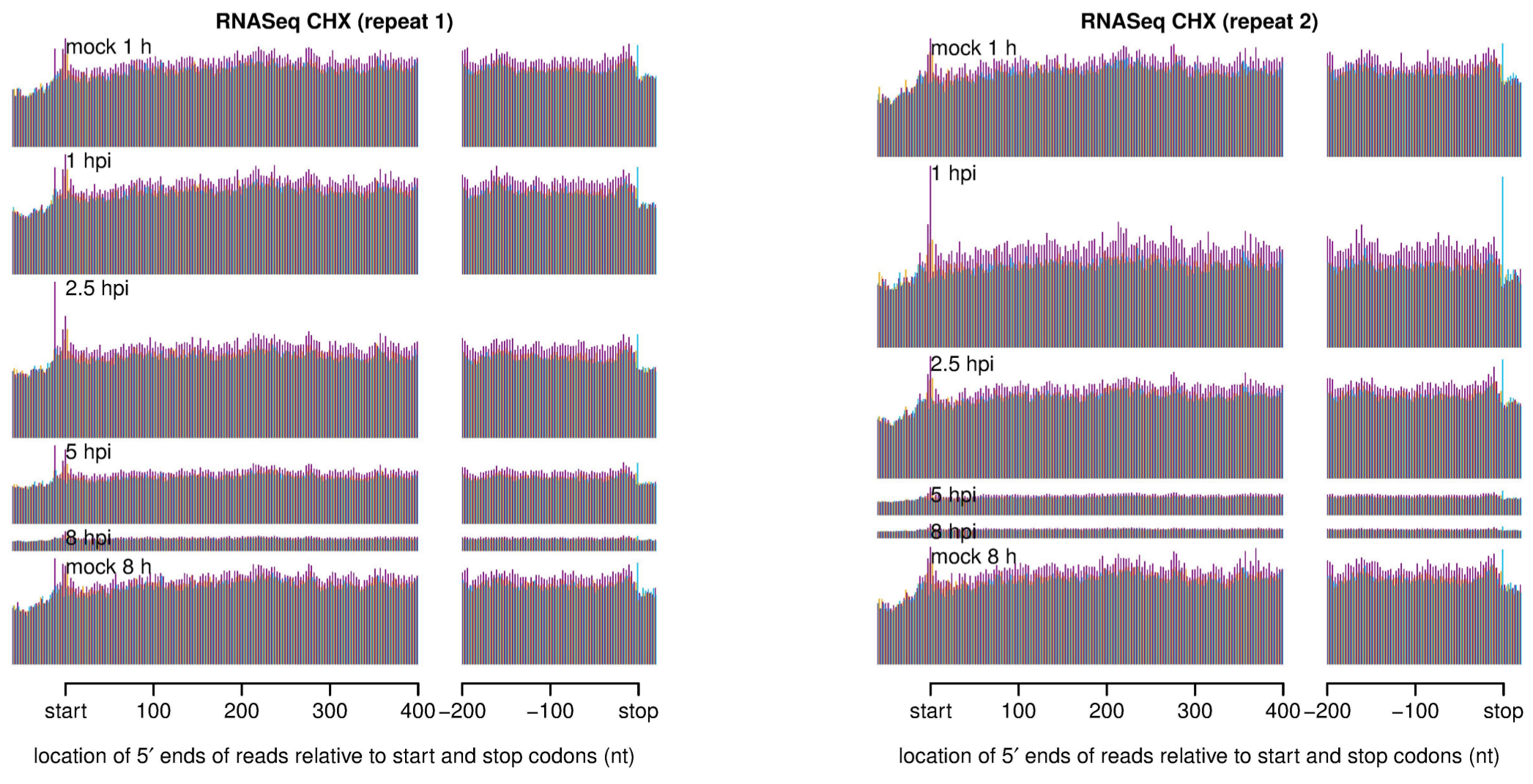

Supplement: S6 Fig — Histograms of read 5′ end positions relative to annotated initiation and termination codons summed over all host RefSeq mRNAs for the RNASeq libraries. To account for different library sizes, histograms are normalized by the sum of total virus RNA (positive and negative-sense) plus total host mRNA for the library. (TIF) [file ppat.1005473.s010.tif]

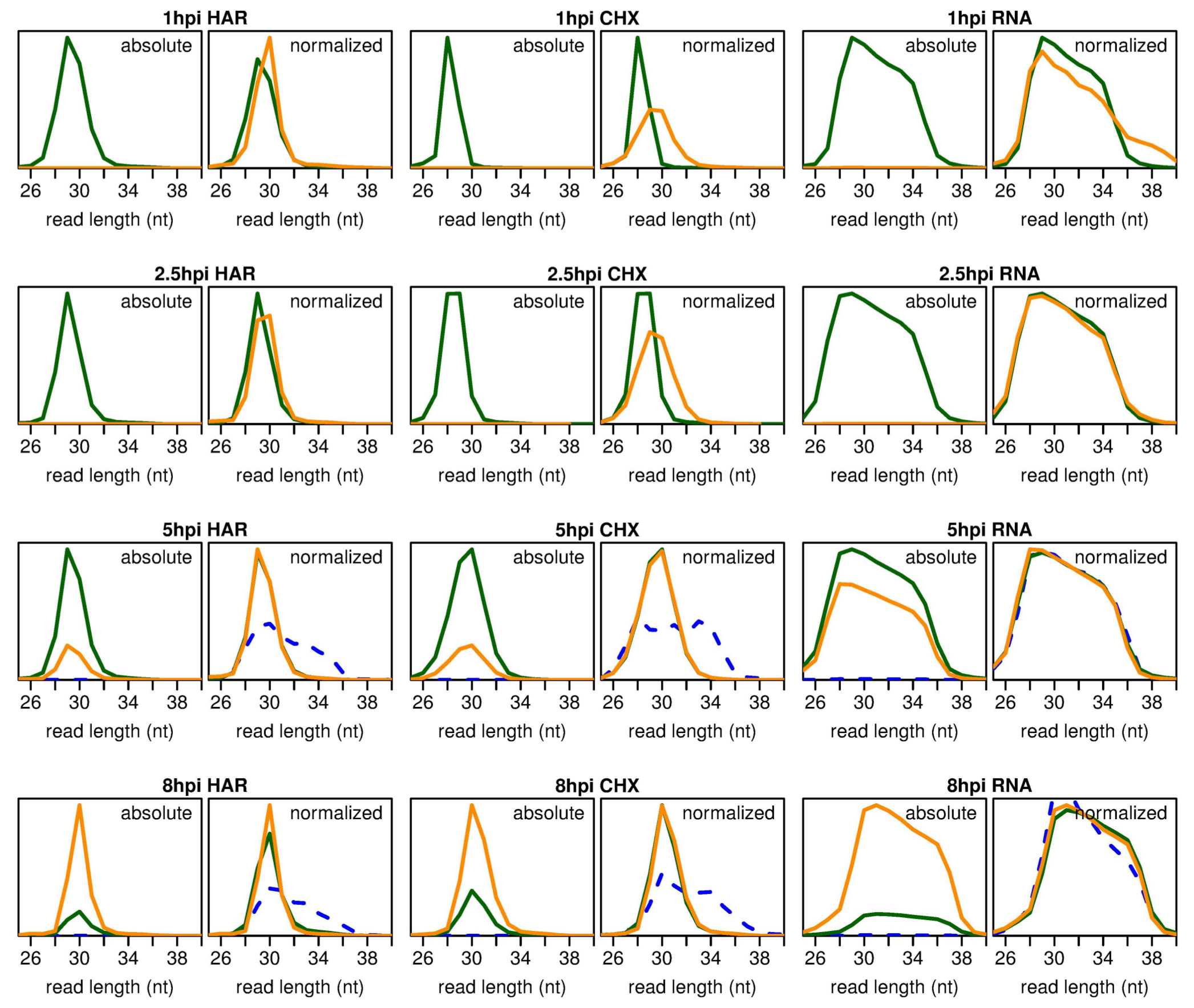

Supplement: S7 Fig — Length distributions for reads mapping to host mRNAs (green), positive-sense virus RNA (orange) and negative-sense virus RNA (blue, dashed) for repeat 1. The left panel in each pair shows the absolute read counts. The right panel in each pair shows the distributions normalized to have equal total sums to facilitate comparison of distribution shapes. Differences between host and virus distributions are indicative of contamination. Negative-sense virus read length distributions are only shown at 5 h p.i. and 8 h p.i. as the counts at earlier time points are often too low to assess distribution shape. (TIF) [file ppat.1005473.s011.tif]

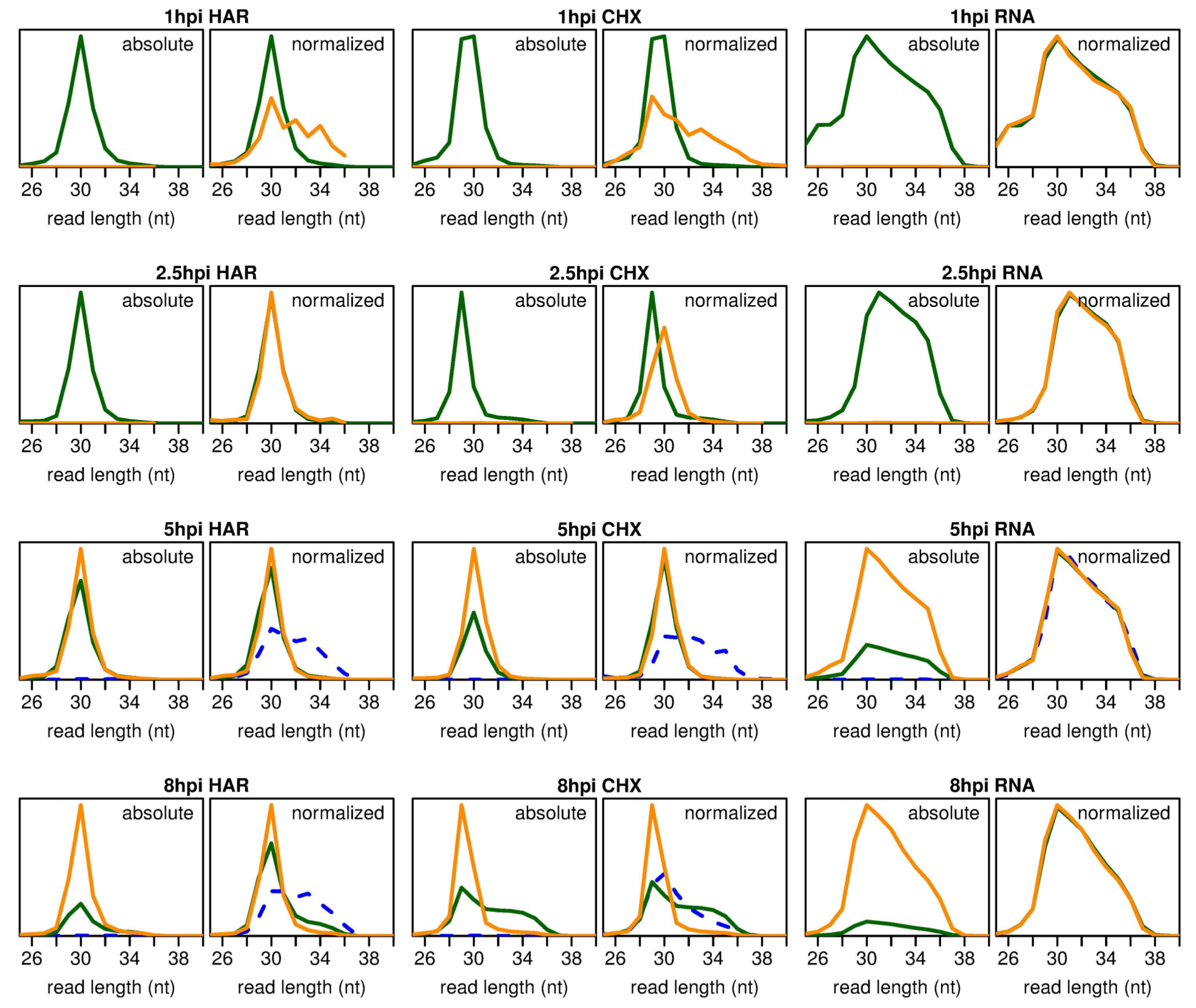

Supplement: S8 Fig — See S7 Fig caption for details. (TIF) [file ppat.1005473.s012.tif]

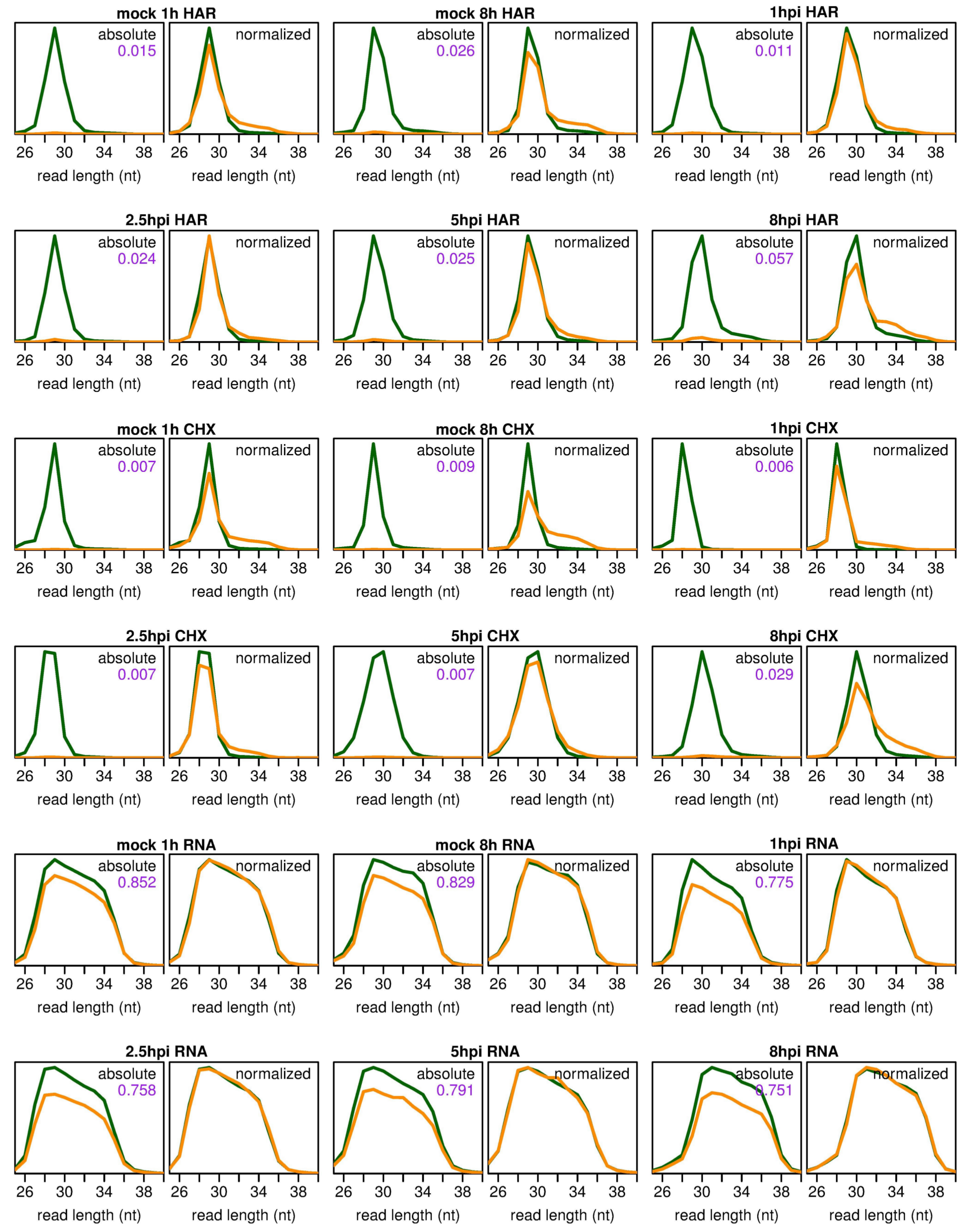

Supplement: S9 Fig — Reads were counted in windows from 10 to 100 codons upstream (CDS; green) or downstream (3′ UTR; orange) of annotated termination codons, and summed over all host mRNAs. The left panel in each pair shows the absolute read counts, allowing comparison of the CDS and 3′ UTR read densities; the density ratio (3′ UTR / CDS) is indicated in purple in each panel. For all RiboSeq samples, 3′ UTR occupancy is very low compared to CDS occupancy, whereas, for RNASeq, 3′ UTR occupancy is typically around 80% of CDS occupancy (the RNASeq value is less than unity due to differences in the transcript isoforms present in the sample compared to the RefSeq mRNA database). The right panel in each pair shows the distributions normalized to have equal total sums so that the shapes of the CDS and 3′ UTR distributions can be compared. For RNASeq, the two distributions have essentially identical shapes. For RiboSeq, differences in the two distributions provide an indicator of the level of non-RPF contamination present in the sample. (TIF) [file ppat.1005473.s013.tif]

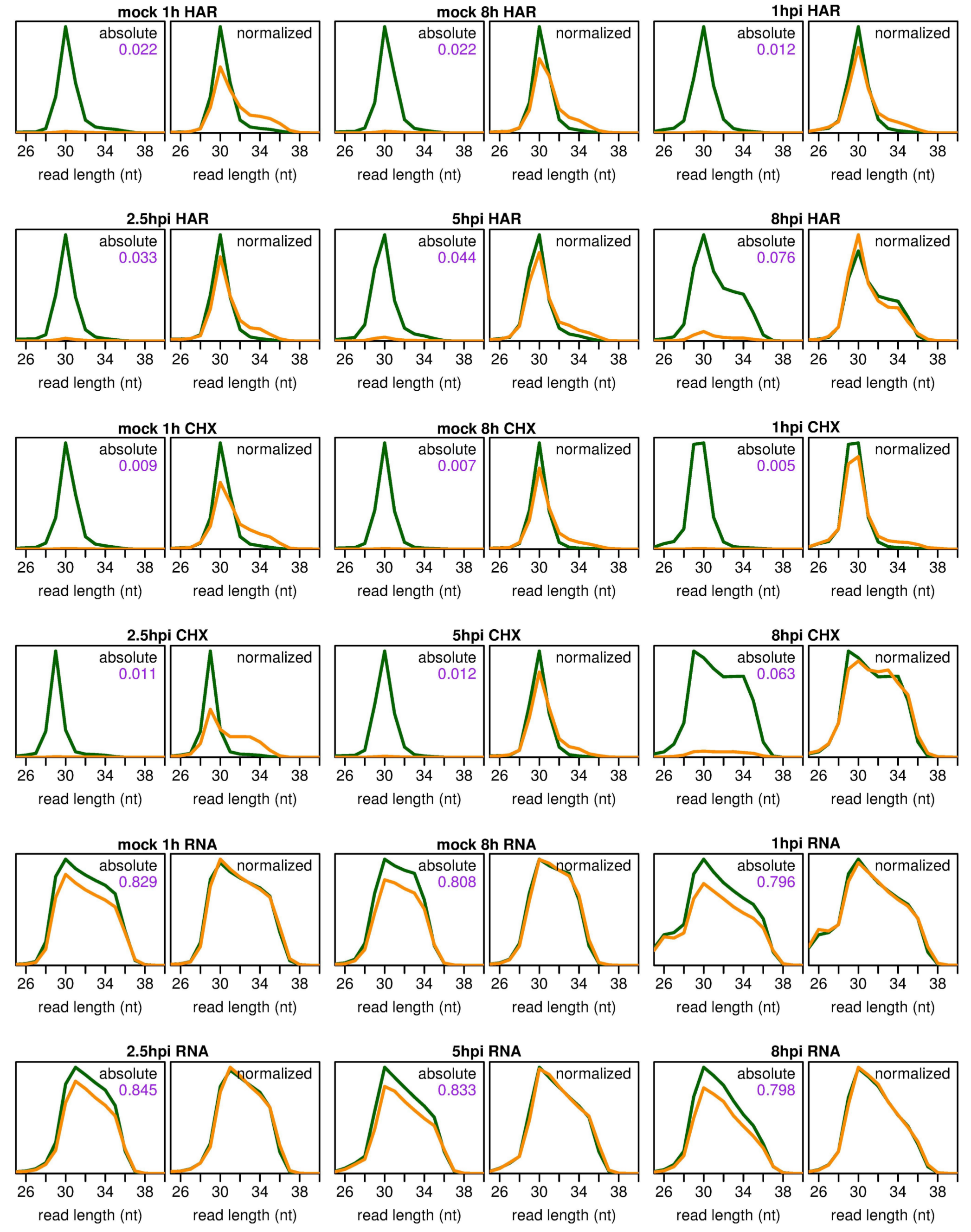

Supplement: S10 Fig — See S9 Fig caption for details. (TIF) [file ppat.1005473.s014.tif]

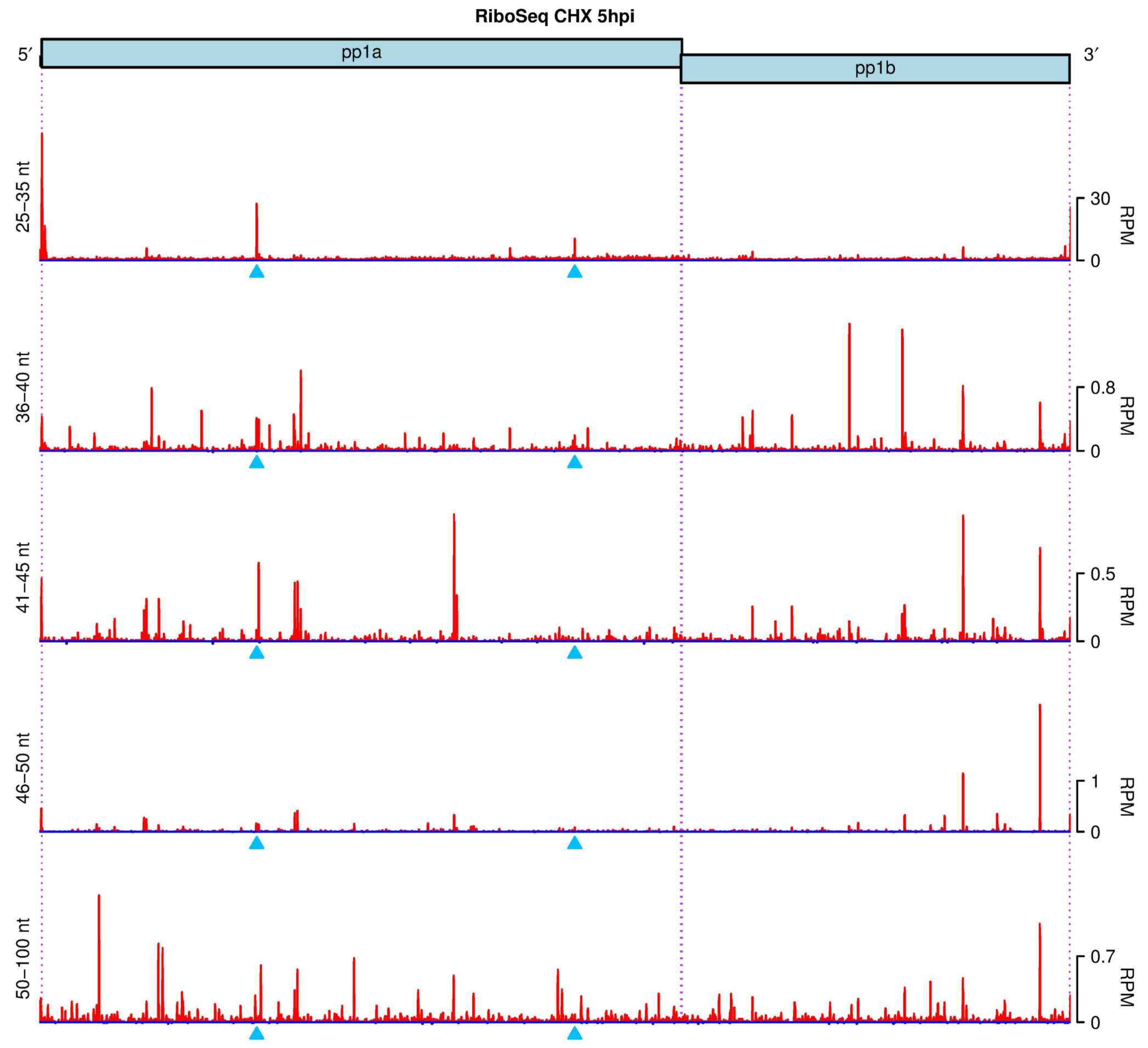

Supplement: S11 Fig — Unfractionated RiboSeq RNA prepared for the 5 h p.i. time point of repeat 2 was re-run on a 15% denaturing acrylamide-urea gel and a larger gel slice taken to sample RPFs within the range 28 to ~80 nt. Histograms show the positions of the 5′ ends of reads with a +12 nt offset to map the approximate P-site. RPF distributions were smoothed with a 15-nt running-mean filter. Note the widely varying vertical axis scales—the vast majority of RPFs fall in the size range 25–35 nt. Blue triangles indicate the previously analysed sites of RPF accumulation (see Fig 9). (TIF) [file ppat.1005473.s015.tif]

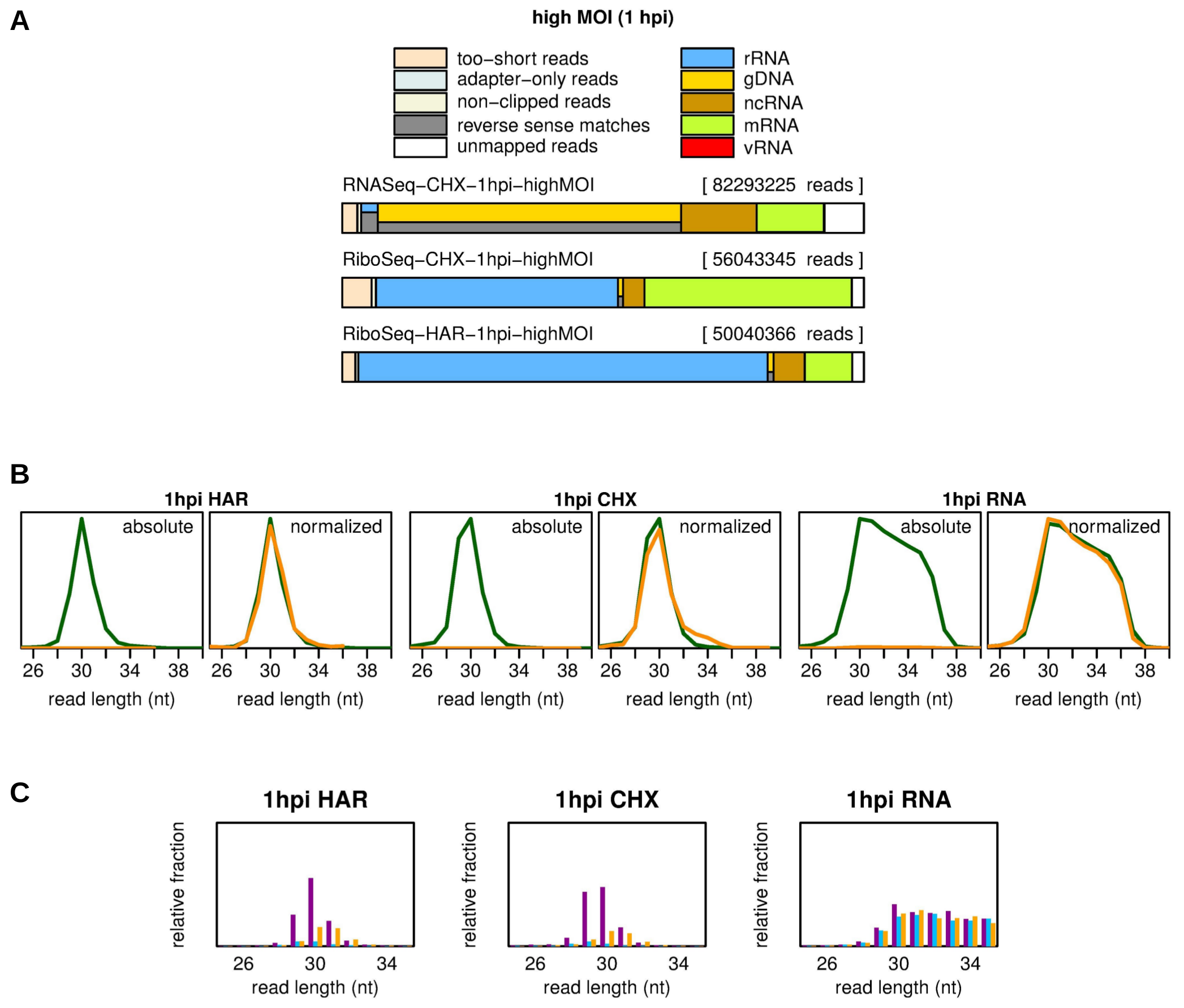

Supplement: S12 Fig — (A) Composition of the high MOI libraries (see S1 Fig caption for further details). (B) Comparison of read length distributions for virus and host mRNA (see S7 Fig caption for further details). (C) Phasing of reads mapping to host mRNAs (see S2 Fig caption for further details). (TIF) [file ppat.1005473.s016.tif]
